# Hypoxia and loss of *GCM1* expression prevent differentiation and contact inhibition in human trophoblast stem cells

**DOI:** 10.1016/j.stemcr.2025.102481

**Published:** 2025-04-24

**Authors:** Jessica K. Cinkornpumin, Sin Young Kwon, Anna-Maria Prandstetter, Theresa Maxian, Jacinthe Sirois, James Goldberg, Joy Zhang, Deepak Saini, Purbasa Dasgupta, Mariyan J. Jeyarajah, Stephen J. Renaud, Soumen Paul, Sandra Haider, William A. Pastor

**Affiliations:** 1 Department of Biochemistry, McGill University, Montreal, QC, Canada; 2 Placental Development Group, Reproductive Biology Unit, Medical University of Vienna, Vienna, Austria; 3 The Rosalind & Morris Goodman Cancer Institute, McGill University, Montreal, QC, Canada; 4 Department of Anatomy and Cell Biology, Schulich School of Medicine and Dentistry, University of Western Ontario, London, ON, Canada; 5 Department of Pathology and Laboratory Medicine, University of Kansas, Kansas City, Kansas, USA; 6 Institute for Reproduction and Developmental Sciences, University of Kansas, Kansas City, Kansas, USA; 7 Department of Obstetrics and Gynecology, University of Kansas, Kansas City, Kansas, USA

**Keywords:** placenta, cytotrophoblast, trophoblast stem cell, extravillous trophoblast, syncytiotrophoblast, GCM1, CDKN1C, differentiation, placental villi, cell column, hypoxia

## Abstract

During the first stages of embryonic development, the placenta develops under very low oxygen tension (∼1%–2% O_2_), so we sought to determine the regulatory role of oxygen in human trophoblast stem cells (hTSCs). We find that low oxygen promotes hTSC self-renewal but inhibits differentiation to syncytiotrophoblast (STB) and extravillous trophoblast (EVT). The transcription factor GCM1 (glial cell missing transcription factor 1) is downregulated in low oxygen, and concordantly, there is substantial reduction of GCM1-regulated genes in hypoxic conditions. Knockout of GCM1 in hTSC likewise impaired EVT and STB formation. Treatment with a phosphatidylinositol 3-kinase (PI3K) inhibitor reported to reduce GCM1 protein levels likewise counteracts spontaneous or directed differentiation. Additionally, chromatin immunoprecipitation of GCM1 showed binding near key genes upregulated upon differentiation including the contact inhibition factor *CDKN1C.* Loss of *GCM1* resulted in downregulation of *CDKN1C* and corresponding loss of contact inhibition, implicating GCM1 in regulation of this critical process.

## Highlights

• Low oxygen prevents spontaneous and directed differentiation of hTSCs
• Genes negatively regulated by hypoxia are often positively regulated by GCM1
• Loss of GCM1 or inhibition of PI3K inhibits differentiation
• GCM1 positively regulates *CDKN1C* and contact inhibition

## Graphical abstract

**Figure:**
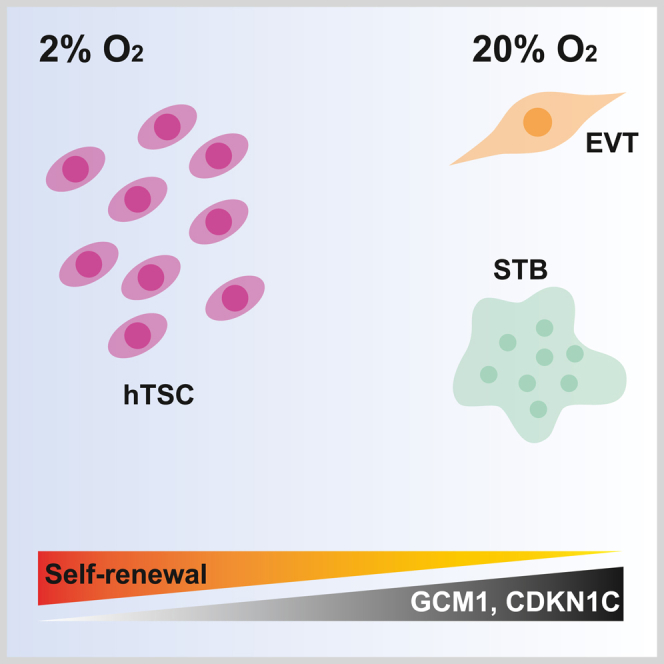

## Teaser

The placenta initially develops under low oxygen tension. Here, Pastor and colleagues show that low oxygen promotes the self-renewal of placental stem cells and inhibits the expression of the transcription factor GCM1. GCM1 in turn promotes differentiation and positively regulates the imprinted cell cycle inhibitor *CDKN1C.*

## Introduction

During the first major specification event in embryonic development, the outer cells of the developing blastocyst are specified as trophectoderm (TE) (Chazaud and Yamanaka, 2016). Cells from the TE, upon implantation, give rise to cells called cytotrophoblasts (CTBs), which can differentiate into the extravillous trophoblast (EVT) and syncytiotrophoblast (STB). These cells are organized into structures called villi, in which CTBs line the inside of the villus and STBs line the outside, mediating the exchange of nutrients, oxygen (O_2_), and waste. At the tips of the villi, the points of contact with maternal tissue, the CTBs form a cell column and differentiate into EVTs. Distinct subtypes of mature EVTs act to invade maternal decidua and to remodel spiral arteries, enabling proper blood flow to the placenta (Turco et al., 2018).

During the first trimester of human pregnancy, some EVTs establish plugs blocking the uterine spiral arteries. For the initial stages of the first trimester, the conceptus develops in a low-oxygen environment (∼2.5% O_2_, hypoxia) (Jauniaux et al., 2001; Rodesch et al., 1992). After approximately 8 weeks, the trophoblast plugs disintegrate, and endovascular extravillous cytotrophoblasts invade the uterine spiral arteries where they degrade smooth muscle and replace the resident endothelial cells (Sato, 2020). This expands the arterial lumen, provides blood to the placenta, and raises oxygen tension (∼8.6% O_2_) (Chang et al., 2018; Genbacev et al., 1997; Rodesch et al., 1992).

Oxygen tension is clearly important in regulation of the trophoblast, but how it regulates placental cell self-renewal and differentiation is not entirely clear. Aspects of response to hypoxia are universal. At high oxygen levels, one of several prolyl hydroxylases will oxidize the hypoxia-inducible factor (HIF) family transcription factors, HIF1α and HIF2α. The oxidized prolines are then recognized by von Hippel-Lindau tumor supressor (VHL), which ubiquitinates the HIFs and targets them for destruction (Majmundar et al., 2010). Low oxygen reduces the activity of the prolyl hydroxylases and thus stabilizes HIF family transcription factors. HIF1α or HIF2α then dimerizes with the transcription factor aryl hydrocarbon receptor nuclear translocator (ARNT) and promotes transcription of target genes. Certain HIF targets are consistent across cell types, and hypoxia frequently promotes angiogenesis and a shift from oxidative respiration to glycolysis (Majmundar et al., 2010). With regard to placenta, mice carrying deletions of *Hif1α/Hif2α* or *Arnt* both undergo midgestational embryonic lethality (Cowden Dahl et al., 2005; Kozak et al., 1997). These mutants show reduced labyrinth vascularization, consistent with the classic role for hypoxia signaling in angiogenesis (Cowden Dahl et al., 2005; Kozak et al., 1997). Intriguingly, they also fail to maintain their spongiotrophoblast population (the murine functional equivalent to EVT precursors), and *Hif1a*^−/−^*Hif2a*^−/−^ or *Arnt*^−/−^ murine trophoblast stem cells preferentially differentiate to STB rather than spongiotrophoblast lineage (Cowden Dahl et al., 2005). Loss of the prolyl hydroxylase *Phd2*, which causes elevated HIF stability, results in reduced expression of STB markers and an increase in spongiotrophoblast (Takeda et al., 2006), while a *Vhl*^−/−^ mouse features a loss of STB altogether (Gnarra et al., 1997). Thus, a consistent feature in murine models is that hypoxia is unfavorable to STB differentiation but conducive for spongiotrophoblast.

In humans, hypoxia is well established to block differentiation to STBs (Alsat et al., 1996; Jaremek et al., 2023; Nelson et al., 1999; Wakeland et al., 2017). The effects on EVT differentiation are less clear. When explants of human placental villi are cultured in high oxygen, expression of integrin subunit alpha 1 (ITGA1, a mature EVT marker) is observed at the edges of the explant where cell column CTBs are found. In low oxygen, instead of ITGA1 expression, cell column CTBs proliferated and appeared to show elevated human leukocyte antigen (HLA)-G (Genbacev et al., 1997). Low O_2_ is reported to reduce CTB invasiveness and block the expression of ITGA1, further supporting a role for oxygen in positive regulation of EVT differentiation (Genbacev et al., 1996). Another study reports higher HLA-G upon culture of human CTB at low O_2_ and indicates a positive role for hypoxia in promoting conversion of CTB to less mature, proximal column EVT (Wakeland et al., 2017). With the discovery of culture conditions that allow for indefinite culture of CTBs *in vitro* as human trophoblast stem cells (hTSCs) (Okae et al., 2018), we sought to determine the molecular and phenotypic effects of oxygen concentration on human placental cells.

## Results

### Hypoxia maintains trophoblast stemness

**Figure 1:**
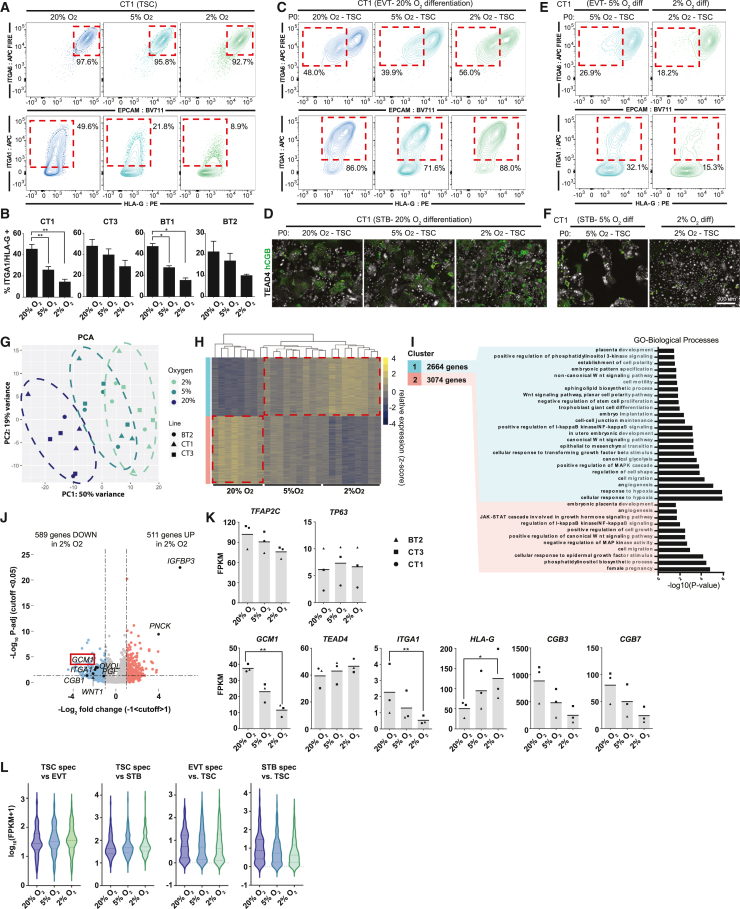
Reduced and impaired hTSC differentiation in hypoxic conditions (A) Trophoblast stem cells were cultured for 72 h in varying levels of oxygen (20%, 5%, 2% O_2_). Flow cytometry plots indicate levels of hTSC (ITGA6 and EPCAM) and EVT (ITGA1 and HLA-G) markers. Note reduction in ITGA1^+^ population in low O_2_. (B) ITGA1^+^ HLA-G^+^ population in O_2_ and cell line indicated (4 cell lines, *n* = 3 replicates for each cell line at 3 different passages). Statistical significance was determined via a two-tailed t test. (C) EVT differentiation in 20% O_2_ starting with hTSC in oxygen concentration indicated. Successful differentiation is indicated by the upregulation of surface markers ITGA1 and HLA-G and downregulation of EPCAM and ITGA6. (D) STB differentiation in 20% O_2_ starting with hTSC in oxygen concentration indicated. STB formation is indicated by loss of TEAD4 and increase in hCGB staining in a cell. (E) EVT differentiation undertaken at oxygen level indicated. (F) STB differentiation undertaken at oxygen level indicated. (G) Principal component analysis (PCA) showing gene expression from hTSC cultured in varying oxygen concentrations. Ovals encompassing all 2%, 5%, and 20% O_2_ samples are drawn manually. (H) Hierarchical gene clustering of RNA-seq samples in (G). Red dotted lines indicate the shift in gene expression from 20% O_2_ and 2% O_2_ labeled as cluster 1 and cluster 2. (I) Gene set enrichment analysis (GSEA) analysis of cluster 1 and cluster 2. (J) Volcano plot showing gene expression differences between TSCs cultured in 20% O_2_ to TSCs cultured in 2% O_2_. Dashed lines indicate significance and log2 fold change cutoff. (K) Bar graphs showing FPKM of specific genes of interest (same samples as in G, significance indicated corresponds to *p*_adj_ values from DESEQ2 analysis, see Table S1). (L) Violin plot showing expression of genes specific to hTSC, EVT, or STB for hTSCs grown in the indicated oxygen concentration. When comparing log_2_ fold change between genes in each set, differences between all sets at all oxygen concentrations are significant (*p* < 0.001) (For analysis in G–L, 3 cell lines; BT2, CT1, CT3; *n* = 3 replicates for each line in each condition over 3 passages, except BT2 at 20% O_2_*n* = 2).

To determine the effect of hypoxia on hTSC growth, we cultured hTSCs in 20%, 5%, and 2% O_2_. After 72 h of culture, we performed flow cytometry for the hTSC cell surface markers ITGA6 and EpCAM and the EVT markers ITGA1 and HLA-G (Figure 1A). We observed noticeable depletion of ITGA1 and slightly increased HLA-G expression in the lower oxygen concentrations in several hTSC lines (Figures 1A and 1B), similar to what was observed in explants by Genbacev and colleagues. Continued culture of these cells in their respective oxygen conditions resulted in near complete loss of ITGA1^hi^ expression cells in both 5% and 2% O_2_ (Figure S1A). Similarly, hTSCs cultured in 20% O_2_ showed some spontaneous expression of the STB marker hCGB, which was reduced in low oxygen (Figures S1B and S1C). Regions of dense cell-to-cell contact showed expression of the differentiation marker NOTCH1 in hTSCs at 20% O_2_ but not in lower oxygen (Figure S1D). In addition to lower expression of differentiation markers, we observed higher cell density in lower oxygen culture conditions (Figure S1E). Collectively, these results indicated that low oxygen aids in stemness and proliferation, while high oxygen promotes spontaneous differentiation.

Based on these findings, we cultured hTSCs and conducted directed differentiation at varying oxygen conditions. hTSC cultured in 20%, 5%, or 2% O_2_ successfully differentiated to EVT or STB if differentiation was undertaken at 20% O_2_ (Figures 1C and 1D). However, when we performed EVT or STB differentiation at reduced oxygen levels, we observed dramatic impairment of differentiation (Figures 1E, 1F, and S1F). EVTs differentiated in reduced oxygen failed to downregulate EpCAM or upregulate ITGA1 and HLA-G (Figure 1E), while STBs in 2% O_2_ showed a higher percentage of cells retaining the stem cell marker TEA domain transcription factor 4 (TEAD4) and a lower percentage expressing the STB marker Chorionic Gonadotropin beta chain (hCGB) (Figures 1F and S1F). Thus, oxygen promotes both spontaneous and directed differentiation of hTSCs.

### Trophoblast differentiation transcription factor, GCM1, is oxygen sensitive

We performed RNA sequencing (RNA-seq) on three hTSC lines (CT1, CT3, and BT2; female) cultured in 20%, 5%, or 2% O_2_. Principal component analysis (PCA) and a correlation matrix show a strong dependence of gene expression on oxygen tension (Figures 1G and S1G). Gene cluster and enrichment analysis (Sherman et al., 2022) indicate that the genes in both cluster 1 (genes upregulated in 2% O_2_) and cluster 2 (upregulated in 20% O_2_) showed general enrichment for various placental-related terms while only cluster 1 corresponded to hypoxia response and WNT activation (Figures 1H and 1I).

Genes associated with EVT and STB expression such as *ITGA1* and *OVOL1* and various chorionic gonadotropin genes were among the genes less expressed in the 2% O_2_ condition (Figures 1J, 1K, and S1H; Table S1). Using published gene expression data, we identified 100 genes specific to EVT and STB differentiation and 100 genes specific to hTSC relative to STB and EVT (Okae et al., 2018). hTSC genes were higher in 2% O_2_, while EVT and STB genes were lower, further indicating that hypoxia broadly suppresses genes associated with differentiation (Figure 1L; Table S2). Interestingly, consistent with flow cytometry data (Figure 1A), HLA-G was positively regulated by hypoxia (Figure 1K), indicating that hypoxia promotes expression of this differentiation marker even as it suppresses the overall EVT differentiation program.

Analysis of known transcription factor (TF) targets appropriately indicated that HIF1α was the most enriched TF associated with cluster 1 (hypoxia) expression, while *GCM1* (glial cells missing TF 1) was associated with high oxygen concentration (Figures S1I and S1J). *GCM1*, which is expressed in hTSCs, but upregulated upon differentiation (Figure S1K), was itself strongly downregulated in low oxygen conditions both at the RNA and protein level (Figures 1J, 1K, and S1L; Table S1). GATA3, reported to repress GCM1 by an indirect mechanism (Wang et al., 2022), was upregulated in 2% O_2_, but only slightly, suggesting another mechanism at work (Figure S1H). To determine if GCM1 is regulated by the canonical hypoxia pathway, we used CRISPR interference to repress *VHL*, the ubiquitin ligase that targets HIF proteins for destruction. Repression of *VHL* in 20% O_2_ led to dramatic upregulation of *IGFBP3*, the most hypoxia-responsive gene in hTSC and a known HIF1α target (Natsuizaka et al., 2012). We also observed downregulation of *GCM1* expression, confirming that *GCM1* is negatively regulated by canonical hypoxia response (Figure S1M).

### GCM1 is essential for the differentiation into trophoblast lineages

**Figure 2:**
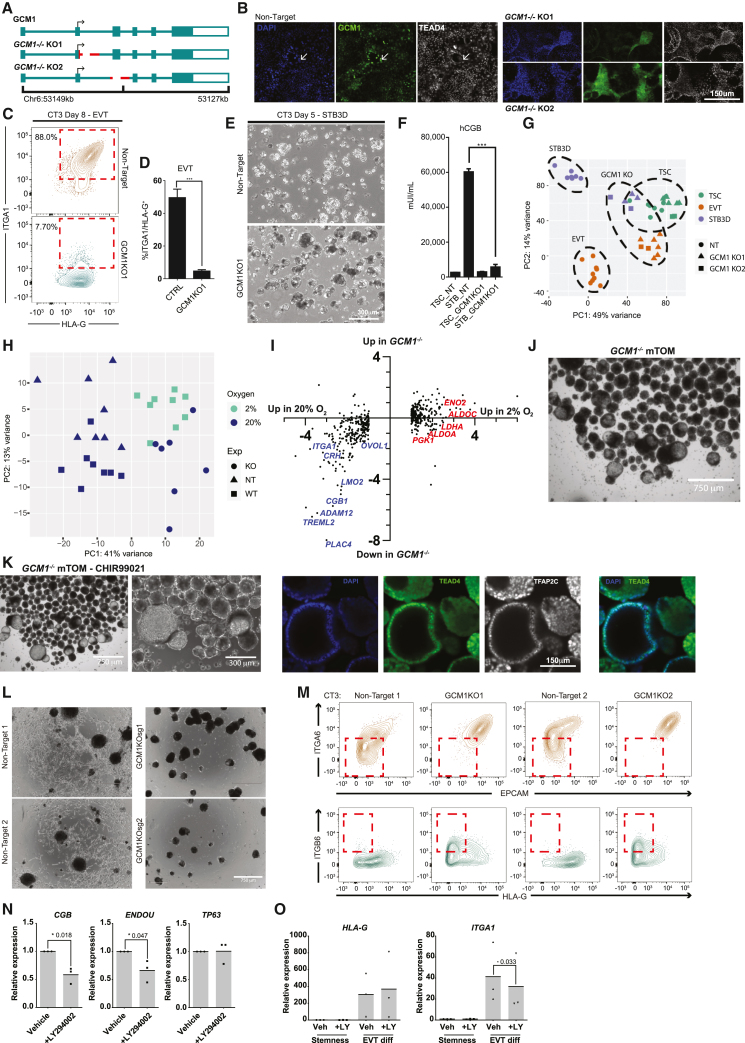
Impaired differentiation upon genetic or chemical reduction in GCM1 level (A) Strategies for mutation of GCM1 using a two-sgRNA CRISPR approach. Lines were generated by deletion of the exon2/intron2 boundary, and by ablation of exon 3, either of which should disrupt the DNA-binding domain of GCM1. (B) Immunofluorescence staining of GCM1 and TEAD4 in control (non-target, NT sgRNA) and *GCM1*^−/−^ hTSC. Sporadic GCM1^+^ TEAD4^lo^ hTSCs are present only in NT control hTSCs. (C) Flow cytometric analysis from EVT differentiation of GCM1 KO1 and NT control TSC. NT hTSC differentiation produced ITGA1^hi^/HLA-G^hi^ cells whereas *GCM1*^−/−^ TSC did not. (D) Bar graphs showing formation of ITGA1^hi^/HLA-G^hi^ population from control and *GCM1*^−/−^ TSC (2 cell lines, CT1 *n* = 2 clonal lines, CT3 *n* = 3 clonal lines for both NT and KO). (E) 3D STB formation of NT and *GCM1*^−/−^ hTSC. Control hTSCs form a fluid-filled syncytium while *GCM1*^−/−^ hTSCs form a cluster of cells. (F) hCGB ELISA was performed using supernatant from *GCM1*^−/−^ and control hTSC (2 cell lines, CT1 *n* = 2 clonal lines, CT3 *n* = 3 clonal lines for both NT and KO). Statistical significance was determined via a two-tailed t test. (G) PCA comparing NT and *GCM1*^−/−^ hTSC, EVT, and STB3D. Note that *GCM1*^−/−^ cells regardless of differentiation state cluster closer to the hTSC population, and similarity of *GCM1*^−/−^ lines 1 and 2 (TSC: *n* = 8 NT, *n* = 7 KO; EVT: *n* = 9 NT, *n* = 8 KO; STB3D *n* = 7 NT, *n* = 6 KO clonal replicates). Ovals encompassing WT TSC, STB, and EVT, as well as GCM1 KO STB and EVT, are drawn manually. (H) PCA of control (NT) and *GCM1*^−/−^ hTSCs, compared with WT hTSCs grown at different O_2_ concentrations. Note that *GCM1*^−/−^ hTSCs cluster on principal component axis 1 with WT hTSCs grown at 2% O_2_ (*n* = 8 WT 20% O_2_, *n* = 9 2% O_2_, *n* = 8 NT 20% O_2_, *n* = 7 KO 20% O_2_). (I) Scatterplot of genes differentially regulated in hypoxia (same set as Figure 1J) showing their relative expression in 2% and 20% O_2_ and their relative expression in *GCM1*^−/−^ hTSC and control cells. Examples of placental differentiation genes are shown in blue, while genes involved in glycolysis are shown in red. (J) Bright-field images of *GCM1*^−/−^ TB-ORG cultured in mTOM media. (K) Left: Bright-field images of *GCM1*^−/−^ TB-ORG culture in mTOM media-CHIR99021. Right: Immunofluorescent staining for trophoblast markers in *GCM1*^−/−^ TB-ORG (representative of *n* = 5 images for NT and KO). (L) NT and *GCM1*^−/−^ TB-ORG differentiated to EVT. (M) Flow cytometry of NT and *GCM1*^−/−^ TB-ORG differentiated to EVT. *GCM1*^−/−^ hTSCs fail to upregulate the EVT marker HLA-G but do upregulate the cell column marker, ITGB6 (representative image, *n* = 5 for NT and KO). (N) Expression of genes associated with differentiation (*CGB*, *ENDOU*) or stemness (*TP63*), normalized to the housekeeping gene *TBP*, in steady-state TB-ORG conditions with 5 μM LY294002 or vehicle control (*n* = 3 cell line replicates). Statistical significance was calculated using a one-tailed t test. (O) Expression of EVT genes upon differentiation to EVT with 5 μM LY294002 or vehicle control (*n* = 3 cell lines replicates). Statistical significance was calculated using a one-tailed t test.

Since GCM1 is highly sensitive to oxygen concentration and is implicated in hTSC differentiation (Wang et al., 2022), we generated GCM1-knockout hTSC (GCM1 KO1) by deleting a small genomic region in exon 2 just after the ATG start site to disrupt the translation of the DNA-binding domain (Chiu and Chen, 2016) and subsequent protein sequence (Figure 2A). Unexpectedly, this deletion made an alternative splice site available, which spliced in before exon 3 (Figure S2A). While this deletion still had the desired frameshift, we generated additional lines (*GCM1*^−/−^ KO2) by deleting the entirety of exon 3 (Figures 2A and S2A). hTSCs electroporated with a non-targeting single-guide RNA (sgRNA) showed ubiquitous nuclear expression of GCM1, with TEAD4 loss in the highest GCM1-expressing cells (Figure 2B), while both *GCM1*^−/−^ lines showed loss of specific GCM1 signal (Figure 2B). Consistent with recent reports (Jeyarajah et al., 2022; Shimizu et al., 2023; Wang et al., 2022), *GCM1*^−/−^ hTSC failed to differentiate to EVT or STBs, a result demonstrated by flow cytometry, immunofluorescent staining, and RNA-seq of control and *GCM1*^−/−^ cells (Figures 2C–2G and S2B–S2F). *GCM1*^−/−^ hTSCs showed lower expression of EVT and STB-specific genes and substantially failed to upregulate these genes upon directed differentiation (Figure S2G; Table S3).

We then compared the effects of *GCM1* loss to the effects of hypoxia in hTSCs. While they did not cluster precisely together, *GCM1*^−/−^ cells grown in 20% O_2_ showed similar positioning over principal component axis 1 with control hTSCs grown in 2% O_2_. This would indicate that a substantial portion of the differential gene expression associated with hypoxia is in fact a consequence of lower GCM1 level (Figure 2H). More specifically, we observe that a high proportion of genes downregulated in 2% O_2_, genes associated with trophoblast differentiation, are also downregulated in *GCM1*^−/−^. By contrast, genes upregulated in 2% O_2_, such as established HIF targets and factors that promote glycolysis (Majmundar et al., 2010), are generally unaffected in *GCM1*^−/−^ (Figure 2I).

Interesting morphological phenomena were observed for *GCM1*^−/−^ hTSCs. When *GCM1*^−/−^ lines were grown to over-confluency in hTSC media (TSCM), we observed three-dimensional dome-like projections. Much larger domes were observed in modified trophoblast organoid media with CHIR99021 omitted (mTOM-C) EVT precursor media (see methods) (Figure S2H). We then cultured the *GCM1*^−/−^ hTSCs using a modified form of a trophoblast organoid (TB-ORG) culture system. When cultured with standard mTOM media in micro-V-shaped wells with Matrigel omitted to allow free-floating organoids, the *GCM1*^−/−^ trophoblast stem cells (TSCs) formed hollow balls of cells (Figure 2J). Removal of CHIR99021 (mTOM-C) resulted in further growth of these *GCM1*^−/−^ shells (Figure 2K). At high cell number, the formation of branched structures is observed (Figure S2I). Upon directed differentiation to EVT, these *GCM1*^−/−^ TB-ORGs failed to adopt EVT morphology and enter an HLA-G^hi^ EpCAM^lo^ state. Instead they upregulated the surface marker ITGB6 (Figures 2L and 2M). ITGB6 is selectively present in column CTBs (Arutyunyan et al., 2023; Lee et al., 2018b), the last GCM1^lo^ state before differentiation, suggesting failure to differentiate beyond this stage (Figures S2J–S2L). Generally, genes upregulated in *GCM1*^−/−^ cells showed highest expression in villous and cell column CTB, while genes downregulated in *GCM1*^−/−^ were associated with subsequent differentiated states (Figure S2M).

TB-ORGs undergo spontaneous differentiation, forming a core of STB in the middle. We considered whether this could be prevented by reducing the expression of GCM1. A published report in choriocarcinoma cells showed that hypoxia inhibits the phosphatidylinositol 3-kinase (PI3K)/pAKT pathway and that chemical inhibition of PI3K can lead to reduced expression of GCM1 (Chiang et al., 2009). hTSCs treated with the PI3K inhibitor LY294002 showed some formation of dome-like structures akin to what is observed for *GCM1*^−/−^ (Figures S2N and S2O). Likewise, LY294002-treated TB-ORG grown in mTOM-C conditions without Matrigel showed some propensity for the formation of hollow cavities, though not to the same extent as *GCM1*^−/−^ (Figure S2P). TB-ORG generated from primary CTBs and treated with LY294002 at standard steady-state conditions with Matrigel showed reduced expression of the STB markers *CGB* and *ENDOU* (Figure 2N) and a modest reduction in *ITGA1* expression upon EVT differentiation while HLA-G levels were not affected (Figure 2O), suggesting that chemical modulation of GCM1 level hampers certain steps of STB and EVT differentiation.

### GCM1 positively regulates EVT and STB-specific regulators

**Figure 3:**
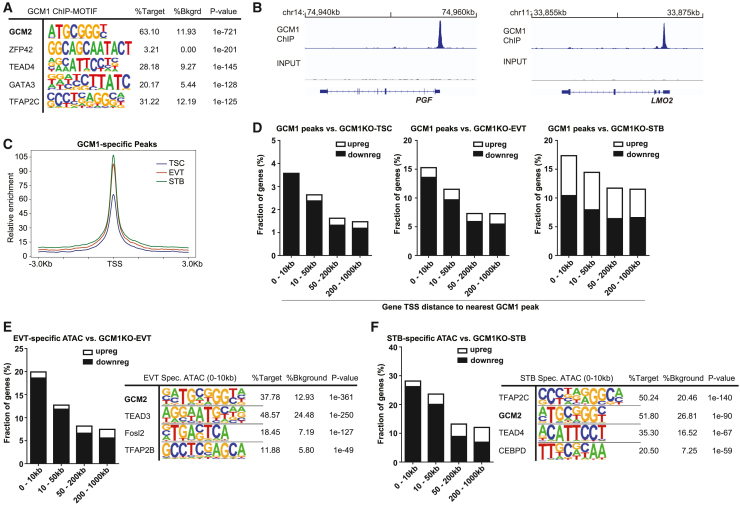
GCM1 positively regulates differentiation-associated genes (A) Motif analysis of GCM1-binding sites shows very strong enrichment for GCM motif, indicating successful and specific ChIP. (B) GCM1 enrichment over *PGF* (left) and *LMO2* (right). (C) ATAC-seq enrichment in TSC, EVT, and STB over GCM1-binding sites. (D) Plot showing the percentage of genes whose promoters are within a given distance of a GCM1-binding site that show upregulation or downregulation in *GCM1*^−/−^ cells. (E) Plot showing the percentage of genes whose promoters are within a given distance of an EVT-specific ATAC-seq site that show upregulation or downregulation in *GCM1*^−/−^ cells (left), motif analysis for EVT-specific peaks (right). (F) Plot showing the percentage of genes whose promoters are within a given distance of an STB-specific ATAC-seq site that show upregulation or downregulation in *GCM1*^−/−^ cells (left), motif analysis for STB-specific peaks (right).

We performed chromatin immunoprecipitation (ChIP) sequencing (ChIP-seq) for GCM1 from day 3 EVTs (CT3), a time point which we found was conducive to high-quality ChIP data. We identified 2,271 peaks with >4-fold enrichment over input. Motif analysis of these sites showed extremely strong enrichment for the GCM-binding motif, with weaker enrichment for other TFs common in trophoblast (Figure 3A). We confirmed enrichment at known GCM1 targets such as *PGF* and *LMO2* (Chen et al., 2022; Jeyarajah et al., 2022; Li and Roberson, 2017) (Figure 3B). We also conducted an assay for transposase-accessible chromatin (ATAC-seq) to measure open chromatin regions in TSC, EVT, and STB, which accorded well with published H3K27Ac ChIP-seq data from these cells (Figure S3A). Comparison with the ATAC-seq data showed that GCM1 peaks corresponded to regions that show higher openness in EVT and STB than in TSC (Figures 3C and S3B; Table S4). Furthermore, we used region-associated differentially expressed gene analysis (Guo et al., 2021) to correlate the proximity of a GCM1 peak to the transcription start site (TSS) of genes dysregulated upon GCM1 knockout. Genes downregulated in *GCM1*^−/−^ cells were found in proximity to GCM1 ChIP-seq peaks (Figure 3D). Putative direct targets, genes proximal to GCM1 peaks and downregulated in *GCM1*^−/−^ cells, include known placenta differentiation factors such as *PGF*, *LMO2*, *CGA*, *OVOL1*, *SYDE1*, and a range of *CGB* genes (Figure S3C; Table S4).

Further supporting the role of GCM1 in regulating differentiation, ATAC-seq analysis of TSC, EVT, and STB data showed enrichment of the GCM motif in EVT and STB-specific regions of open chromatin (Figures 3E, 3F, S3D, and S3E). We also observed the association of EVT and STB-specific ATAC-seq peaks with genes downregulated in *GCM1*^−/−^ cells of the corresponding cell types (Figures 3E and 3F).

### GCM1 regulates CDKN1C and trophoblast overgrowth

**Figure 4:**
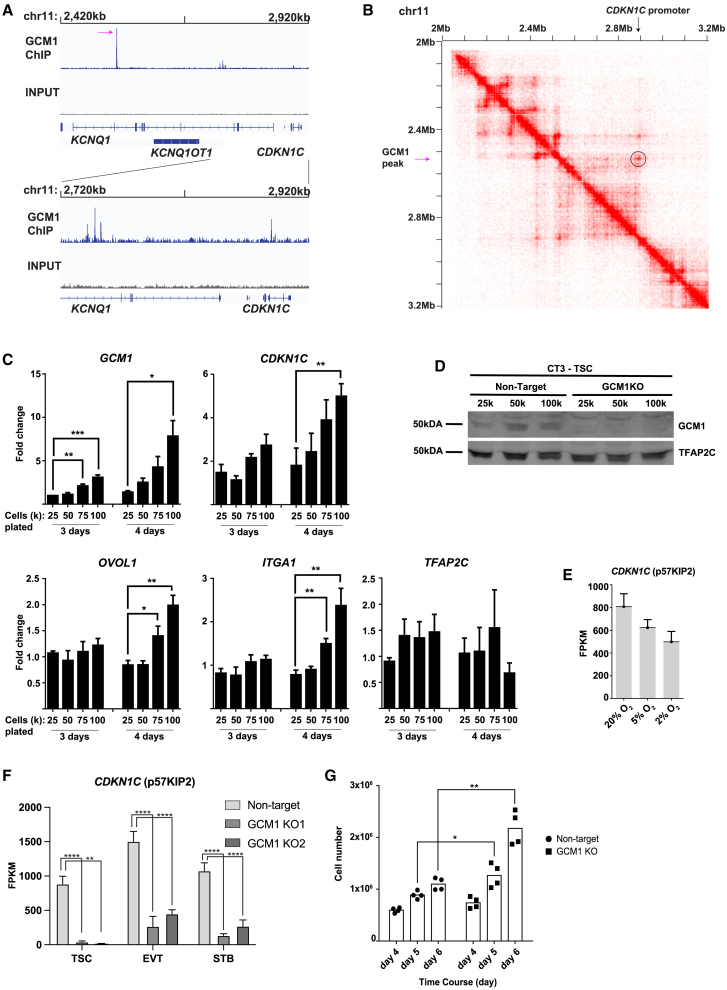
GCM1 positively regulates *CDKN1C* and contact inhibition (A) GCM1 enrichment over imprinted *KCNQ* locus. (B) Hi-C interaction data over the *GCM1* locus. Note physical association between GCM1-binding site and *CDKN1C* promoter. In (A) and (B), the highest GCM1 peak is indicated with a magenta arrow. (C) Expression of genes indicated in plating conditions (cell number and growth time) indicated. Note that plating at higher densities leads to higher expression of *GCM1* and *CDKN1C* (CT1 hTSCs, *n* = 4 replicates). Statistical significance was determined via a two-tailed t test. (D) GCM1 protein levels increase with higher confluence. (E) Expression of *CDKN1C* in oxygen concentration indicated. (F) Expression of *CDKN1C* in control and *GCM1*^−/−^ KO1 and KO2 hTSC and differentiated cells (significance marked by *p*_adj._ value from DESEQ2 analysis, see Table S3). (G) Cell number after plating 50k cells and allowing cells to grow for indicated number of days. Note a leveling off in non-targeting cells as cell lines reach confluence, but continued growth in *GCM1*^−/−^ hTSCs (CT3 hTSCs, *n* = 4 replicates, including *n* = 2 GCM1KO1 and *n* = 2 GCM1KO2). Statistical significance was determined via a two-tailed t-test.

One of the strongest GCM1 enrichment sites in the genome was found within the 11p15.5 imprinted locus (Smilinich et al., 1999), as were several smaller peaks (Figure 4A). This locus includes the transcripts *KCNQ1* and *KCNQ1OT1* and the protein CDKN1C (p57^KIP2^), which binds to cyclin/CDK and blocks cell division (Takahashi et al., 2019). Analysis of published Hi-C data (Varberg et al., 2023), which shows three-dimensional interaction of regions of chromatin, shows a high degree of interaction between the strong GCM1-binding site in *KCNQ1* and the promoter of *CDKN1C* (Figures 4A and 4B).

CDKN1C shows preferential expression from the maternal allele (Matsuoka et al., 1995). A pregnancy abnormality called a full hydatidiform mole arises from an androgenetic pregnancy in which all genetic material is of paternal origin, and thus placental cells in hydatidiform moles feature loss of *CDKN1C* as well as persistent trophoblastic outgrowth (Jun et al., 2003). hTSCs upregulate *CDKN1C* at high confluence, and *CDKN1C*^*−/-*^ hTSCs lose contact inhibition and continue growing after reaching confluence (Takahashi et al., 2019).

When we grew hTSCs to high confluence, we observed upregulation of *GCM1* and *CDKN1C* in tandem (Figures 4C and 4D), with more modest increases in other differentiation markers (Figure 4C). We also observed lower *CDKN1C* expression in hypoxia (Figure 4E), where levels of GCM1 are lower (Figures 1K and S1L), though this drop in *CDKN1C* does not reach statistical significance. Further consistent with direct regulation by GCM1, *CDKN1C* was dramatically downregulated in *GCM1*^−/−^ hTSCs and differentiated cells (Figure 4F). While control cells stopped dividing as confluence occurs, *GCM1*^−/−^ hTSCs continued to expand, similar to the reported *CDKN1C*^−/−^ phenotype (Figures 4G and S4) (Takahashi et al., 2019). These results collectively indicate that GCM1 acts upstream of *CDKN1C* in response to confluence and controls its expression.

## Discussion

Considering the positive effect of hypoxia on spongiotrophoblast differentiation in mice, how can we explain the generally inhibitory effect of low oxygen on EVT differentiation in humans? A critical difference between mice and humans, as illustrated in our work and others (Jeyarajah et al., 2022; Shimizu et al., 2023; Wang et al., 2022), is that GCM1 is essential for both EVT and STB formation in human but only STB formation in mouse. Hence, if hypoxia negatively regulates GCM1 in both mice and humans, this would be predicted to have an inhibitory effect on EVT differentiation in humans but not on spongiotrophoblast or trophoblast giant cell formation in mice. We also find, somewhat counterintuitively, that hypoxia has a positive effect on HLA-G expression even though it has an overall negative effect on EVT differentiation. It is worth noting here that hTSCs, while clearly bipotent, express some markers consistent with cell column CTB, indicating that they may be more EVT-like than typical villous CTBs (Cinkornpumin et al., 2020; Lee et al., 2018a; Shannon et al., 2024). Hence, the observation that hypoxia promotes proximal-column EVT transcriptional program (Wakeland et al., 2017) is not necessarily incompatible with a role in hTSC self-renewal.

With regard to pathology, preeclampsia is widely understood to feature inadequate remodeling of maternal arterioles and concomitant reduced blood and oxygen availability for the placenta (Zhou et al., 1997), and higher levels of HIF-1α and/or HIF-2α protein have been observed in preeclamptic placenta (Soares et al., 2017). Excess of undifferentiated CTBs, which our study predicts would result from hypoxia, has also been reported (Redline and Patterson, 1995).

TSC organoids derived from human placenta collected over several gestational stages have the consistent feature that STB differentiation occurs inside the organoid (Haider et al., 2018; Sheridan et al., 2021; Turco et al., 2018; Yang et al., 2022). This may reflect higher pressure inside the organoid, or lack of access to Epidermal growth factor (EGF), but this organization is inverted with respect to the bilayer formation of chorionic villus (Enders and Blankenship, 1999). Two groups have succeeded in finding conditions in which STBs form on the outside of the organoid, though the resulting organoids cannot be propagated (Hori et al., 2024; Yang et al., 2024). We reasoned that reduction of *GCM1* level, as reported in literature from the PI3K inhibitor treatment, could allow sustained culture of undifferentiated organoids. We did observe reduced, though not eliminated, spontaneous differentiation. Interestingly, the published mechanism by which LY294002 reduces GCM1 expression, via activation of GSK3β, which then phosphorylates and degrades GCM1 (Chiang et al., 2009), would not be expected to work in TSCM media conditions in which GSK3β is perpetually inhibited via treatment with CHIR99021. Hence, it is something of a mystery how LY294002 prevents spontaneous differentiation in the organoid model, and future research in this area could yield improved culture conditions.

There is extensive literature demonstrating a role for Wnt signaling in maintenance of CTBs and hTSCs, along with substantial evidence for a role for GCM1 in the suppression of Wnt signaling. Nuclear β-catenin, the output of the canonical Wnt pathway, is observed in villous CTBs but is lost upon subsequent differentiation (Haider et al., 2018). CHIR99021, a Wnt activator, is essential for hTSC maintenance, and its removal facilitates directed differentiation to downstream lineages (Okae et al., 2018). Likewise, removal of CHIR99021 from self-renewing TB-ORG conditions is sufficient to allow differentiation to EVT (Haider et al., 2018). Concordantly, knockdown of *GCM1* in hTSCs results in elevated levels of Wnt pathway signaling, leading to failed EVT differentiation (Jeyarajah et al., 2022). Our results are broadly consistent with these findings, and we observe Wnt pathway ontology terms upregulated in 2% O_2_ conditions (Figure 1I). Cell column CTBs show low expression of both the Wnt receptor *WLS* and *GCM1* (Figure S2L). We note that *GCM1*^−/−^ TB-ORG cultured without CHIR99021 to induce EVT differentiation expressed the cell column marker ITGB6, suggesting that this is the most differentiated state that can be attained by *GCM1*-deficient hTSCs.

In a CRISPR dropout screen of hTSCs, *GCM1* is a growth-restricting gene, whose deletion promotes cell growth (Dong et al., 2022). This is likely due to two mechanisms. As shown earlier, hTSCs, especially at high confluence, undergo some spontaneous differentiation, which is suppressed by loss of GCM1. Furthermore, *GCM1*^−/−^ hTSCs show greatly reduced *CDKN1C* expression, limiting contact inhibition. Indeed, *CDKN1C* is also a growth-restricting gene in CRISPR screens (Dong et al., 2022; Shimizu et al., 2023). Interestingly, despite its growth-restricting properties in culture, *GCM1* is not a known tumor suppressor in gestational choriocarcinoma (GC) (Fisher and Maher, 2021; Jung et al., 2020; Mello et al., 2017). In the case of hydatidiform-mole-derived GC, *CDKN1C* expression is already lost, but GC can also arise from non-molar pregnancy, and there is no evidence of GCM1 mutation in these diseases either. This may be because while loss of *GCM1* causes loss of contact inhibition and uncontrolled growth, it also precludes epithelial-mesenchymal transition (EMT) and invasiveness. At the same time, analysis of mutations and karyotypic abnormalities in GC remains limited, and there is almost nothing known about the mutational profile of the related placental cancers, placental site trophoblastic tumor, and epithelioid trophoblastic tumor (Hui et al., 2004; Oliver et al., 2021; Xu et al., 2009). New roles for GCM1 in placental development and placental cancer may have yet to be discovered.

## Methods

### Cell culture maintenance and differentiation of hTSC

CT1, CT3, BT1, and BT2 hTSC lines were generously provided from Dr. Arima’s lab in Japan with STR authentication. CT1 and CT3 were derived from first trimester placenta, and BT1 and BT2 hTSC were derived from blastocysts (Okae et al., 2018). Cell culture, including thawing, freezing and differentiating cells was performed according to published protocol (Okae et al., 2018). A detailed description of the maintenance and differentiation produces is included in the supplemental methods.

All cells were free of visible contamination and routinely tested negative for mycoplasma.

### Cell culture—Standard maintenance and EVT differentiation of TB-ORGs

TB-ORGs were generated and cultured according to a recent publication (Haider et al., 2022). A detailed description is available in the supplemental methods .

### TB-ORG and EVT differentiation (modified protocol without Matrigel)

3D TSCs (TB-ORG) of control and *GCM1*^−/−^ cells were cultured based on a modification from the Okae et al. (2018) and Haider et al. (2022) publications. Our modified trophoblast organoid media (mTOM) contains DMEM F-12 (Gibco), 10 mM HEPES (Gibco), 1× ITS-X (Gibco), 2 mM GlutaMax (Gibco), 1× Penicillin/Streptomycin (Gibco), 0.2% ESC-FBS (Gibco), 50 ng/mL rhEGF (Invitrogen), 3 μM CHIR99021 (Cayman Chemical), 2 μM A8301 (Cayman Chemical), and 5 μM Y27632 (Cayman Chemical). Instead of Matrigel embedding, micro-V-shaped wells (AggreWell 400, STEMCELL Technologies) were used to generate organoids. ∼50,000 cells were resuspended in 1 mL mTOM per 24 well and centrifuged in a plate spinner for 5 min at 1,000 rpm to collect cells to the bottom at ∼100 cells per V-shaped well. After 24 h, 500 μL of media is gently removed and either mTOM is replaced or mTOM without CHIR99021 (mTOM-C) for CTB-CCC/precursor to EVT formation was replaced every other day over a ∼10-day period. For LY294002 treatment, treatment up to 14 days may be required to physically observe cavity formation by microscopy.

### Generation of CRISPR knockout lines by nucleofection

CRISPR guides were designed using IDT Custom Alt-R CRISPR-Cas9 guide RNA program. sgRNA guides targeting a small region of nucleotide just after the ATG start site in exon 2 and introns 2–3 (sgRNA1: UCU UCA GAA UCA AAG UCG UC and sgRNA2: ACU AUU AAC AUG CGG AGA CC). Additional deletion of exon 3 was designed (sgRNA3: GAG CGC UGC UCA GAU AGC GA and sgRNA4: AGA CCU AAG AGC AAU CAG UG). Cas9-sgRNA ribonucleoprotein complexes were generated from sgRNA and Cas9 protein (Synthego). In brief, 75 pmol of each individual sgRNA is complexed with 10 pmol of Cas9 protein in Cas9 annealing buffer (NEB) for 10 min. In the meantime, TSCs are dissociated with 30% TrypLE and reconstituted in PBS to a concentration of 1 × 10^5^ cell/μL. Pre-complexed ribonucleoproteins sgRNA1 and sgRNA2 are combined together with 10 μL of cell suspension and 20 μL of P3 solution (Lonza) and transferred to a cuvette to be nucleofected using an Amaxa 4D nucleofector (Lonza) with pulse code CA137. Immediately after, 150 μL of TSC media was added to the cuvette to neutralize the reaction, and cells were transferred to a freshly prepared 10 cm plate coated with Lam-511 and TSCM for generating single clones. 2 mg/mL Collagenase V solution was used to dissociate colonies in order to pick single clones. Deletions were confirmed by genotyping (GCM1KO1: forward, TTGTATGAGGACTTGTGCATAACAA and reverse, GCCATTGGTTACAGATGACAAC; GCM1KO2: forward, ATGGAACTCACAGGGGCTAT and reverse, TAACAGGAGCCTTCAGTCCA).

### hCG ELISA

Human chorionic gonadatropin (hCG) secretion was measured using an hCG AccuBind ELISA (Monobind) according to manufacturer’s instructions.

### Confluence experiment

CT1 and CT3 hTSCs were plated in 24-well plates at 4 different densities (25, 50, 75, and 100K cells), in duplicates, and incubated for 48 or 72 h. Cells were collected using TrypLE 30% at 37°C for 10 min followed by addition of trypsin inhibitor. Single cells obtained were centrifuged to obtain a pellet, washed with PBS, and flash frozen and stored at −80°C.

### RNA isolation and qPCR

A detailed description of RNA isolation and qPCR, including a table of primers used, is included in the supplemental methods section.

### Next-generation sequencing libraries

ChIP was performed as previously described (Jeyarajah et al., 2022). ATAC-seq libraries were generated using a commercially available kit Active Motif (#53150, Carlsbad, CA). RNA-seq libraries were generated using an NEBNext Ultra RNA Library Prep Kit.

See supplemental methods for detailed descriptions of library generation.

### Sequencing analysis

For RNA-seq and ChIP/ATAC-seq analysis, Genpipes 4.3.2 (https://bitbucket.org/mugqic/genpipes) provided the pipeline for basic sequencing processing. For RNA-seq, sequencing quality and adaptor removal were trimmed with trimmomatic (v.0.36). Trimmed fastq file alignment was performed with STAR aligner (v.2.7.8a) using hg38/GRCh38 (ensembl v.104). Picard (v.2.9.0) was then used to merge, mark duplicates, identify unique read, and sort .bam files proceeding alignment. Read counts were collected using HTseq-count and StringTie (v.1.3.5). Differentially expressed gene comparison was performed with DESeq2 package on RStudio (R v.4.3.1). Correlation matrix, hierarchal gene cluster analysis, and PCA were generated in R. Gene pathway analysis was performed using ConsensusPathwayDB and EnrichR. For ChIP-seq and ATAC-seq, in brief, raw fastq files were trimmed using trimmomatic (v.0.36). Then, qualified fastq reads were mapped with BWA (v.0.7.17) with post processing with sambamda (v.0.8.1) to merge replicates, mark and filter duplicates, and remove blacklist regions. Peak calling and differential bind were performed with MACS2 (v.2.2.7.1). Gene annotations and motif analysis were performed with Homer (v.4.11). Bam to bigwig bamCoverage from deepTools (v.3.5.1) was used to generate the tracks for viewing on IGV (v.2.9.4).

### Ethics

All research was approved by the McGill Faculty of Medicine and Health Sciences Research Ethics Board (McGill IRB).

## Resource availability

### Lead contact

Requests for further information or cell lines will be fulfilled by the lead contact, William A. Pastor (william.pastor@mcgill.ca).

### Materials availability

Cell lines generated in the course of this study will be made available upon reasonable requests.

### Data and code availability

Sequencing data were deposited to the Gene Expression Omnibus (GEO) repository with the following accession numbers: RNA-seq (GSE276594 and GSE276595), ATAC-seq (GSE276588), and ChIP-seq (GSE276590). Passage number is indicated in GEO submission.

## Acknowledgments

We thank the Rosalind & Morris Goodman Cancer Institute Flow Cytometry core, the SickKids Center for Applied Genomics facility, the La Jolla Institute for Allergy and Immunology Sequencing Core, and the Canada Michael Smith Genome Sciences Center at BC Cancer for their dedicated service. We thank Dr. Brian Cox (University of Toronto) for sharing code to perform single-cell RNA-seq analysis. This work was funded by the New Frontiers in Research Fund (NFRF) grant NFRFE-2018-00883 and the 10.13039/501100000024Canadian Institutes of Health Research (CIHR) project grant PJT-166169 to W.A.P., the 10.13039/100000002NIH grants HD101319, HD062546, and HD103161 to S.P., the NSERC Discovery Grant RGPIN-2016-05053 and CIHR project grant PJT-180483 to S.J.R. and the 10.13039/501100002428Austrian Science Fund P34588-B and P36159-B to S.H. W.A.P. was supported by an FRQS Chercheurs-boursier. J.K.C. was supported by a Fonds de recherche Santé Québec graduate fellowship and studentships from the McGill University Faculty of Medicine.

## Author contributions

J.K.C., S.Y.K., A.-M.P., T.M., J.S., J.G., J.Z., P.D., and M.J.J. conducted the experiments. J.K.C. and D.S. conducted the bioinformatic analysis. S.J.R., S.P., S.H., and W.A.P. supervised the experiments and analysis.

## Declaration of interests

The authors declare no competing interests.

## Supplemental information

Document S1. Figures S1–S4 and supplemental methods

Table S1. Gene expression associated with different oxygen concentrations, including FPKM of all samples, differentially expressed genes for 2% and 5% O_2_ as compared with 20% O_2_, and gene cluster analysis to identify genes associated with O_2_ level

Table S2. Expression of genes specific to hTSC, EVT, and STB in hTSCs grown at 20%, 5%, and 2% O_2_

Table S3. Gene expression associated with WT and GCM1 KO lines, including FPKM, expression of hTSC, STB, and EVT-specific genes, and genes identified as differentially expressed between WT and GCM1 KO

Table S4. GCM1 ChIP-seq peaks as well as ATAC-seq peaks specific to cell types indicated

Document S2. Article plus supplemental information

## References

[bib1] Alsat E., Wyplosz P., Malassiné A., Guibourdenche J., Porquet D., Nessmann C., Evain-Brion D. (1996). Hypoxia impairs cell fusion and differentiation process in human cytotrophoblast, in vitro. J. Cell. Physiol..

[bib2] Arutyunyan A., Roberts K., Troulé K., Wong F.C.K., Sheridan M.A., Kats I., Garcia-Alonso L., Velten B., Hoo R., Ruiz-Morales E.R. (2023). Spatial multiomics map of trophoblast development in early pregnancy. Nature.

[bib3] Chang C.W., Wakeland A.K., Parast M.M. (2018). Trophoblast lineage specification, differentiation and their regulation by oxygen tension. J. Endocrinol..

[bib4] Chazaud C., Yamanaka Y. (2016). Lineage specification in the mouse preimplantation embryo. Development.

[bib5] Chen Y., Meng Y., Yu Y., Li W., Shen Y., Li S., Chang Y., Sun W. (2022). LMO2 plays differential roles in trophoblast subtypes and is associated with preeclampsia. Biochem. Biophys. Res. Commun..

[bib6] Chiang M.H., Liang F.Y., Chen C.P., Chang C.W., Cheong M.L., Wang L.J., Liang C.Y., Lin F.Y., Chou C.C., Chen H. (2009). Mechanism of hypoxia-induced GCM1 degradation: implications for the pathogenesis of preeclampsia. J. Biol. Chem..

[bib7] Chiu Y.H., Chen H. (2016). GATA3 inhibits GCM1 activity and trophoblast cell invasion. Sci. Rep..

[bib8] Cinkornpumin J.K., Kwon S.Y., Guo Y., Hossain I., Sirois J., Russett C.S., Tseng H.W., Okae H., Arima T., Duchaine T.F. (2020). Naive Human Embryonic Stem Cells Can Give Rise to Cells with a Trophoblast-like Transcriptome and Methylome. Stem Cell Rep..

[bib9] Cowden Dahl K.D., Fryer B.H., Mack F.A., Compernolle V., Maltepe E., Adelman D.M., Carmeliet P., Simon M.C. (2005). Hypoxia-inducible factors 1alpha and 2alpha regulate trophoblast differentiation. Mol. Cell Biol..

[bib10] Dong C., Fu S., Karvas R.M., Chew B., Fischer L.A., Xing X., Harrison J.K., Popli P., Kommagani R., Wang T. (2022). A genome-wide CRISPR-Cas9 knockout screen identifies essential and growth-restricting genes in human trophoblast stem cells. Nat. Commun..

[bib11] Enders A.C., Blankenship T.N. (1999). Comparative placental structure. Adv. Drug Deliv. Rev..

[bib12] Fisher R.A., Maher G.J. (2021). Genetics of gestational trophoblastic disease. Best Pract. Res. Clin. Obstet. Gynaecol..

[bib13] Genbacev O., Joslin R., Damsky C.H., Polliotti B.M., Fisher S.J. (1996). Hypoxia alters early gestation human cytotrophoblast differentiation/invasion in vitro and models the placental defects that occur in preeclampsia. J. Clin. Investig..

[bib14] Genbacev O., Zhou Y., Ludlow J.W., Fisher S.J. (1997). Regulation of human placental development by oxygen tension. Science.

[bib15] Gnarra J.R., Ward J.M., Porter F.D., Wagner J.R., Devor D.E., Grinberg A., Emmert-Buck M.R., Westphal H., Klausner R.D., Linehan W.M. (1997). Defective placental vasculogenesis causes embryonic lethality in VHL-deficient mice. Proc. Natl. Acad. Sci. USA.

[bib16] Guo Y., Xue Z., Yuan R., Li J.J., Pastor W.A., Liu W. (2021). RAD: a web application to identify region associated differentially expressed genes. Bioinformatics.

[bib17] Haider S., Lackner A.I., Dietrich B., Kunihs V., Haslinger P., Meinhardt G., Maxian T., Saleh L., Fiala C., Pollheimer J. (2022). Transforming growth factor-beta signaling governs the differentiation program of extravillous trophoblasts in the developing human placenta. Proc. Natl. Acad. Sci. USA.

[bib18] Haider S., Meinhardt G., Saleh L., Kunihs V., Gamperl M., Kaindl U., Ellinger A., Burkard T.R., Fiala C., Pollheimer J. (2018). Self-Renewing Trophoblast Organoids Recapitulate the Developmental Program of the Early Human Placenta. Stem Cell Rep..

[bib19] Hori T., Okae H., Shibata S., Kobayashi N., Kobayashi E.H., Oike A., Sekiya A., Arima T., Kaji H. (2024). Trophoblast stem cell-based organoid models of the human placental barrier. Nat. Commun..

[bib20] Hui P., Riba A., Pejovic T., Johnson T., Baergen R.N., Ward D. (2004). Comparative genomic hybridization study of placental site trophoblastic tumour: a report of four cases. Mod. Pathol..

[bib21] Jaremek A., Shaha S., Jeyarajah M.J., Jaju Bhattad G., Chowdhury D., Riddell M., Renaud S.J. (2023). Genome-Wide Analysis of Hypoxia-Inducible Factor Binding Reveals Targets Implicated in Impaired Human Placental Syncytiotrophoblast Formation under Low Oxygen. Am. J. Pathol..

[bib22] Jauniaux E., Watson A., Burton G. (2001). Evaluation of respiratory gases and acid-base gradients in human fetal fluids and uteroplacental tissue between 7 and 16 weeks' gestation. Am. J. Obstet. Gynecol..

[bib23] Jeyarajah M.J., Jaju Bhattad G., Kelly R.D., Baines K.J., Jaremek A., Yang F.H.P., Okae H., Arima T., Dumeaux V., Renaud S.J. (2022). The multifaceted role of GCM1 during trophoblast differentiation in the human placenta. Proc. Natl. Acad. Sci. USA.

[bib24] Jun S.Y., Ro J.Y., Kim K.R. (2003). p57kip2 is useful in the classification and differential diagnosis of complete and partial hydatidiform moles. Histopathology.

[bib25] Jung S.H., Choi Y.J., Kim M.S., Park H.C., Han M.R., Hur S.Y., Lee A.W., Shin O.R., Kim J., Lee S.H. (2020). Distinct genomic profiles of gestational choriocarcinoma, a unique cancer of pregnant tissues. Exp. Mol. Med..

[bib26] Kozak K.R., Abbott B., Hankinson O. (1997). ARNT-deficient mice and placental differentiation. Dev. Biol..

[bib27] Lee C.Q.E., Turco M.Y., Gardner L., Simons B.D., Hemberger M., Moffett A. (2018). Integrin alpha2 marks a niche of trophoblast progenitor cells in first trimester human placenta. Development.

[bib28] Lee C.Q.E., Turco M.Y., Gardner L., Simons B.D., Hemberger M., Moffett A. (2018). Integrin α2 marks a niche of trophoblast progenitor cells in first trimester human placenta. Development.

[bib29] Li S., Roberson M.S. (2017). Dlx3 and GCM-1 functionally coordinate the regulation of placental growth factor in human trophoblast-derived cells. J. Cell. Physiol..

[bib30] Majmundar A.J., Wong W.J., Simon M.C. (2010). Hypoxia-inducible factors and the response to hypoxic stress. Mol. Cell.

[bib31] Matsuoka S., Edwards M.C., Bai C., Parker S., Zhang P., Baldini A., Harper J.W., Elledge S.J. (1995). p57KIP2, a structurally distinct member of the p21CIP1 Cdk inhibitor family, is a candidate tumor suppressor gene. Genes Dev..

[bib32] Mello J.B.H.d., Ramos Cirilo P.D., Michelin O.C., Custódio Domingues M.A., Cunha Rudge M.V., Rogatto S.R., Maestá I. (2017). Genomic profile in gestational and non-gestational choriocarcinomas. Placenta.

[bib33] Natsuizaka M., Naganuma S., Kagawa S., Ohashi S., Ahmadi A., Subramanian H., Chang S., Nakagawa K.J., Ji X., Liebhaber S.A. (2012). Hypoxia induces IGFBP3 in esophageal squamous cancer cells through HIF-1alpha-mediated mRNA transcription and continuous protein synthesis. FASEB J..

[bib34] Nelson D.M., Johnson R.D., Smith S.D., Anteby E.Y., Sadovsky Y. (1999). Hypoxia limits differentiation and up-regulates expression and activity of prostaglandin H synthase 2 in cultured trophoblast from term human placenta. Am. J. Obstet. Gynecol..

[bib35] Okae H., Toh H., Sato T., Hiura H., Takahashi S., Shirane K., Kabayama Y., Suyama M., Sasaki H., Arima T. (2018). Derivation of Human Trophoblast Stem Cells. Cell Stem Cell.

[bib36] Oliver G.R., Marcano-Bonilla S., Quist J., Tolosa E.J., Iguchi E., Swanson A.A., Hoppman N.L., Schwab T., Sigafoos A., Prodduturi N. (2021). LPCAT1-TERT fusions are uniquely recurrent in epithelioid trophoblastic tumors and positively regulate cell growth. PLoS One.

[bib37] Redline R.W., Patterson P. (1995). Pre-eclampsia is associated with an excess of proliferative immature intermediate trophoblast. Hum. Pathol..

[bib38] Rodesch F., Simon P., Donner C., Jauniaux E. (1992). Oxygen measurements in endometrial and trophoblastic tissues during early pregnancy. Obstet. Gynecol..

[bib39] Sato Y. (2020). Endovascular trophoblast and spiral artery remodeling. Mol. Cell. Endocrinol..

[bib40] Shannon M.J., McNeill G.L., Koksal B., Baltayeva J., Wächter J., Castellana B., Peñaherrera M.S., Robinson W.P., Leung P.C.K., Beristain A.G. (2024). Single-cell assessment of primary and stem cell-derived human trophoblast organoids as placenta-modeling platforms. Dev. Cell.

[bib41] Sheridan M.A., Zhao X., Fernando R.C., Gardner L., Perez-Garcia V., Li Q., Marsh S.G.E., Hamilton R., Moffett A., Turco M.Y. (2021). Characterization of primary models of human trophoblast. Development.

[bib42] Sherman B.T., Hao M., Qiu J., Jiao X., Baseler M.W., Lane H.C., Imamichi T., Chang W. (2022). DAVID: a web server for functional enrichment analysis and functional annotation of gene lists (2021 update). Nucleic Acids Res..

[bib43] Shimizu T., Oike A., Kobayashi E.H., Sekiya A., Kobayashi N., Shibata S., Hamada H., Saito M., Yaegashi N., Suyama M. (2023). CRISPR screening in human trophoblast stem cells reveals both shared and distinct aspects of human and mouse placental development. Proc. Natl. Acad. Sci. USA.

[bib44] Smilinich N.J., Day C.D., Fitzpatrick G.V., Caldwell G.M., Lossie A.C., Cooper P.R., Smallwood A.C., Joyce J.A., Schofield P.N., Reik W. (1999). A maternally methylated CpG island in KvLQT1 is associated with an antisense paternal transcript and loss of imprinting in Beckwith-Wiedemann syndrome. Proc. Natl. Acad. Sci. USA.

[bib45] Soares M.J., Iqbal K., Kozai K. (2017). Hypoxia and Placental Development. Birth Defects Res..

[bib46] Takahashi S., Okae H., Kobayashi N., Kitamura A., Kumada K., Yaegashi N., Arima T. (2019). Loss of p57(KIP2) expression confers resistance to contact inhibition in human androgenetic trophoblast stem cells. Proc. Natl. Acad. Sci. USA.

[bib47] Takeda K., Ho V.C., Takeda H., Duan L.J., Nagy A., Fong G.H. (2006). Placental but not heart defects are associated with elevated hypoxia-inducible factor alpha levels in mice lacking prolyl hydroxylase domain protein 2. Mol. Cell Biol..

[bib48] Turco M.Y., Gardner L., Kay R.G., Hamilton R.S., Prater M., Hollinshead M.S., McWhinnie A., Esposito L., Fernando R., Skelton H. (2018). Trophoblast organoids as a model for maternal-fetal interactions during human placentation. Nature.

[bib49] Varberg K.M., Dominguez E.M., Koseva B., Varberg J.M., McNally R.P., Moreno-Irusta A., Wesley E.R., Iqbal K., Cheung W.A., Schwendinger-Schreck C. (2023). Extravillous trophoblast cell lineage development is associated with active remodeling of the chromatin landscape. Nat. Commun..

[bib50] Wakeland A.K., Soncin F., Moretto-Zita M., Chang C.W., Horii M., Pizzo D., Nelson K.K., Laurent L.C., Parast M.M. (2017). Hypoxia Directs Human Extravillous Trophoblast Differentiation in a Hypoxia-Inducible Factor-Dependent Manner. Am. J. Pathol..

[bib51] Wang L.J., Chen C.P., Lee Y.S., Ng P.S., Chang G.D., Pao Y.H., Lo H.F., Peng C.H., Cheong M.L., Chen H. (2022). Functional antagonism between DeltaNp63alpha and GCM1 regulates human trophoblast stemness and differentiation. Nat. Commun..

[bib52] Xu M.L., Yang B., Carcangiu M.L., Hui P. (2009). Epithelioid trophoblastic tumor: comparative genomic hybridization and diagnostic DNA genotyping. Mod. Pathol..

[bib53] Yang L., Liang P., Yang H., Coyne C.B. (2024). Trophoblast organoids with physiological polarity model placental structure and function. J. Cell Sci..

[bib54] Yang L., Semmes E.C., Ovies C., Megli C., Permar S., Gilner J.B., Coyne C.B. (2022). Innate immune signaling in trophoblast and decidua organoids defines differential antiviral defenses at the maternal-fetal interface. Elife.

[bib55] Zhou Y., Damsky C.H., Fisher S.J. (1997). Preeclampsia is associated with failure of human cytotrophoblasts to mimic a vascular adhesion phenotype. One cause of defective endovascular invasion in this syndrome?. J. Clin. Investig..

