# Supplementary material for: Hypoxia and loss of GCM1 expression prevent differentiation and contact inhibition in human trophoblast stem cells
Source: Stem Cell Reports. 2025 Apr 24;20(5):102481. doi: 10.1016/j.stemcr.2025.102481 (PMC12143156; doi:10.1016/j.stemcr.2025.102481)
Supplement: Document S1. Figures S1–S4 and supplemental methods [file mmc1.pdf]

Stem Cell Reports, Volume *20*

Supplemental Information

Hypoxia and loss of *GCM1* expression prevent differentiation and contact inhibition in human trophoblast stem cells

Jessica K. Cinkornpumin, Sin Young Kwon, Anna-Maria Prandstetter, Theresa Maxian, Jacinthe Sirois, James Goldberg, Joy Zhang, Deepak Saini, Purbasa Dasgupta, Mariyan J. Jeyarajah, Stephen J. Renaud, Soumen Paul, Sandra Haider, and William A. Pastor

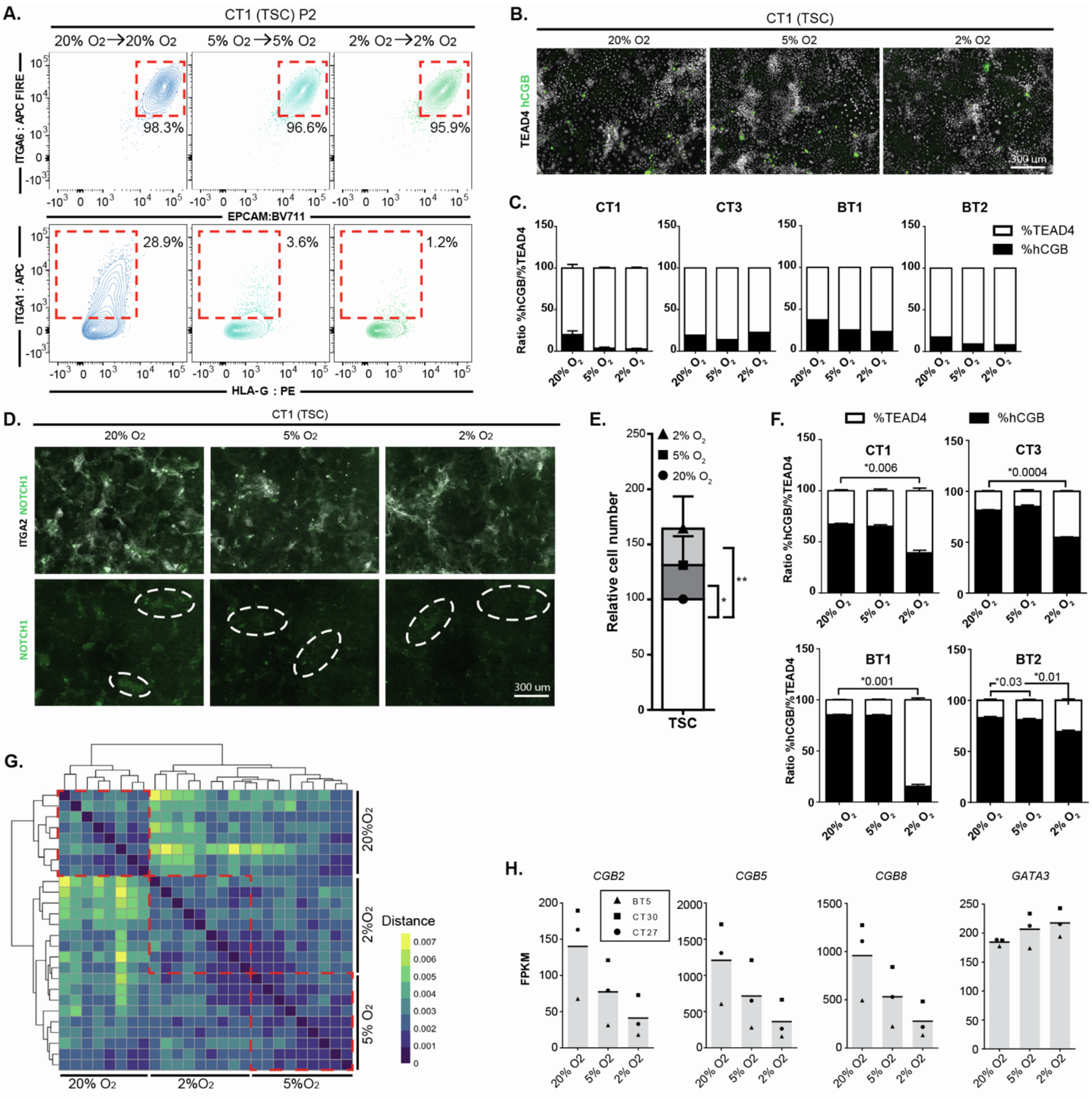

I. Enriched transcription factor target sets Cluster1

351 genes (13.8%) from the input list are present in at least one TF target set.
The total number of genes present in at least one TF target set and identifiable by 'ensembl' IDs is **2373**.

transcription factor	set size	candidates contained	p-value	q-value
HIF1A	82	30 (36.6%)	5.51e-07	0.000103
PTTG1	15	9 (60.0%)	6.74e-05	0.0063
MYCN	45	16 (35.6%)	0.000398	0.0248
MYC	99	25 (25.5%)	0.00316	0.129
VHL	20	8 (40.0%)	0.00523	0.129
HDGF	6	4 (66.7%)	0.00552	0.129
ARNTL	6	4 (66.7%)	0.00552	0.129
HIVEP2	6	4 (66.7%)	0.00552	0.129
CLOCK	13	6 (46.2%)	0.00686	0.143
TFDP1	10	5 (50.0%)	0.00914	0.171

J. Enriched transcription factor target sets Cluster2

338 genes (11.5%) from the input list are present in at least one TF target set.
The total number of genes present in at least one TF target set and identifiable by 'ensembl' IDs is **2373**.

transcription factor	set size	candidates contained	p-value	q-value
GCM1	4	4 (100.0%)	0.000405	0.0595
ATF4	35	13 (37.1%)	0.000601	0.0595
KLF6	20	8 (40.0%)	0.00411	0.217
HIC1	9	5 (55.6%)	0.00439	0.217
DDIT3	18	7 (38.9%)	0.00866	0.269
PPARG	66	17 (25.8%)	0.00878	0.269

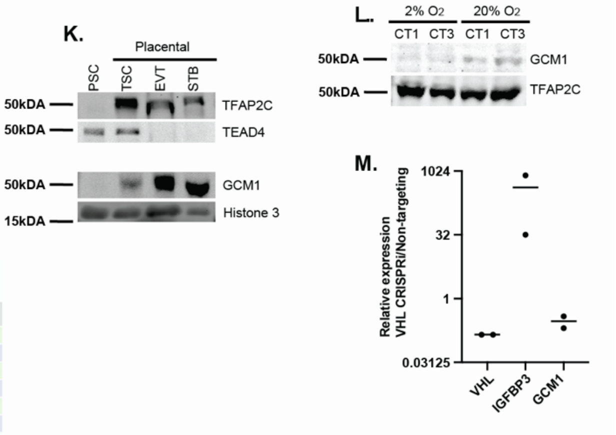

Figure S1 (related to Figure 1). Reduced and impaired hTSC differentiation in hypoxic conditions. A. Trophoblast stem cells shown in Figure. 1A were passaged and cultured for additional 72hrs in varying levels of oxygen (20%, 5%, 2% O_2). Continued low oxygen tension causes further reduction of ITGA1$^+$ cell population. **B.** Spontaneous differentiation of hTSC to STB in as indicated by loss of TEAD4 and gain of hCGB. Note trend toward higher hCGB staining at 20% O_2. **C.** Quantification of spontaneous hCGB expression across multiple cell lines at oxygen concentration indicated (4 cell lines, n=4 wells each cell line per condition). **D.** hTSC in the varying oxygen conditions were grown to over maximum confluency. At regions where overgrowth causes increase cell-to-cell contact and cell pile-up, spontaneous nuclear NOTCH1 signal is observed. In the low oxygen cultures, NOTCH1 expression is not detected. **E.** Relative numbers of TEAD4+ cells per unit area (4 cell lines, n=3 for each cell line over 3 different passages). **F.** Quantification of hCGB expression upon directed differentiation to STB in multiple cell lines at oxygen concentration indicated (4 cell lines, n=4 wells for each cell line per condition). **G.** Correlation matrix showing sample clustering of RNA-seq data from hTSCs in culture conditions indicated. **H.** Bar graphs showing FPKM of specific genes of interest (for G-H, 3 cell lines; BT2, CT1, CT3; n=3 replicates for each line in each condition over 3 passages, except BT2 at 20% O_2 n=2). **I., J.** ConsensusPathwayDB analysis of Cluster 1 and Cluster 2 from Figure 1H, identifying TF targets with significance from each cluster. **K.** Western blot comparing pluripotent stem cell (PSC) that don't express placental markers, with hTSC and differentiated EVT and STB to highlight specific expression patterns. **L.** Western blot for GCM1 in 2 cell lines grown in 2% and 20% O_2. TFAP2C is the loading control. **M.** Ratio of expression for genes indicated in hTSCs subjected to CRISPRi-targeted degradation of VHL relative to control hTSC. Note reduced expression of GCM1, while known hypoxia target IGFBP3 is dramatically upregulated.

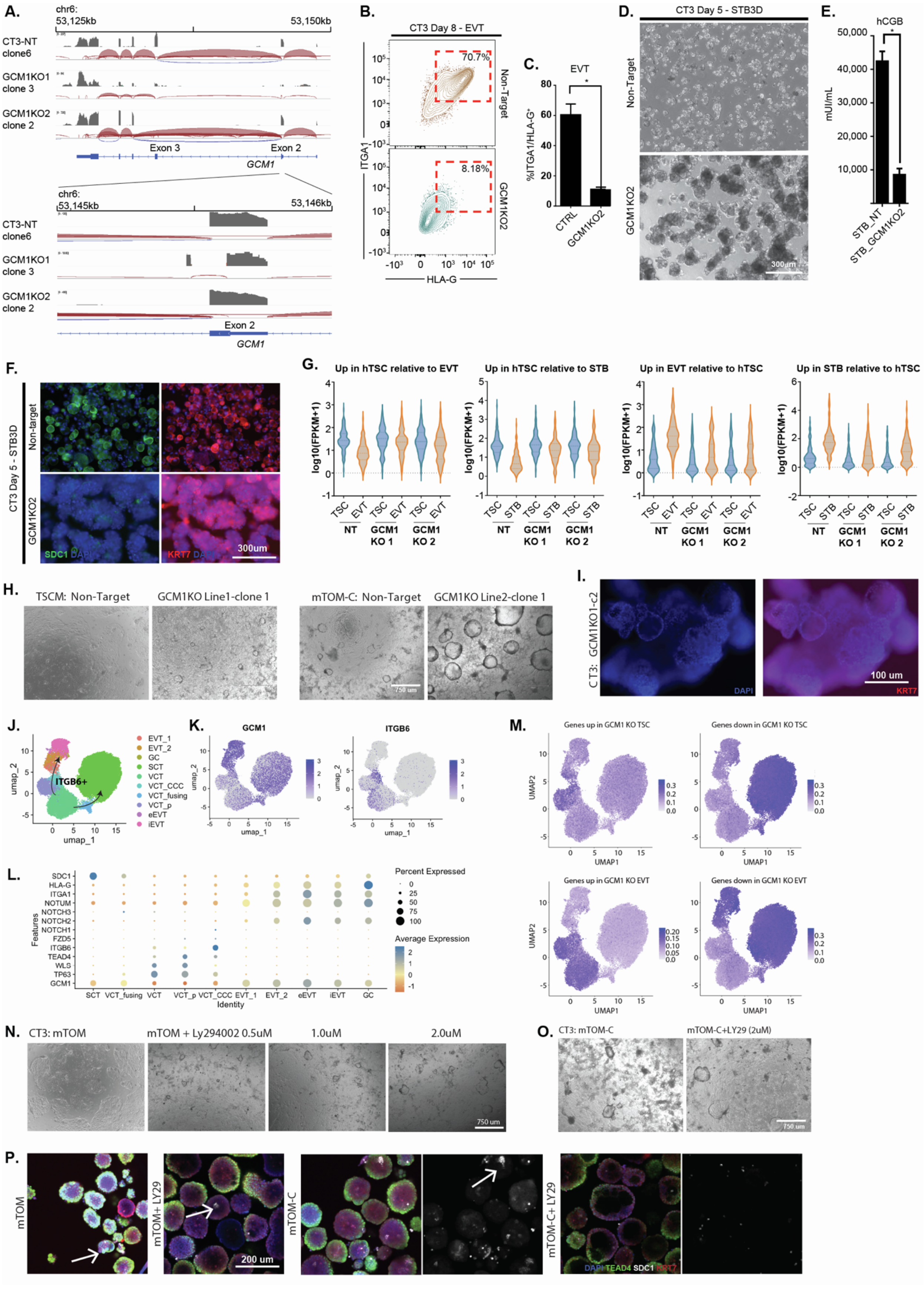
A.
chr6:
53,125kb
53,150kb
CT3-NT
clone6
GCM1KO1
clone 3
GCM1KO2
clone 2
Exon 3
Exon 2
GCM1
chr6:
53,145kb
53,146kb
CT3-NT
clone6
GCM1KO1
clone 3
GCM1KO2
clone 2
Exon 2
GCM1
B.
CT3 Day 8 - EVT
70.7%
Non-Target
8.18%
GCM1KO2
ITGA1
HLA-G
C.
EVT
%ITGA1/HLA-G+
CTRL
GCM1KO2
D.
CT3 Day 5 - STB3D
Non-Target
GCM1KO2
300um
E.
hCGB
mUI/mL
STB_NT
STB_GCM1KO2
F.
CT3 Day 5 - STB3D
Non-target
GCM1KO2
SDC1 DAPI
KRT7 DAPI
300um
G.
Up in hTSC relative to EVT
Up in hTSC relative to STB
Up in EVT relative to hTSC
Up in STB relative to hTSC
log10(FPKM+1)
TSC EVT TSC EVT TSC EVT
TSC STB TSC STB TSC STB
NT
GCM1 KO 1
GCM1 KO 2
H.
TSCM: Non-Target
GCM1KO Line1-clone 1
mTOM-C: Non-Target
GCM1KO Line2-clone 1
750 um
I.
CT3: GCM1KO1-c2
DAPI
KRT7
100 um
J.
ITGB6+
EVT_1
EVT_2
GC
SCT
VCT
VCT_CCC
VCT_fusing
VCT_p
eEVT
iEVT
umap_1
umap_2
K.
GCM1
ITGB6
M.
Genes up in GCM1 KO TSC
Genes down in GCM1 KO TSC
Genes up in GCM1 KO EVT
Genes down in GCM1 KO EVT
UMAP1
UMAP2
L.
SDC1
HLA-G
ITGA1
NOTUM
NOTCH3
NOTCH2
NOTCH1
FZD5
ITGB6
TEAD4
WLS
TP63
GCM1
Features
SCT VCT_fusing VCT VCT_p VCT_CCC EVT_1 EVT_2 eEVT iEVT GC
Identity
Percent Expressed
Average Expression
N.
CT3: mTOM
mTOM + Ly294002 0.5uM
1.0uM
2.0uM
750 um
O.
CT3: mTOM-C
mTOM-C+LY29 (2uM)
750 um
P.
mTOM
mTOM+ LY29
mTOM-C
mTOM-C+ LY29
200 um
DAPI TEAD4 SDC1 KRT7

Figure S2 (related to Figure 2). Impaired differentiation upon genetic or chemical reduction in GCM1 level. A. Sashimi plot across the genomic region of *GCM1*. Representative clones were chosen. Normal splicing is observed from non-target line. *GCM1*$^{-/-}$ KO1 (from right to left) show a deletion at the distal tip of exon 2 but an alternative slice site forming just after. *GCM1*$^{-/-}$ KO2 shows the complete skipping of exon 3. **B.** Flow cytometric analysis from EVT differentiation of *GCM1*$^{-/-}$ KO2 and NT hTSC. NT cells differentiation produce ITGA1^{hi}/HLA-G^{hi} cells whereas *GCM1*$^{-/-}$ TSC do not. (representative flow related to C). **C.** Bar graph showing formation of ITGA1^{hi}/HLA-G^{hi} EVTs from control and *GCM1*$^{-/-}$ KO2 hTSC (n=3 clonal replicates for NT and KO). **D.** STB3D formation of NT and *GCM1*$^{-/-}$ KO2 hTSC. Control hTSCs form a fluid-filled syncytium while *GCM1*$^{-/-}$ form a cluster of cells (representative image related to E). **E.** hCGB ELISA was perform using supernatant from *GCM1*$^{-/-}$ and control hTSC (n=3 clonal replicates for NT and KO). **F.** Control and *GCM1*$^{-/-}$ KO2 STB3D stained for the STB-marker SDC1 and the pan-placental marker CKT7. Note absence of SDC1 in *GCM1*$^{-/-}$ (representative image related to E). **G.** Violin plot showing expression of genes specific to hTSC, EVT or STB in cell types indicated. Differentiated *GCM1*$^{-/-}$ cells fail to express differentiation markers and retain expression of TSC markers instead (data analyze from Fig. 2G). **H.** *GCM1*$^{-/-}$-hTSC were grown in mTOM with and without CHIR99021. Dome-like projections appeared in regions of high cell density (representative image line CT3, n=7 each for NT and KO line CT1 and CT3). **I.** Immunofluorescent staining of *GCM1-/-* trophoblast organoids. **J.-L** Reanalysis of Arut. *et al.* 2022 single RNA-seq profiling the several subtypes found in early villus of the placenta. **J.** Cell types in placenta, with path of differentiation indicated. (GC=Giant Cell, VCT = villous CTB, VCT_p=proliferating CTB, VCT_CCC = cell column CTB eEVT=endovascular EVT, iEVT= interstitial EVT). **K.** Expression of GCM1 and ITGB6 in cells shown in (**J**). **L.** Expression of genes indicated in cell types indicated. Note that ITGB6 is associated with cell column cytotrophoblast. **M.** Average expression of gene set indicated ($\log_2$ FC>0.5, $p_{adj} < 0.05$) over cells shown in (**J**). **N.** hTSC cultured in mTOM media with varying concentrations of LY294002. **O.** hTSC cultured in mTOM-C with or without LY294002 2μM. **P.** day14 TB-ORG grown in mTOM or mTOM-C with or without LY294002 2μM treatment and IF stained for DAPI, TEAD4, SDC1, and KRT7. Arrows mark areas of SDC1 expression (n=3 over 3 passages, cell line CT3). Note hollow cavities and lack of SDC1^{+} regions in the presence of LY294002.

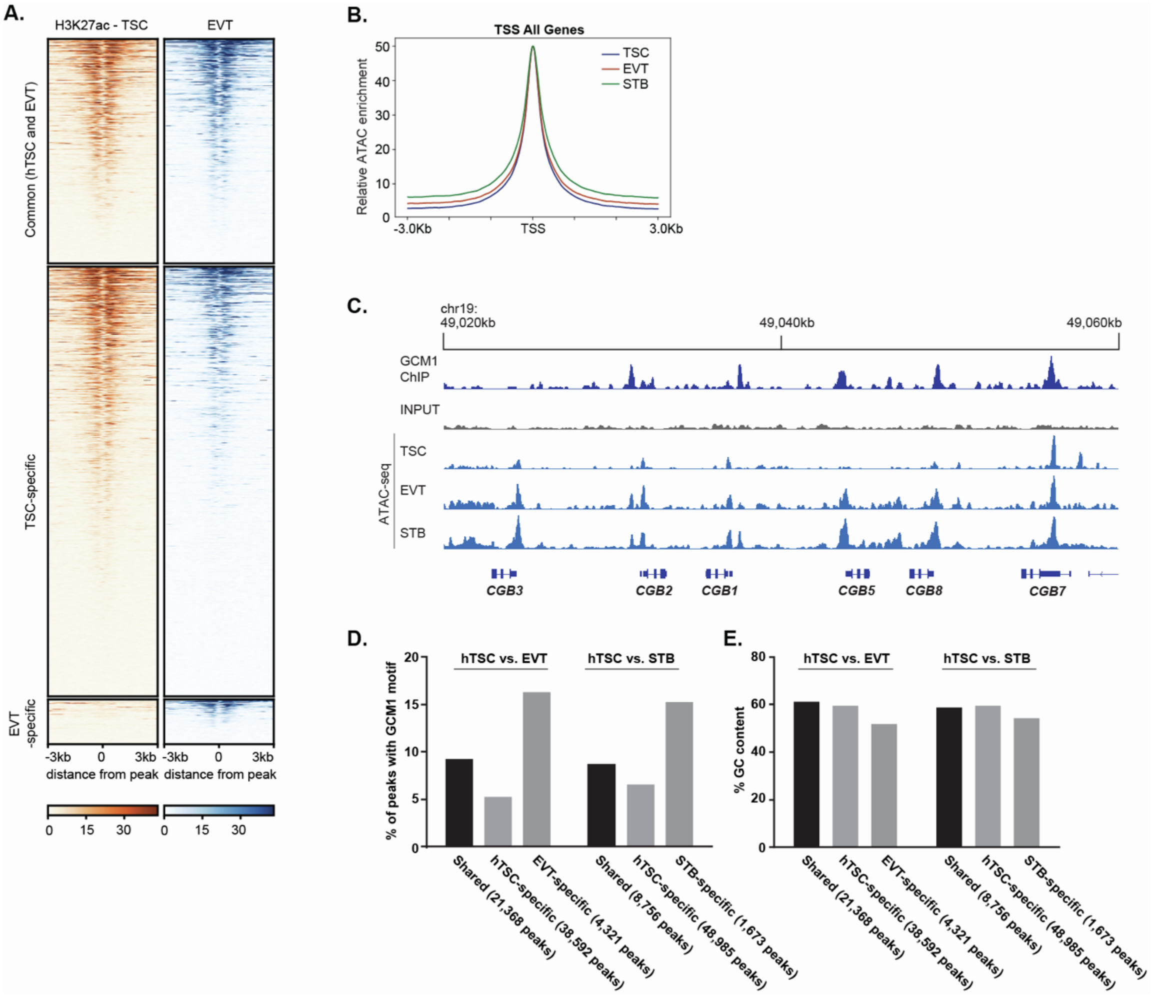

Figure S3 (related to Figure 3). GCM1 positively regulates differentiation-associated genes. **A.** Heatmap of H3K27Ac enrichment over common, hTSC-specific, and EVT-specific ATAC-seq peaks in hTSC and EVT. Note correspondence of H3K27Ac enrichment with ATAC enrichment in each set. **B.** Metaplot of ATAC-seq data from TSC, EVT and STB over all gene TSS after normalization. **C.** GCM1 ChIP-seq and ATAC-seq data plotted over the *CGB* locus. **D.** Percentage of peaks in each category containing GCM motifs. **E.** GC content of ATAC-seq peaks in each category. GCM1 has a GC-rich motif, but the higher frequency of GCM1 sites observed in **(D)** cannot be explained by difference in underlying GC richness.

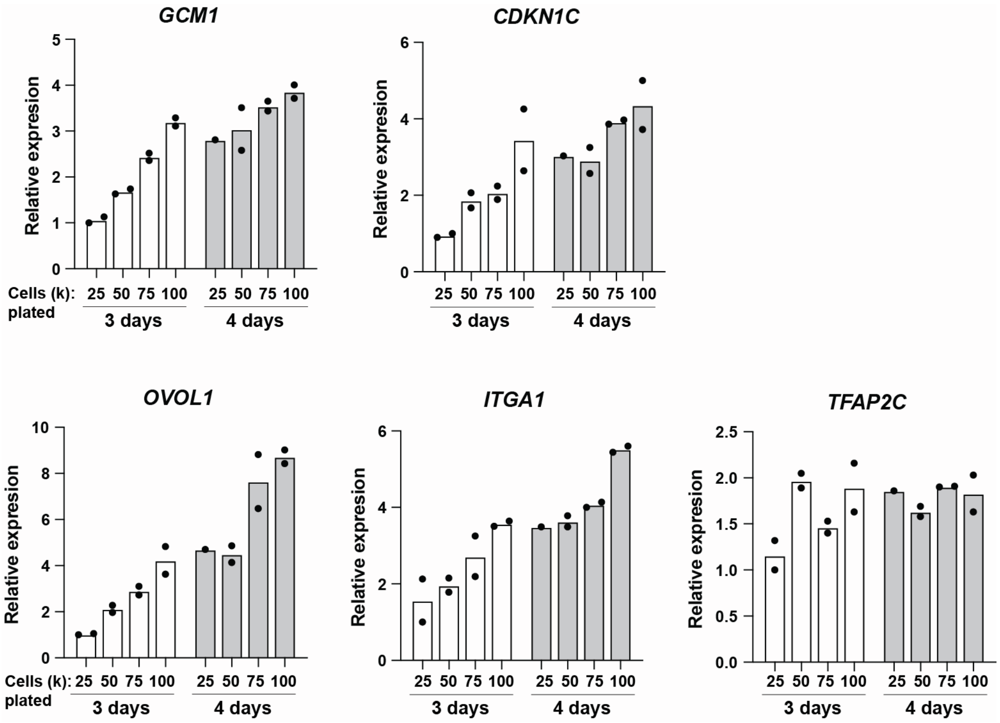

Figure S4 (related to Figure 4). Expression of GCM1 is confluence dependent. Expression of genes indicated in plating conditions (cell number and growth time) indicated. Note that plating at higher densities leads to higher expression of *CDKN1C, GCM1*, *OVOL1* and *ITGA1* (CT3 cells, n=2 replicates).

Supplemental Methods

Cell Culture-Maintenance of hTSC: TSC were cultured in TSC basal media containing DMEM F-12 (GIBCO), 1x ITS-X (GIBCO), 0.3% BSA (WISENT), 1% Penicillin/Streptomycin (GIBCO), 1% ESC qualified Fetal Bovine Serum (GIBCO), 0.1mM β-mercaptoethanol (GIBCO), 15μg/ml L-ascorbic acid (SIGMA), and 50ng/ml recombinant hEGF (GIBCO). To this, 0.75mM valporic acid, 2μM CHIR99021 (Cayman Chem), 0.5μM A8301 (Cayman Chem), 1μM SB431542 (Cayman Chem), and 5μM Y27632 (Cayman Chem) was added to make TSC Media (TSCM) immediately before use. TSCs were dissociated using TrypLE (GIBCO) diluted with PBS to 30% and incubated at 37°C for 10 min. TrypLE was deactivated using 1:1 vol of 0.5mg/ml Soybean trypsin inhibitor (GIBCO) diluted in PBS. For experiments performed in Figure 1, hTSCs were passaged on to 5μg/ml of Collagen IV (Corning) coated plates, but due to product availability, TSC from experiments in Figures 2-4 were passaged onto Laminin 511(SIGMA) coated plates.

Cell Culture-Differentiation of hTSC to EVT: TSCs were converted to EVTs using a modified TSC basal media containing DMEM F-12 (GIBCO), 1x ITS-X (GIBCO), 0.3% BSA (WISENT), 1% Penicillin/Streptomycin, and 0.1mM β-mercaptoethanol (GIBCO). For days 1-2, TSCs were passage onto 1μg/ml Collagen IV coated plates in mod. TSC basal with added 5% Knockout-serum replacement (KSR) (GIBCO), 5μM Y27632 (Cayman Chem), 3μM A8301 (Cayman Chem), 100ng/ml NRG1 (NEB) and 2% GFR-Matrigel (Corning) while the media is still cold (EVTM). Days 3-5, the media is changed to ETVM without NRG1 and reduction to 0.5% GFR-Matrigel. Days 6-8, the media is changed to the previously mentioned ETVM but this time without NRG1, KSR, and reduction to 0.5% GFR-Matrigel. At the end of day 8, differentiated EVTs can be assessed by Flow cytometry.

Cell Culture-Differentiation of hTSC to STB: TSCs were converted to STB3Ds using a modified TSC basal media containing DMEM F-12 (GIBCO), 1x ITS-X (GIBCO), 0.3% BSA (WISENT), 1% Penicillin/Streptomycin, and 0.1mM β-mercaptoethanol (GIBCO). For days 1-2, TSCs were passage on to suspension plate (Sarstedt) in modified TSC basal media containing 5μM Y27632, 2μM Forskolin (Cayman Chem), 5% KSR (GIBCO), and 50ng/ml recombinant hEGF (STBM). On day 3, cell clusters were collected and pulsed-spun for 30sec to separate single cells from STB fusion-clusters and replated in STBM. At day 5, STB-3D are ready to be assessed by immunofluorescence and/or hCG- ELISA.
To generate STB2D, the same media was used except that hEGF was omitted. hTSCs were seeded onto tissue culture plates pre-treated with 2.5μg/uL Collagen IV. Media was changed on day 3, and STB2D were analyzed on day 5.

Cell culture – standard maintenance and EVT differentiation of trophoblast organoids: Villous cytotrophoblasts (vCTBs) were isolated (6 – 7th week of gestation, n=3), and 1 x 10^5 cells were embedded in Matrigel and cultured in Advanced DMEM/F12 (Invitrogen) supplemented with media containing 10mM HEPES, 1 x B27 (Gibco), 1 x ITS-X (Gibco), 2mM glutamine (Gibco), 0.05 mg/ml gentamicin (Gibco), 1 μM A8301 (R&D Systems), 50 ng/ml recombinant human epidermal growth factor (rhEGF, R&D Systems), 3 μM CHIR99021, and 5 μM ROCKi (Y27632, Santa Cruz). After the first passaging, ROCKi was omitted. The medium was changed every 2 – 4 days, and TB-ORG were split after 5 – 7 days. To test effects of LY294002 under stemness conditions, TB-ORG of passage 2 were treated with DMSO (vehicle) or 5 μM LY294002 for 10 days. Culture media were changed every 2 – 3 days.

For EVT differentiation, passage 2 TB-ORG were incubated with TB-ORG medium lacking CHIR99021 (TB-ORG-DIFF). To test the effects of LY294002 on EVT lineage formation and differentiation, TB-ORG were incubated in TB-ORG-DIFF medium supplemented with DMSO (vehicle) or 5 μM LY294002 for 10 days. EVT formation was monitored and bright field images were taken every 2 – 3 days.

Flow cytometry: Dissociated single cells were first washed with 1% KSR-PBS solution. Cell samples were incubated with fluorescently conjugated antibodies for 15min at room temperature. Post-incubation, the samples were washed once with 1% KSR-PBS, then resuspended in 1% KSR-PBS containing DAPI nuclear counterstain to identify live or dead cells. Data acquisition was preformed using the LSR Fortessa and data analysis was performed on FlowJo v10. Antibody: EPCAM (Biolegend324239), ITGA1 (Biolegend328313), ITGA6 (Biolegend313631), and HLA-G (ab24384).

Western blot: Dissociated single cell samples were washed with cold PBS and lysed with Laemmli buffer without blue dye and boiled at 95°C for 5 minutes. Protein concentration was determined using a standard Bradford assay. Standard Bio-RAD SDS-PAGE system was used to separate proteins and transfer it to PVDF membrane (Millipore). Membrane blocking, primary and secondary antibody incubations are diluted in Odyssey Blocking buffer (LICOR). Infrared conjugated secondary antibodies were used for detection and visualization of the protein of interest on the membrane with the LICOR-Odyssey Imager. Antibody: GCM1 (ab187860, 49kDA), TFAP2C (ab76007, 49kDA), Histone H3 (ab10799, 15kDA), and TEAD4 (ab58310, 48kDA). Figure 4D was performed with 2 blots of the same protein samples.

Immunofluorescence: Glass coverslips were precoated with 5μg/ml Collagen IV (CORNING) overnight before cell attachment. Cells were grown for a determined amount of time for its corresponding experiment. Coverslips were fixed with 4% PFA for 20 min. at room temperature. PFA solution was washed 3 times with PBS before permeabilization with permeabilization buffer (PBS + 5% donkey serum + 0.1% Trition-X100) for 30 min. Primary and secondary antibodies were diluted in permeabilization buffer and incubated for 1-2 hours for each process. Coverslips were washed with PBS containing DAPI nuclear counterstain and mounted on glass slides using Pro Long Gold (Invitrogen). Imaging analysis was performed using the Axiovert (Zeiss). Antibody: GCM1 (ab187860), hCGB (ab131170) ITGA2 (Biolegend108901), ITGA5 (ab150361), KRT7 (BioLegend601603), NOTCH1 (CS4380), SDC1 (HPA006185), TEAD4 (ab58310), and TFAP2C (ab76007).

RNA isolation and qPCR: Total RNA isolation used manufacturing protocol indicated by Sigma-Aldrich RNAzol^{RT} R4533. Qubit™ RNA BR Assay kit (Q10211) was used to measure RNA concentration.
First-strand cDNA was generated using the SensiFast cDNA Synthesis kit (Froggabio). Quantitative PCR was done using PowerUP SYBR^{TM} Green PCR (Invitrogen, A25742) on Quantstudio 5 (Applied Biosystems) with the following cycling conditions: 50 °C 2 minutes, 95 °C 20 seconds, 45x (95 °C 3 seconds, 60 °C 30 seconds), 95 °C 1 second). The qPCR reaction was performed using 1x concentration of PowerUP SYBR Green Master Mix, 5ng of template mRNA and 0.5 μM of primer mix in a total of 7 μL reaction. The expression of target genes was

normalized to the housekeeping gene *RAB7A* and the sequence of primer used for qPCR are provided in the table below.

**qPCR primer name**	**sequence**
GCM1_F	TGAACACAGCACCTTCCTCC
GCM1_R	CGCCTTCCTGGAAAGACCAA
RAB7A_3_F	GAGGTGGAGCTGTACAACGAATTT
RAB7A_3_R	CGGTCATTCTTGTCCAGTTTGATAG
CDKN1C (p57KIP2)_F	AGCTGCACTCGGGGATTTC
CDKN1C (p57KIP2)_R	ACTTCTCAGGCGCTGATCTCTT
OVOL1_1F	CAATGACACCTTCGACCTCA
OVOL1_1R	TGCACACCATGGATCTTCTT
ITGA1_F	ACGCTGCTGCGTATCATTCA
ITGA1_R	CACCTCTCCCAACTGGACAC
TFAP2C_F	CGCGGAAGAGTATGTTGTTG
TFAP2C _R	TATGTTCGGCTCCAAGACCT
VHL_F4_CR	GACCTGGAGCGGCTGACA
VHL_R4_CR	TACCATCAAAAGCTGAGATGAAACA

RNA isolation and qPCR from TB-ORG (Figures 2N,O). TB-ORG were washed with ice-cold PBS and re-suspended with PeqGold Trifast (PeqLab). Homogenization of TB-ORG was supported using the Precellys 24 (CK-Mix tubes, 5000 rpm, 1 x 20 sec, PeqLab) and RNA isolation was performed as indicated by the manufacturer. RNA (1 µg per sample) was reverse transcribed (RevertAid H Minus Reverse Transcriptase, Thermo Scientific) and qPCR was performed (7500 Fast Real-time PCR system, Applied Biosystems). The following TaqMan Gene Expression Assays (ABI) were used: *CGB* (Hs00361224_g), *ENDOU* (Hs00195731_m1), *TP63* (Hs00978340), *HLA-G* (Hs00365950_g1), and *ITGA1* (Hs00235006_m1). Signals (ΔCt) were normalized to TATA-box binding protein (*TBP*, 4333769F).

Library preparation: For RNA library synthesis, in brief, purified mRNA was cleaned using the NEBNext Poly(A) mRNA Magnetic Isolation Module kit (NEB E7490) and final library generation was created with the NEBNext® Ultra RNA Library Prep Kit for Illumina® (NEB E7530) following manufacturing instructions. Barcoding came from TruSeq Unique Dual Indexes (Illumina, San Diego, CA). Qubit™ 1x dsDNA HS Assay kit (Q33231) was used to measure synthesis of the library.

Chromatin immunoprecipitation: Cell samples are fixed with 0.66% paraformaldehyde (FisherSci) diluted with PBS and is quenched by adding glycine to a final concentration of 0.125M. Nuclear lysis extraction begins with nuclear lysis buffer (50mM HEPES pH 7.8, 0.5% Triton X-100, 1mM EDTA, 0.5mM EGTA, 140 mM NaCl, 10% glycerol and 1% NP-40). Nuclei are resuspended in nuclear wash buffer (10mM Tris-HCl pH 8.0, 200mM NaCl, 1mM EDTA, and 0.5mM EGTA). Nuclear pellets are then resuspended in SDS lysis buffer (50mM Tris-HCl pH 8, 10mM EDTA, 1% SDS, and 1% Triton X-100). Nuclei samples are transferred to a 1ml tube (Bioruptor) and keep cold before sonication. Sonication was performed using the Diagenode Bioruptor sonicator (settings: 30sec on, 30 sec off, 30-35 cycles). Then diluted with Dilution buffer (25mM Tris-HCl pH 8, 150 mM NaCl, 3mM EDTA, and 1% Triton X-100). A pre-clearing step is performed by using 40µl of pre-washed Protein G Sepharose beads (SIGMA, P3296-5ml)

combined sonicated sample. 2µg of GCM1(HPA001343) antibody is added to each pre-cleared sample and then rotated overnight at 4°C. Pre-washed Protein G Sepharose beads are added to the ChIP samples and washed with three buffers: DB150 (25mM Tris-HCl pH 8.0, 150mM NaCl, 3mM EDTA, 1% Triton X-100, and 0.05% SDS), DB500 (25mM Tris-HCl pH 8.0, 500mM NaCl, 3mM EDTA, 1% Triton X-100, and 0.05% SDS), Buffer III (10mM Tris-HCl pH 8.0, 250mM LiCl, 1% Sodium Deoxycholate, 1% NP-40, and 1mM EDTA, and lastly TE buffer (10mM Tris-HCl pH 8.0, 1mM EDTA). DNA is eluted with 200µl elution buffer (100 mM NaHCO3, and 1% SDS) and incubated at 65°C overnight for decrosslinking. Ethanol precipitation is performed to collect ChIP material, and further purification was performed with Geneaid Gel/PCR cleanup protocol. Purified DNA fragments passed to sequencing library preparation.

ATAC-Seq: ATAC-seq library preparation was performed on 1×10^6 freshly cultured cells using a commercially available ATAC-Seq kit from Active Motif (#53150, Carlsbad, CA). We followed manufacturers protocol with some minor modification. Each sample was lysed in the ATAC-seq lysis buffer. Next, the samples were processed for the transposase reaction. After cleanup of the transposed DNA, samples were stored at −20 °C until library amplification. Samples were subsequently thawed at room temperature and library construction completed according to manufacturer protocol. Libraries were quantified using a Qubit dsDNA BR Assay Kit (Q32853, Thermo-Fisher) and the size was determined with a High Sensitivity DNA Bioanalyzer Kit (5067-4626, Agilent, Santa Clara, CA) and sequenced on a NovaSeq 6000 (Illumina, San Diego, CA) using Nextera Sequencing primers.

Supplemental Table Captions

Table S1: Gene expression associated with different oxygen concentrations, including FPKM of all samples, differentially expressed genes for 2% and 5% O_2 as compared with 20% O_2, and gene cluster analysis to identify genes associated with O_2 level.
Table S2: Expression of genes specific to hTSC, EVT and STB in hTSCs grown at 20%, 5% and 2% O_2.
Table S3: Gene expression associated with WT and GCM1 KO lines, including FPKM, expression of hTSC, STB and EVT-specific genes, and genes identified as differentially expressed between WT vs. GCM1 KO.
Table S4: GCM1 ChIP-seq peaks as well as ATAC-seq peaks specific to cell types indicated.
