# Supplementary material for: Hypoxia and loss of GCM1 expression prevent differentiation and contact inhibition in human trophoblast stem cells
Source: Stem Cell Reports. 2025 Apr 24;20(5):102481. doi: 10.1016/j.stemcr.2025.102481 (PMC12143156; doi:10.1016/j.stemcr.2025.102481)
Supplement: Document S2. Article plus supplemental information [file mmc6.pdf]

Stem Cell Reports
Report
ISSCR
OPEN ACCESS

Hypoxia and loss of *GCM1* expression prevent differentiation and contact inhibition in human trophoblast stem cells

Jessica K. Cinkornpumin,[1] Sin Young Kwon,[1] Anna-Maria Prandstetter,[2] Theresa Maxian,[2] Jacinthe Sirois,[1,3] James Goldberg,[1] Joy Zhang,[1] Deepak Saini,[1] Purbasa Dasgupta,[5] Mariyan J. Jeyarajah,[4] Stephen J. Renaud,[4] Soumen Paul,[5,6,7] Sandra Haider,[2] and William A. Pastor[1,3,8,*]
[1]Department of Biochemistry, McGill University, Montreal, QC, Canada
[2]Placental Development Group, Reproductive Biology Unit, Medical University of Vienna, Vienna, Austria
[3]The Rosalind & Morris Goodman Cancer Institute, McGill University, Montreal, QC, Canada
[4]Department of Anatomy and Cell Biology, Schulich School of Medicine and Dentistry, University of Western Ontario, London, ON, Canada
[5]Department of Pathology and Laboratory Medicine, University of Kansas, Kansas City, Kansas, USA
[6]Institute for Reproduction and Developmental Sciences, University of Kansas, Kansas City, Kansas, USA
[7]Department of Obstetrics and Gynecology, University of Kansas, Kansas City, Kansas, USA
[8]Lead contact
*Correspondence: william.pastor@mcgill.ca
https://doi.org/10.1016/j.stemcr.2025.102481

SUMMARY

During the first stages of embryonic development, the placenta develops under very low oxygen tension (~1%–2% O_2), so we sought to determine the regulatory role of oxygen in human trophoblast stem cells (hTSCs). We find that low oxygen promotes hTSC self-renewal but inhibits differentiation to syncytiotrophoblast (STB) and extravillous trophoblast (EVT). The transcription factor GCM1 (glial cell missing transcription factor 1) is downregulated in low oxygen, and concordantly, there is substantial reduction of GCM1-regulated genes in hypoxic conditions. Knockout of GCM1 in hTSC likewise impaired EVT and STB formation. Treatment with a phosphatidylinositol 3-kinase (PI3K) inhibitor reported to reduce GCM1 protein levels likewise counteracts spontaneous or directed differentiation. Additionally, chromatin immunoprecipitation of GCM1 showed binding near key genes upregulated upon differentiation including the contact inhibition factor *CDKN1C*. Loss of *GCM1* resulted in downregulation of *CDKN1C* and corresponding loss of contact inhibition, implicating GCM1 in regulation of this critical process.

INTRODUCTION

During the first major specification event in embryonic development, the outer cells of the developing blastocyst are specified as trophectoderm (TE) (Chazaud and Yamanaka, 2016). Cells from the TE, upon implantation, give rise to cells called cytotrophoblasts (CTBs), which can differentiate into the extravillous trophoblast (EVT) and syncytiotrophoblast (STB). These cells are organized into structures called villi, in which CTBs line the inside of the villus and STBs line the outside, mediating the exchange of nutrients, oxygen (O_2), and waste. At the tips of the villi, the points of contact with maternal tissue, the CTBs form a cell column and differentiate into EVTs. Distinct subtypes of mature EVTs act to invade maternal decidua and to remodel spiral arteries, enabling proper blood flow to the placenta (Turco et al., 2018).

During the first trimester of human pregnancy, some EVTs establish plugs blocking the uterine spiral arteries. For the initial stages of the first trimester, the conceptus develops in a low-oxygen environment (~2.5% O_2, hypoxia) (Jauniaux et al., 2001; Rodesch et al., 1992). After approximately 8 weeks, the trophoblast plugs disintegrate, and endovascular extravillous cytotrophoblasts invade the uterine spiral arteries where they degrade smooth muscle and replace the resident endothelial cells (Sato, 2020). This expands the arterial lumen, provides blood to the placenta, and raises oxygen tension (~8.6% O_2) (Chang et al., 2018; Genbacev et al., 1997; Rodesch et al., 1992).

Oxygen tension is clearly important in regulation of the trophoblast, but how it regulates placental cell self-renewal and differentiation is not entirely clear. Aspects of response to hypoxia are universal. At high oxygen levels, one of several prolyl hydroxylases will oxidize the hypoxia-inducible factor (HIF) family transcription factors, HIF1α and HIF2α. The oxidized prolines are then recognized by von Hippel-Lindau tumor supressor (VHL), which ubiquitinates the HIFs and targets them for destruction (Majmundar et al., 2010). Low oxygen reduces the activity of the prolyl hydroxylases and thus stabilizes HIF family transcription factors. HIF1α or HIF2α then dimerizes with the transcription factor aryl hydrocarbon receptor nuclear translocator (ARNT) and promotes transcription of target genes. Certain HIF targets are consistent across cell types, and hypoxia frequently promotes angiogenesis and a shift from oxidative respiration to glycolysis (Majmundar et al., 2010). With regard to placenta, mice carrying deletions of *Hif1α/Hif2α* or *Arnt* both undergo midgestational embryonic lethality (Cowden Dahl et al., 2005; Kozak et al., 1997). These mutants show reduced labyrinth

© 2025 The Authors.
Published by Elsevier Inc. on behalf of International Society for Stem Cell Research.
This is an open access article under the CC BY license (http://creativecommons.org/licenses/by/4.0/).
Check for updates

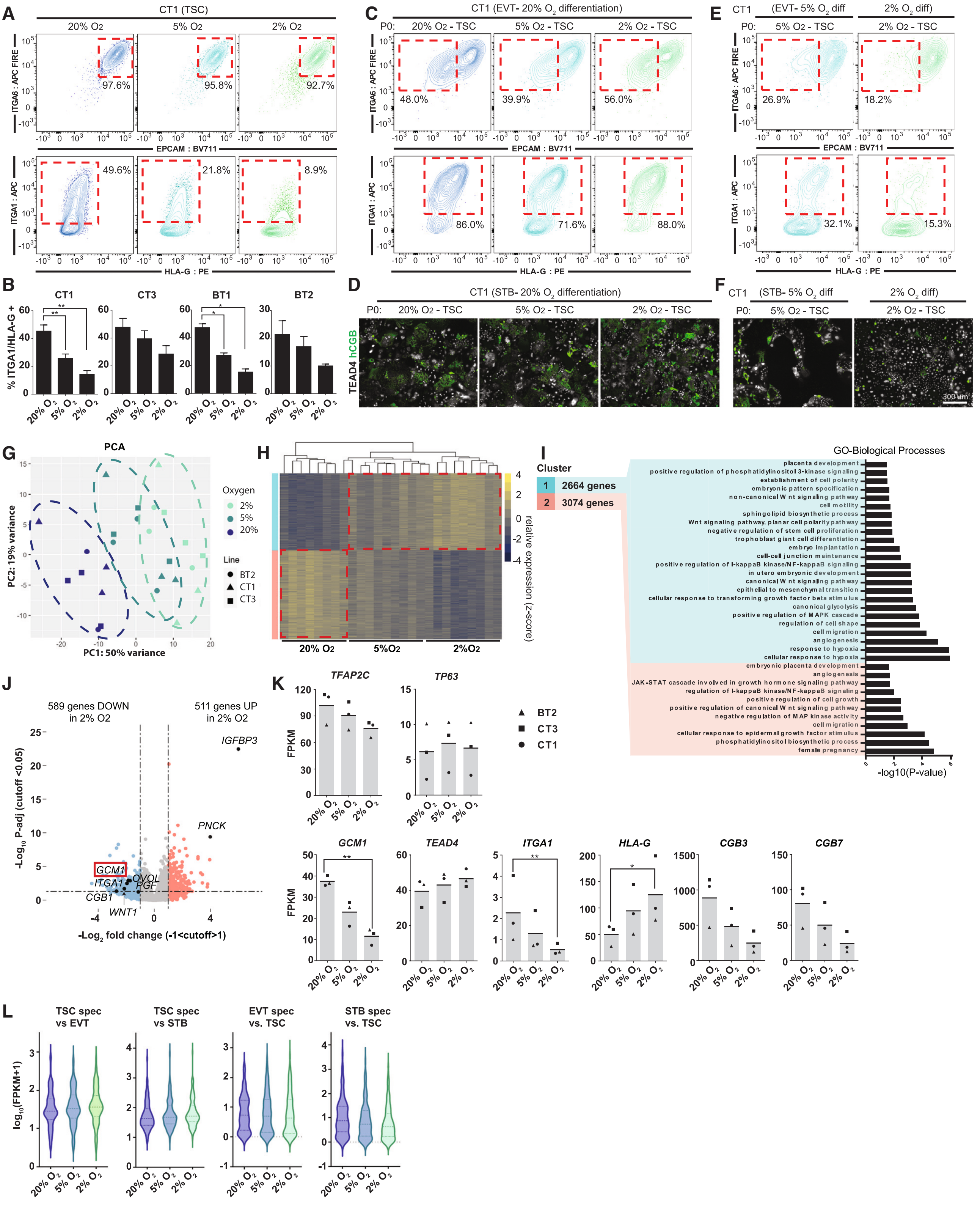

(legend on next page)

vascularization, consistent with the classic role for hypoxia signaling in angiogenesis (Cowden Dahl et al., 2005; Kozak et al., 1997). Intriguingly, they also fail to maintain their spongiotrophoblast population (the murine functional equivalent to EVT precursors), and *Hif1a*$^{-/-}$*Hif2a*$^{-/-}$ or *Arnt*$^{-/-}$ murine trophoblast stem cells preferentially differentiate to STB rather than spongiotrophoblast lineage (Cowden Dahl et al., 2005). Loss of the prolyl hydroxylase *Phd2*, which causes elevated HIF stability, results in reduced expression of STB markers and an increase in spongiotrophoblast (Takeda et al., 2006), while a *Vhl*$^{-/-}$ mouse features a loss of STB altogether (Gnarra et al., 1997). Thus, a consistent feature in murine models is that hypoxia is unfavorable to STB differentiation but conducive for spongiotrophoblast.

In humans, hypoxia is well established to block differentiation to STBs (Alsat et al., 1996; Jaremek et al., 2023; Nelson et al., 1999; Wakeland et al., 2017). The effects on EVT differentiation are less clear. When explants of human placental villi are cultured in high oxygen, expression of integrin subunit alpha 1 (ITGA1, a mature EVT marker) is observed at the edges of the explant where cell column CTBs are found. In low oxygen, instead of ITGA1 expression, cell column CTBs proliferated and appeared to show elevated human leukocyte antigen (HLA)-G (Genbacev et al., 1997). Low O_2 is reported to reduce CTB invasiveness and block the expression of ITGA1, further supporting a role for oxygen in positive regulation of EVT differentiation (Genbacev et al., 1996). Another study reports higher HLA-G upon culture of human CTB at low O_2 and indicates a positive role for hypoxia in promoting conversion of CTB to less mature, proximal column EVT (Wakeland et al., 2017). With the discovery of culture conditions that allow for indefinite culture of CTBs *in vitro* as human trophoblast stem cells (hTSCs) (Okae et al., 2018), we sought to determine the molecular and phenotypic effects of oxygen concentration on human placental cells.

RESULTS

Hypoxia maintains trophoblast stemness

To determine the effect of hypoxia on hTSC growth, we cultured hTSCs in 20%, 5%, and 2% O_2. After 72 h of culture, we performed flow cytometry for the hTSC cell surface markers ITGA6 and EpCAM and the EVT markers ITGA1 and HLA-G (Figure 1A). We observed noticeable depletion of ITGA1 and slightly increased HLA-G expression in the lower oxygen concentrations in several hTSC lines (Figures 1A and 1B), similar to what was observed in explants by Genbacev and colleagues. Continued culture of these cells in their respective oxygen conditions resulted in near complete loss of ITGA1^{hi} expression cells in both 5% and 2% O_2 (Figure S1A). Similarly, hTSCs cultured in 20% O_2 showed some spontaneous expression of the STB marker hCGB, which was reduced in low oxygen (Figures S1B and S1C). Regions of dense cell-to-cell contact showed expression of the differentiation marker NOTCH1 in hTSCs at 20% O_2 but not in lower oxygen (Figure S1D). In addition to lower expression of

Figure 1. Reduced and impaired hTSC differentiation in hypoxic conditions

(A) Trophoblast stem cells were cultured for 72 h in varying levels of oxygen (20%, 5%, 2% O_2). Flow cytometry plots indicate levels of hTSC (ITGA6 and EPCAM) and EVT (ITGA1 and HLA-G) markers. Note reduction in ITGA1$^+$ population in low O_2.
(B) ITGA1$^+$ HLA-G$^+$ population in O_2 and cell line indicated (4 cell lines, $n = 3$ replicates for each cell line at 3 different passages). Statistical significance was determined via a two-tailed t test.
(C) EVT differentiation in 20% O_2 starting with hTSC in oxygen concentration indicated. Successful differentiation is indicated by the upregulation of surface markers ITGA1 and HLA-G and downregulation of EPCAM and ITGA6.
(D) STB differentiation in 20% O_2 starting with hTSC in oxygen concentration indicated. STB formation is indicated by loss of TEAD4 and increase in hCGB staining in a cell.
(E) EVT differentiation undertaken at oxygen level indicated.
(F) STB differentiation undertaken at oxygen level indicated.
(G) Principal component analysis (PCA) showing gene expression from hTSC cultured in varying oxygen concentrations. Ovals encompassing all 2%, 5%, and 20% O_2 samples are drawn manually.
(H) Hierarchical gene clustering of RNA-seq samples in (G). Red dotted lines indicate the shift in gene expression from 20% O_2 and 2% O_2 labeled as cluster 1 and cluster 2.
(I) Gene set enrichment analysis (GSEA) analysis of cluster 1 and cluster 2.
(J) Volcano plot showing gene expression differences between TSCs cultured in 20% O_2 to TSCs cultured in 2% O_2. Dashed lines indicate significance and log2 fold change cutoff.
(K) Bar graphs showing FPKM of specific genes of interest (same samples as in G, significance indicated corresponds to p_{adj} values from DESEQ2 analysis, see Table S1).
(L) Violin plot showing expression of genes specific to hTSC, EVT, or STB for hTSCs grown in the indicated oxygen concentration. When comparing log_2 fold change between genes in each set, differences between all sets at all oxygen concentrations are significant ($p < 0.001$) (For analysis in G–L, 3 cell lines; BT2, CT1, CT3; $n = 3$ replicates for each line in each condition over 3 passages, except BT2 at 20% O_2 $n = 2$).

differentiation markers, we observed higher cell density in lower oxygen culture conditions (Figure S1E). Collectively, these results indicated that low oxygen aids in stemness and proliferation, while high oxygen promotes spontaneous differentiation.

Based on these findings, we cultured hTSCs and conducted directed differentiation at varying oxygen conditions. hTSC cultured in 20%, 5%, or 2% O_2 successfully differentiated to EVT or STB if differentiation was undertaken at 20% O_2 (Figures 1C and 1D). However, when we performed EVT or STB differentiation at reduced oxygen levels, we observed dramatic impairment of differentiation (Figures 1E, 1F, and S1F). EVTs differentiated in reduced oxygen failed to downregulate EpCAM or upregulate ITGA1 and HLA-G (Figure 1E), while STBs in 2% O_2 showed a higher percentage of cells retaining the stem cell marker TEA domain transcription factor 4 (TEAD4) and a lower percentage expressing the STB marker Chorionic Gonadotropin beta chain (hCGB) (Figures 1F and S1F). Thus, oxygen promotes both spontaneous and directed differentiation of hTSCs.

Trophoblast differentiation transcription factor, GCM1, is oxygen sensitive

We performed RNA sequencing (RNA-seq) on three hTSC lines (CT1, CT3, and BT2; female) cultured in 20%, 5%, or 2% O_2. Principal component analysis (PCA) and a correlation matrix show a strong dependence of gene expression on oxygen tension (Figures 1G and S1G). Gene cluster and enrichment analysis (Sherman et al., 2022) indicate that the genes in both cluster 1 (genes upregulated in 2% O_2) and cluster 2 (upregulated in 20% O_2) showed general enrichment for various placental-related terms while only cluster 1 corresponded to hypoxia response and WNT activation (Figures 1H and 1I).

Genes associated with EVT and STB expression such as *ITGA1* and *OVOL1* and various chorionic gonadotropin genes were among the genes less expressed in the 2% O_2 condition (Figures 1J, 1K, and S1H; Table S1). Using published gene expression data, we identified 100 genes specific to EVT and STB differentiation and 100 genes specific to hTSC relative to STB and EVT (Okae et al., 2018). hTSC genes were higher in 2% O_2, while EVT and STB genes were lower, further indicating that hypoxia broadly suppresses genes associated with differentiation (Figure 1L; Table S2). Interestingly, consistent with flow cytometry data (Figure 1A), HLA-G was positively regulated by hypoxia (Figure 1K), indicating that hypoxia promotes expression of this differentiation marker even as it suppresses the overall EVT differentiation program.

Analysis of known transcription factor (TF) targets appropriately indicated that HIF1α was the most enriched TF associated with cluster 1 (hypoxia) expression, while *GCM1* (glial cells missing TF 1) was associated with high oxygen concentration (Figures S1I and S1J). *GCM1*, which is expressed in hTSCs, but upregulated upon differentiation (Figure S1K), was itself strongly downregulated in low oxygen conditions both at the RNA and protein level (Figures 1J, 1K, and S1L; Table S1). GATA3, reported to repress GCM1 by an indirect mechanism (Wang et al., 2022), was upregulated in 2% O_2, but only slightly, suggesting another mechanism at work (Figure S1H). To determine if GCM1 is regulated by the canonical hypoxia pathway, we used CRISPR interference to repress *VHL*, the ubiquitin ligase that targets HIF proteins for destruction. Repression of *VHL* in 20% O_2 led to dramatic upregulation of *IGFBP3*, the most hypoxia-responsive gene in hTSC and a known HIF1α target (Natsuizaka et al., 2012). We also observed downregulation of *GCM1* expression, confirming that *GCM1* is negatively regulated by canonical hypoxia response (Figure S1M).

GCM1 is essential for the differentiation into trophoblast lineages

Since GCM1 is highly sensitive to oxygen concentration and is implicated in hTSC differentiation (Wang et al., 2022), we generated GCM1-knockout hTSC (GCM1 KO1) by deleting a small genomic region in exon 2 just after the ATG start site to disrupt the translation of the DNA-binding domain (Chiu and Chen, 2016) and subsequent protein sequence (Figure 2A). Unexpectedly, this deletion made an alternative splice site available, which spliced in before exon 3 (Figure S2A). While this deletion still had the desired frameshift, we generated additional lines ($GCM1^{-/-}$ KO2) by deleting the entirety of exon 3 (Figures 2A and S2A). hTSCs electroporated with a non-targeting single-guide RNA (sgRNA) showed ubiquitous nuclear expression of GCM1, with TEAD4 loss in the highest GCM1-expressing cells (Figure 2B), while both $GCM1^{-/-}$ lines showed loss of specific GCM1 signal (Figure 2B). Consistent with recent reports (Jeyarajah et al., 2022; Shimizu et al., 2023; Wang et al., 2022), $GCM1^{-/-}$ hTSC failed to differentiate to EVT or STBs, a result demonstrated by flow cytometry, immunofluorescent staining, and RNA-seq of control and $GCM1^{-/-}$ cells (Figures 2C–2G and S2B–S2F). $GCM1^{-/-}$ hTSCs showed lower expression of EVT and STB-specific genes and substantially failed to upregulate these genes upon directed differentiation (Figure S2G; Table S3).

We then compared the effects of *GCM1* loss to the effects of hypoxia in hTSCs. While they did not cluster precisely together, $GCM1^{-/-}$ cells grown in 20% O_2 showed similar positioning over principal component axis 1 with control hTSCs grown in 2% O_2. This would indicate that a substantial portion of the differential gene expression associated with hypoxia is in fact a consequence of lower GCM1 level

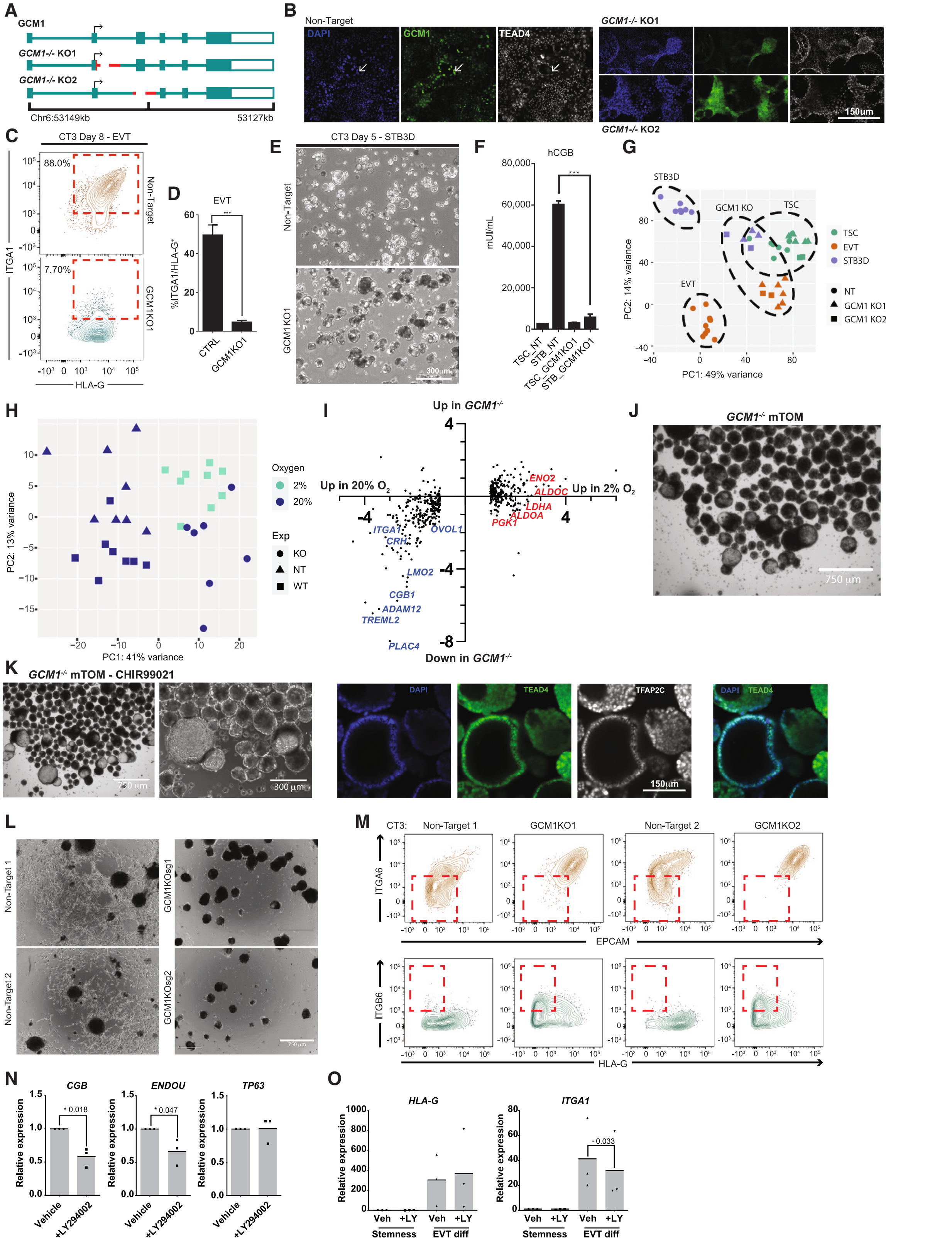

(legend on next page)

(Figure 2H). More specifically, we observe that a high proportion of genes downregulated in 2% O_2, genes associated with trophoblast differentiation, are also downregulated in *GCM1*$^{-/-}$. By contrast, genes upregulated in 2% O_2, such as established HIF targets and factors that promote glycolysis (Majmundar et al., 2010), are generally unaffected in *GCM1*$^{-/-}$ (Figure 2I).

Interesting morphological phenomena were observed for *GCM1*$^{-/-}$ hTSCs. When *GCM1*$^{-/-}$ lines were grown to over-confluency in hTSC media (TSCM), we observed three-dimensional dome-like projections. Much larger domes were observed in modified trophoblast organoid media with CHIR99021 omitted (mTOM-C) EVT precursor media (see methods) (Figure S2H). We then cultured the *GCM1*$^{-/-}$ hTSCs using a modified form of a trophoblast organoid (TB-ORG) culture system. When cultured with standard mTOM media in micro-V-shaped wells with Matrigel omitted to allow free-floating organoids, the *GCM1*$^{-/-}$ trophoblast stem cells (TSCs) formed hollow balls of cells (Figure 2J). Removal of CHIR99021 (mTOM-C) resulted in further growth of these *GCM1*$^{-/-}$ shells (Figure 2K). At high cell number, the formation of branched structures is observed (Figure S2I). Upon directed differentiation to EVT, these *GCM1*$^{-/-}$ TB-ORGs failed to adopt EVT morphology and enter an HLA-G^{hi} EpCAM^{lo} state. Instead they upregulated the surface marker ITGB6 (Figures 2L and 2M). ITGB6 is selectively present in column CTBs (Arutyunyan et al., 2023; Lee et al., 2018b), the last GCM1^{lo} state before differentiation, suggesting failure to differentiate beyond this stage (Figures S2J–S2L). Generally, genes upregulated in *GCM1*$^{-/-}$ cells showed highest expression in villous and cell column CTB, while genes downregulated in *GCM1*$^{-/-}$ were associated with subsequent differentiated states (Figure S2M).

TB-ORGs undergo spontaneous differentiation, forming a core of STB in the middle. We considered whether this could be prevented by reducing the expression of GCM1. A published report in choriocarcinoma cells showed that hypoxia inhibits the phosphatidylinositol 3-kinase (PI3K)/pAKT pathway and that chemical inhibition of PI3K can lead to reduced expression of GCM1 (Chiang et al., 2009). hTSCs treated with the PI3K inhibitor LY294002 showed some formation of dome-like structures akin to what is observed for *GCM1*$^{-/-}$ (Figures S2N and S2O). Likewise, LY294002-treated TB-ORG grown in mTOM-C conditions without Matrigel showed some propensity for the formation of hollow

Figure 2. Impaired differentiation upon genetic or chemical reduction in GCM1 level

(A) Strategies for mutation of GCM1 using a two-sgRNA CRISPR approach. Lines were generated by deletion of the exon2/intron2 boundary, and by ablation of exon 3, either of which should disrupt the DNA-binding domain of GCM1.

(B) Immunofluorescence staining of GCM1 and TEAD4 in control (non-target, NT sgRNA) and *GCM1*$^{-/-}$ hTSC. Sporadic GCM1^{+} TEAD4^{lo} hTSCs are present only in NT control hTSCs.

(C) Flow cytometric analysis from EVT differentiation of GCM1 KO1 and NT control TSC. NT hTSC differentiation produced ITGA1^{hi}/HLA-G^{hi} cells whereas *GCM1*$^{-/-}$ TSC did not.

(D) Bar graphs showing formation of ITGA1^{hi}/HLA-G^{hi} population from control and *GCM1*$^{-/-}$ TSC (2 cell lines, CT1 n = 2 clonal lines, CT3 n = 3 clonal lines for both NT and KO).

(E) 3D STB formation of NT and *GCM1*$^{-/-}$ hTSC. Control hTSCs form a fluid-filled syncytium while *GCM1*$^{-/-}$ hTSCs form a cluster of cells.

(F) hCGB ELISA was performed using supernatant from *GCM1*$^{-/-}$ and control hTSC (2 cell lines, CT1 n = 2 clonal lines, CT3 n = 3 clonal lines for both NT and KO). Statistical significance was determined via a two-tailed t test.

(G) PCA comparing NT and *GCM1*$^{-/-}$ hTSC, EVT, and STB3D. Note that *GCM1*$^{-/-}$ cells regardless of differentiation state cluster closer to the hTSC population, and similarity of *GCM1*$^{-/-}$ lines 1 and 2 (TSC: n = 8 NT, n = 7 KO; EVT: n = 9 NT, n = 8 KO; STB3D n = 7 NT, n = 6 KO clonal replicates). Ovals encompassing WT TSC, STB, and EVT, as well as GCM1 KO STB and EVT, are drawn manually.

(H) PCA of control (NT) and *GCM1*$^{-/-}$ hTSCs, compared with WT hTSCs grown at different O_2 concentrations. Note that *GCM1*$^{-/-}$ hTSCs cluster on principal component axis 1 with WT hTSCs grown at 2% O_2 (n = 8 WT 20% O_2, n = 9 2% O_2, n = 8 NT 20% O_2, n = 7 KO 20% O_2).

(I) Scatterplot of genes differentially regulated in hypoxia (same set as Figure 1J) showing their relative expression in 2% and 20% O_2 and their relative expression in *GCM1*$^{-/-}$ hTSC and control cells. Examples of placental differentiation genes are shown in blue, while genes involved in glycolysis are shown in red.

(J) Bright-field images of *GCM1*$^{-/-}$ TB-ORG cultured in mTOM media.

(K) Left: Bright-field images of *GCM1*$^{-/-}$ TB-ORG culture in mTOM media-CHIR99021. Right: Immunofluorescent staining for trophoblast markers in *GCM1*$^{-/-}$ TB-ORG (representative of n = 5 images for NT and KO).

(L) NT and *GCM1*$^{-/-}$ TB-ORG differentiated to EVT.

(M) Flow cytometry of NT and *GCM1*$^{-/-}$ TB-ORG differentiated to EVT. *GCM1*$^{-/-}$ hTSCs fail to upregulate the EVT marker HLA-G but do upregulate the cell column marker, ITGB6 (representative image, n = 5 for NT and KO).

(N) Expression of genes associated with differentiation (*CGB*, *ENDOU*) or stemness (*TP63*), normalized to the housekeeping gene *TBP*, in steady-state TB-ORG conditions with 5 μM LY294002 or vehicle control (n = 3 cell line replicates). Statistical significance was calculated using a one-tailed t test.

(O) Expression of EVT genes upon differentiation to EVT with 5 μM LY294002 or vehicle control (n = 3 cell lines replicates). Statistical significance was calculated using a one-tailed t test.

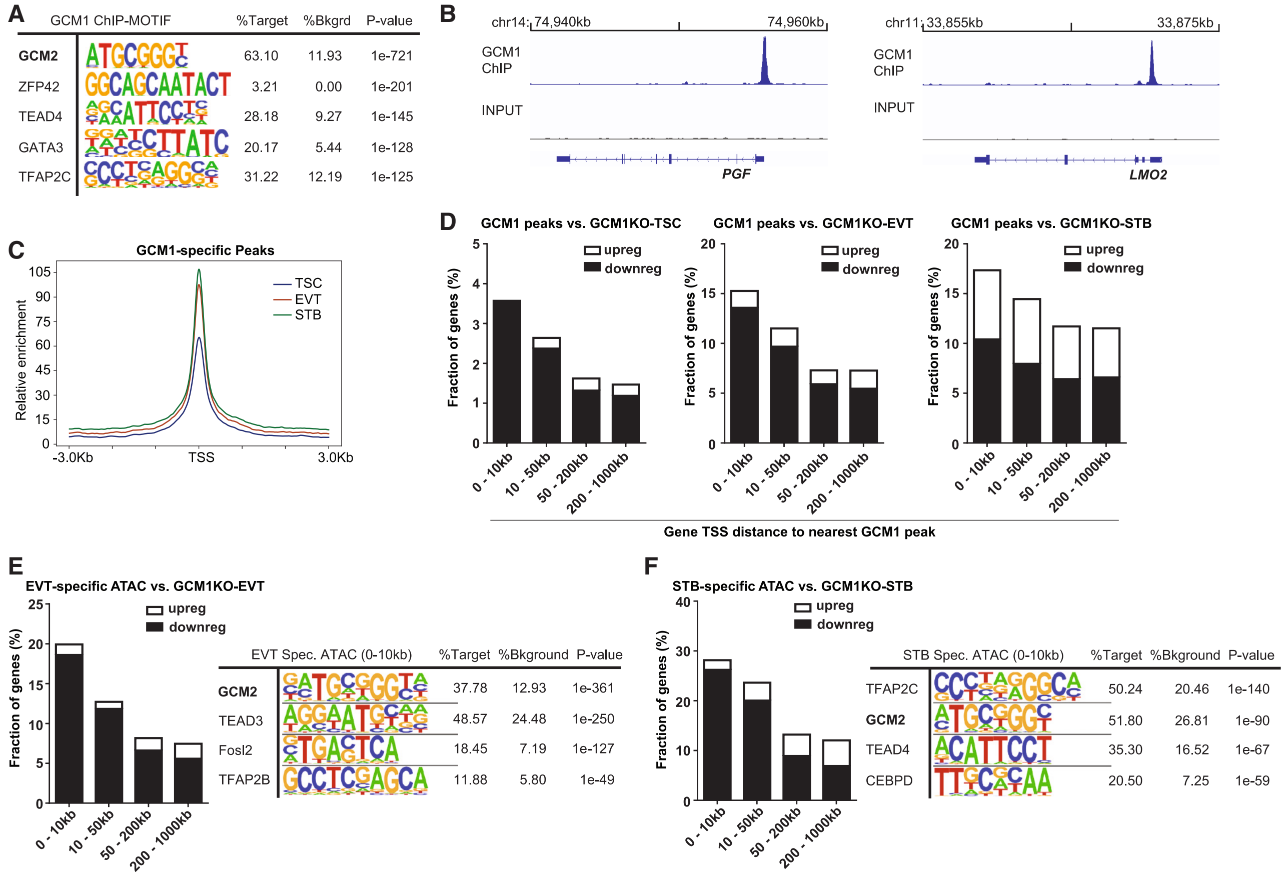

Figure 3. GCM1 positively regulates differentiation-associated genes

(A) Motif analysis of GCM1-binding sites shows very strong enrichment for GCM motif, indicating successful and specific ChIP.

(B) GCM1 enrichment over *PGF* (left) and *LMO2* (right).

(C) ATAC-seq enrichment in TSC, EVT, and STB over GCM1-binding sites.

(D) Plot showing the percentage of genes whose promoters are within a given distance of a GCM1-binding site that show upregulation or downregulation in $GCM1^{-/-}$ cells.

(E) Plot showing the percentage of genes whose promoters are within a given distance of an EVT-specific ATAC-seq site that show upregulation or downregulation in $GCM1^{-/-}$ cells (left), motif analysis for EVT-specific peaks (right).

(F) Plot showing the percentage of genes whose promoters are within a given distance of an STB-specific ATAC-seq site that show upregulation or downregulation in $GCM1^{-/-}$ cells (left), motif analysis for STB-specific peaks (right).

cavities, though not to the same extent as $GCM1^{-/-}$ (Figure S2P). TB-ORG generated from primary CTBs and treated with LY294002 at standard steady-state conditions with Matrigel showed reduced expression of the STB markers *CGB* and *ENDOU* (Figure 2N) and a modest reduction in *ITGA1* expression upon EVT differentiation while HLA-G levels were not affected (Figure 2O), suggesting that chemical modulation of GCM1 level hampers certain steps of STB and EVT differentiation.

GCM1 positively regulates EVT and STB-specific regulators

We performed chromatin immunoprecipitation (ChIP) sequencing (ChIP-seq) for GCM1 from day 3 EVTs (CT3), a time point which we found was conducive to high-quality ChIP data. We identified 2,271 peaks with >4-fold enrichment over input. Motif analysis of these sites showed extremely strong enrichment for the GCM-binding motif, with weaker enrichment for other TFs common in trophoblast (Figure 3A). We confirmed enrichment at known GCM1 targets such as *PGF* and *LMO2* (Chen et al., 2022; Jeyarajah et al., 2022; Li and Roberson, 2017) (Figure 3B). We also conducted an assay for transposase-accessible chromatin (ATAC-seq) to measure open chromatin regions in TSC, EVT, and STB, which accorded well with published H3K27Ac ChIP-seq data from these cells (Figure S3A). Comparison with the ATAC-seq data showed that GCM1 peaks corresponded to regions that show higher openness in EVT

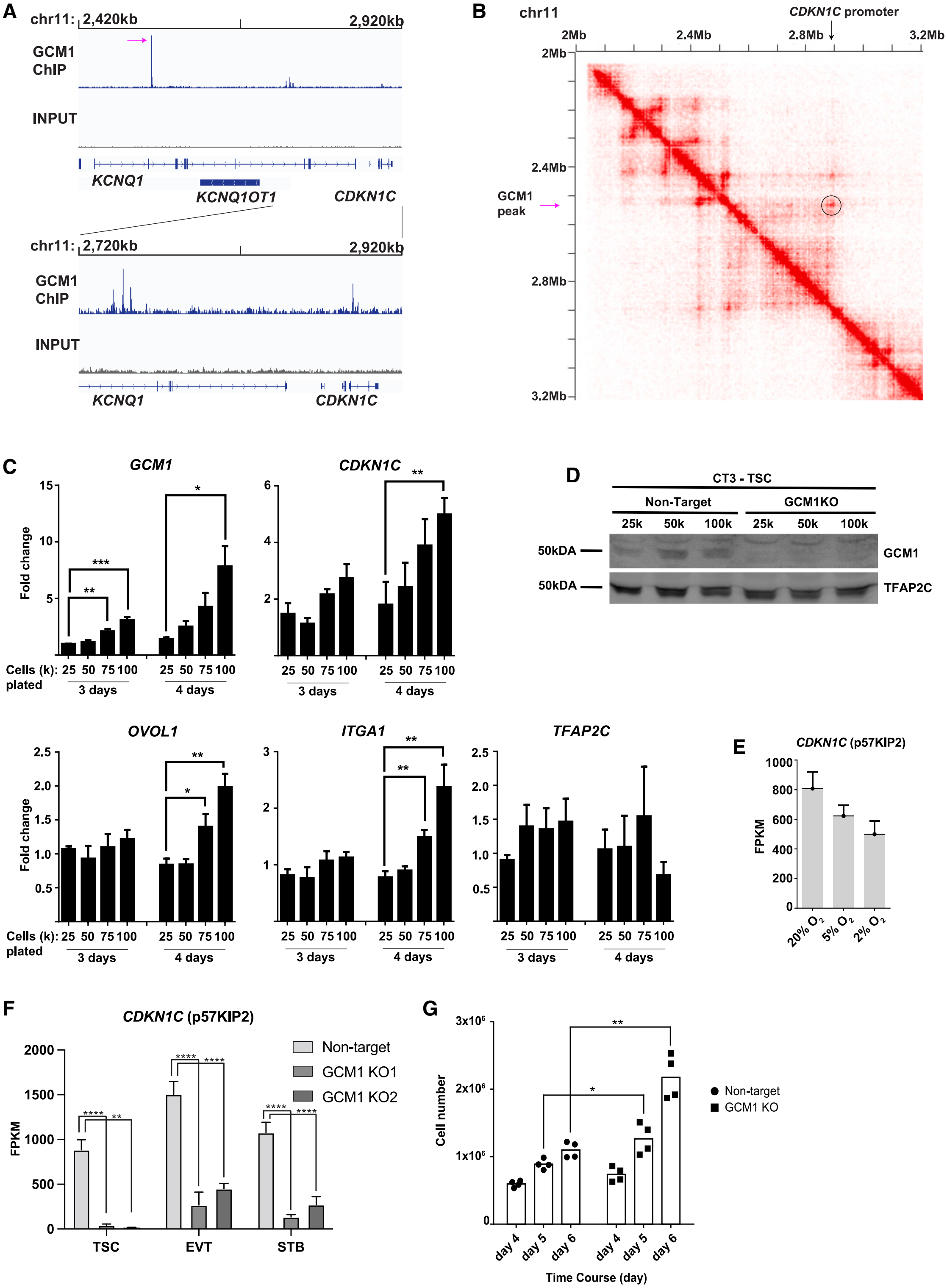

Figure 4. GCM1 positively regulates *CDKN1C* and contact inhibition

(A) GCM1 enrichment over imprinted *KCNQ* locus.

(B) Hi-C interaction data over the *GCM1* locus. Note physical association between GCM1-binding site and *CDKN1C* promoter. In (A) and (B), the highest GCM1 peak is indicated with a magenta arrow.

(legend continued on next page)

and STB than in TSC (Figures 3C and S3B; Table S4). Furthermore, we used region-associated differentially expressed gene analysis (Guo et al., 2021) to correlate the proximity of a GCM1 peak to the transcription start site (TSS) of genes dysregulated upon GCM1 knockout. Genes downregulated in *GCM1*$^{-/-}$ cells were found in proximity to GCM1 ChIP-seq peaks (Figure 3D). Putative direct targets, genes proximal to GCM1 peaks and downregulated in *GCM1*$^{-/-}$ cells, include known placenta differentiation factors such as *PGF*, *LMO2*, *CGA*, *OVOL1*, *SYDE1*, and a range of *CGB* genes (Figure S3C; Table S4).

Further supporting the role of GCM1 in regulating differentiation, ATAC-seq analysis of TSC, EVT, and STB data showed enrichment of the GCM motif in EVT and STB-specific regions of open chromatin (Figures 3E, 3F, S3D, and S3E). We also observed the association of EVT and STB-specific ATAC-seq peaks with genes downregulated in *GCM1*$^{-/-}$ cells of the corresponding cell types (Figures 3E and 3F).

GCM1 regulates CDKN1C and trophoblast overgrowth

One of the strongest GCM1 enrichment sites in the genome was found within the 11p15.5 imprinted locus (Smilinich et al., 1999), as were several smaller peaks (Figure 4A). This locus includes the transcripts *KCNQ1* and *KCNQ1OT1* and the protein CDKN1C (p57^{KIP2}), which binds to cyclin/CDK and blocks cell division (Takahashi et al., 2019). Analysis of published Hi-C data (Varberg et al., 2023), which shows three-dimensional interaction of regions of chromatin, shows a high degree of interaction between the strong GCM1-binding site in *KCNQ1* and the promoter of *CDKN1C* (Figures 4A and 4B).

CDKN1C shows preferential expression from the maternal allele (Matsuoka et al., 1995). A pregnancy abnormality called a full hydatidiform mole arises from an androgenetic pregnancy in which all genetic material is of paternal origin, and thus placental cells in hydatidiform moles feature loss of *CDKN1C* as well as persistent trophoblastic outgrowth (Jun et al., 2003). hTSCs upregulate *CDKN1C* at high confluence, and *CDKN1C*$^{-/-}$ hTSCs lose contact inhibition and continue growing after reaching confluence (Takahashi et al., 2019).

When we grew hTSCs to high confluence, we observed upregulation of *GCM1* and *CDKN1C* in tandem (Figures 4C and 4D), with more modest increases in other differentiation markers (Figure 4C). We also observed lower *CDKN1C* expression in hypoxia (Figure 4E), where levels of GCM1 are lower (Figures 1K and S1L), though this drop in *CDKN1C* does not reach statistical significance. Further consistent with direct regulation by GCM1, *CDKN1C* was dramatically downregulated in *GCM1*$^{-/-}$ hTSCs and differentiated cells (Figure 4F). While control cells stopped dividing as confluence occurs, *GCM1*$^{-/-}$ hTSCs continued to expand, similar to the reported *CDKN1C*$^{-/-}$ phenotype (Figures 4G and S4) (Takahashi et al., 2019). These results collectively indicate that GCM1 acts upstream of *CDKN1C* in response to confluence and controls its expression.

DISCUSSION

Considering the positive effect of hypoxia on spongiotrophoblast differentiation in mice, how can we explain the generally inhibitory effect of low oxygen on EVT differentiation in humans? A critical difference between mice and humans, as illustrated in our work and others (Jeyarajah et al., 2022; Shimizu et al., 2023; Wang et al., 2022), is that GCM1 is essential for both EVT and STB formation in human but only STB formation in mouse. Hence, if hypoxia negatively regulates GCM1 in both mice and humans, this would be predicted to have an inhibitory effect on EVT differentiation in humans but not on spongiotrophoblast or trophoblast giant cell formation in mice. We also find, somewhat counterintuitively, that hypoxia has a positive effect on HLA-G expression even though it has an overall negative effect on EVT differentiation. It is worth noting here that hTSCs, while clearly bipotent, express some markers consistent with cell column CTB, indicating that they may be more EVT-like than typical villous CTBs (Cinkornpumin et al., 2020; Lee et al., 2018a; Shannon et al., 2024). Hence, the observation that hypoxia promotes proximal-column EVT transcriptional program (Wakeland et al., 2017) is not necessarily incompatible with a role in hTSC self-renewal.

(C) Expression of genes indicated in plating conditions (cell number and growth time) indicated. Note that plating at higher densities leads to higher expression of *GCM1* and *CDKN1C* (CT1 hTSCs, $n = 4$ replicates). Statistical significance was determined via a two-tailed t test.
(D) GCM1 protein levels increase with higher confluence.
(E) Expression of *CDKN1C* in oxygen concentration indicated.
(F) Expression of *CDKN1C* in control and *GCM1*$^{-/-}$ KO1 and KO2 hTSC and differentiated cells (significance marked by $p_{adj.}$ value from DESEQ2 analysis, see Table S3).
(G) Cell number after plating 50k cells and allowing cells to grow for indicated number of days. Note a leveling off in non-targeting cells as cell lines reach confluence, but continued growth in *GCM1*$^{-/-}$ hTSCs (CT3 hTSCs, $n = 4$ replicates, including $n = 2$ GCM1KO1 and $n = 2$ GCM1KO2). Statistical significance was determined via a two-tailed t-test.

With regard to pathology, preeclampsia is widely understood to feature inadequate remodeling of maternal arterioles and concomitant reduced blood and oxygen availability for the placenta (Zhou et al., 1997), and higher levels of HIF-1α and/or HIF-2α protein have been observed in preeclamptic placenta (Soares et al., 2017). Excess of undifferentiated CTBs, which our study predicts would result from hypoxia, has also been reported (Redline and Patterson, 1995).

TSC organoids derived from human placenta collected over several gestational stages have the consistent feature that STB differentiation occurs inside the organoid (Haider et al., 2018; Sheridan et al., 2021; Turco et al., 2018; Yang et al., 2022). This may reflect higher pressure inside the organoid, or lack of access to Epidermal growth factor (EGF), but this organization is inverted with respect to the bilayer formation of chorionic villus (Enders and Blankenship, 1999). Two groups have succeeded in finding conditions in which STBs form on the outside of the organoid, though the resulting organoids cannot be propagated (Hori et al., 2024; Yang et al., 2024). We reasoned that reduction of *GCM1* level, as reported in literature from the PI3K inhibitor treatment, could allow sustained culture of undifferentiated organoids. We did observe reduced, though not eliminated, spontaneous differentiation. Interestingly, the published mechanism by which LY294002 reduces GCM1 expression, via activation of GSK3β, which then phosphorylates and degrades GCM1 (Chiang et al., 2009), would not be expected to work in TSCM media conditions in which GSK3β is perpetually inhibited via treatment with CHIR99021. Hence, it is something of a mystery how LY294002 prevents spontaneous differentiation in the organoid model, and future research in this area could yield improved culture conditions.

There is extensive literature demonstrating a role for Wnt signaling in maintenance of CTBs and hTSCs, along with substantial evidence for a role for GCM1 in the suppression of Wnt signaling. Nuclear β-catenin, the output of the canonical Wnt pathway, is observed in villous CTBs but is lost upon subsequent differentiation (Haider et al., 2018). CHIR99021, a Wnt activator, is essential for hTSC maintenance, and its removal facilitates directed differentiation to downstream lineages (Okae et al., 2018). Likewise, removal of CHIR99021 from self-renewing TB-ORG conditions is sufficient to allow differentiation to EVT (Haider et al., 2018). Concordantly, knockdown of *GCM1* in hTSCs results in elevated levels of Wnt pathway signaling, leading to failed EVT differentiation (Jeyarajah et al., 2022). Our results are broadly consistent with these findings, and we observe Wnt pathway ontology terms upregulated in 2% O_2 conditions (Figure 1I). Cell column CTBs show low expression of both the Wnt receptor *WLS* and *GCM1* (Figure S2L). We note that $GCM1^{-/-}$ TB-ORG cultured without CHIR99021 to induce EVT differentiation expressed the cell column marker ITGB6, suggesting that this is the most differentiated state that can be attained by *GCM1*-deficient hTSCs.

In a CRISPR dropout screen of hTSCs, *GCM1* is a growth-restricting gene, whose deletion promotes cell growth (Dong et al., 2022). This is likely due to two mechanisms. As shown earlier, hTSCs, especially at high confluence, undergo some spontaneous differentiation, which is suppressed by loss of GCM1. Furthermore, $GCM1^{-/-}$ hTSCs show greatly reduced *CDKN1C* expression, limiting contact inhibition. Indeed, *CDKN1C* is also a growth-restricting gene in CRISPR screens (Dong et al., 2022; Shimizu et al., 2023). Interestingly, despite its growth-restricting properties in culture, *GCM1* is not a known tumor suppressor in gestational choriocarcinoma (GC) (Fisher and Maher, 2021; Jung et al., 2020; Mello et al., 2017). In the case of hydatidiform-mole-derived GC, *CDKN1C* expression is already lost, but GC can also arise from non-molar pregnancy, and there is no evidence of GCM1 mutation in these diseases either. This may be because while loss of *GCM1* causes loss of contact inhibition and uncontrolled growth, it also precludes epithelial-mesenchymal transition (EMT) and invasiveness. At the same time, analysis of mutations and karyotypic abnormalities in GC remains limited, and there is almost nothing known about the mutational profile of the related placental cancers, placental site trophoblastic tumor, and epithelioid trophoblastic tumor (Hui et al., 2004; Oliver et al., 2021; Xu et al., 2009). New roles for GCM1 in placental development and placental cancer may have yet to be discovered.

METHODS

Cell culture maintenance and differentiation of hTSC

CT1, CT3, BT1, and BT2 hTSC lines were generously provided from Dr. Arima's lab in Japan with STR authentication. CT1 and CT3 were derived from first trimester placenta, and BT1 and BT2 hTSC were derived from blastocysts (Okae et al., 2018). Cell culture, including thawing, freezing and differentiating cells was performed according to published protocol (Okae et al., 2018). A detailed description of the maintenance and differentiation produces is included in the supplemental methods.

All cells were free of visible contamination and routinely tested negative for mycoplasma.

Cell culture—Standard maintenance and EVT differentiation of TB-ORGs

TB-ORGs were generated and cultured according to a recent publication (Haider et al., 2022). A detailed description is available in the supplemental methods .

TB-ORG and EVT differentiation (modified protocol without Matrigel)

3D TSCs (TB-ORG) of control and *GCM1*$^{-/-}$ cells were cultured based on a modification from the Okae et al. (2018) and Haider et al. (2022) publications. Our modified trophoblast organoid media (mTOM) contains DMEM F-12 (Gibco), 10 mM HEPES (Gibco), 1× ITS-X (Gibco), 2 mM GlutaMax (Gibco), 1× Penicillin/Streptomycin (Gibco), 0.2% ESC-FBS (Gibco), 50 ng/mL rhEGF (Invitrogen), 3 μM CHIR99021 (Cayman Chemical), 2 μM A8301 (Cayman Chemical), and 5 μM Y27632 (Cayman Chemical). Instead of Matrigel embedding, micro-V-shaped wells (AggreWell 400, STEMCELL Technologies) were used to generate organoids. ~50,000 cells were resuspended in 1 mL mTOM per 24 well and centrifuged in a plate spinner for 5 min at 1,000 rpm to collect cells to the bottom at ~100 cells per V-shaped well. After 24 h, 500 μL of media is gently removed and either mTOM is replaced or mTOM without CHIR99021 (mTOM-C) for CTB-CCC/precursor to EVT formation was replaced every other day over a ~10-day period. For LY294002 treatment, treatment up to 14 days may be required to physically observe cavity formation by microscopy.

Generation of CRISPR knockout lines by nucleofection

CRISPR guides were designed using IDT Custom Alt-R CRISPR-Cas9 guide RNA program. sgRNA guides targeting a small region of nucleotide just after the ATG start site in exon 2 and introns 2–3 (sgRNA1: UCU UCA GAA UCA AAG UCG UC and sgRNA2: ACU AUU AAC AUG CGG AGA CC). Additional deletion of exon 3 was designed (sgRNA3: GAG CGC UGC UCA GAU AGC GA and sgRNA4: AGA CCU AAG AGC AAU CAG UG). Cas9-sgRNA ribonucleoprotein complexes were generated from sgRNA and Cas9 protein (Synthego). In brief, 75 pmol of each individual sgRNA is complexed with 10 pmol of Cas9 protein in Cas9 annealing buffer (NEB) for 10 min. In the meantime, TSCs are dissociated with 30% TrypLE and reconstituted in PBS to a concentration of 1×10^5 cell/μL. Pre-complexed ribonucleoproteins sgRNA1 and sgRNA2 are combined together with 10 μL of cell suspension and 20 μL of P3 solution (Lonza) and transferred to a cuvette to be nucleofected using an Amaxa 4D nucleofector (Lonza) with pulse code CA137. Immediately after, 150 μL of TSC media was added to the cuvette to neutralize the reaction, and cells were transferred to a freshly prepared 10 cm plate coated with Lam-511 and TSCM for generating single clones. 2 mg/mL Collagenase V solution was used to dissociate colonies in order to pick single clones. Deletions were confirmed by genotyping (GCM1KO1: forward, TTGTATGAGGACTTGTGCATAACAA and reverse, GCCATTGGTTACAGATGACAAC; GCM1KO2: forward, ATGGAACTCACAGGGGCTAT and reverse, TAAC AGGAGCCTTCAGTCCA).

hCG ELISA

Human chorionic gonadatropin (hCG) secretion was measured using an hCG AccuBind ELISA (Monobind) according to manufacturer's instructions.

Confluence experiment

CT1 and CT3 hTSCs were plated in 24-well plates at 4 different densities (25, 50, 75, and 100K cells), in duplicates, and incubated for 48 or 72 h. Cells were collected using TrypLE 30% at 37°C for 10 min followed by addition of trypsin inhibitor. Single cells obtained were centrifuged to obtain a pellet, washed with PBS, and flash frozen and stored at −80°C.

RNA isolation and qPCR

A detailed description of RNA isolation and qPCR, including a table of primers used, is included in the supplemental methods section.

Next-generation sequencing libraries

ChIP was performed as previously described (Jeyarajah et al., 2022). ATAC-seq libraries were generated using a commercially available kit Active Motif (#53150, Carlsbad, CA). RNA-seq libraries were generated using an NEBNext Ultra RNA Library Prep Kit.

See supplemental methods for detailed descriptions of library generation.

Sequencing analysis

For RNA-seq and ChIP/ATAC-seq analysis, Genpipes 4.3.2 (https://bitbucket.org/mugqic/genpipes) provided the pipeline for basic sequencing processing. For RNA-seq, sequencing quality and adaptor removal were trimmed with trimmomatic (v.0.36). Trimmed fastq file alignment was performed with STAR aligner (v.2.7.8a) using hg38/GRCh38 (ensembl v.104). Picard (v.2.9.0) was then used to merge, mark duplicates, identify unique read, and sort .bam files proceeding alignment. Read counts were collected using HTseq-count and StringTie (v.1.3.5). Differentially expressed gene comparison was performed with DESeq2 package on RStudio (R v.4.3.1). Correlation matrix, hierarchal gene cluster analysis, and PCA were generated in R. Gene pathway analysis was performed using ConsensusPathwayDB and EnrichR. For ChIP-seq and ATAC-seq, in brief, raw fastq files were trimmed using trimmomatic (v.0.36). Then, qualified fastq reads were mapped with BWA (v.0.7.17) with post processing with sambamda (v.0.8.1) to merge replicates, mark and filter duplicates, and remove blacklist regions. Peak calling and differential bind were performed with MACS2 (v.2.2.7.1). Gene annotations and motif analysis were performed with Homer (v.4.11). Bam to bigwig bamCoverage from deepTools (v.3.5.1) was used to generate the tracks for viewing on IGV (v.2.9.4).

Ethics

All research was approved by the McGill Faculty of Medicine and Health Sciences Research Ethics Board (McGill IRB).

RESOURCE AVAILABILITY

Lead contact

Requests for further information or cell lines will be fulfilled by the lead contact, William A. Pastor (william.pastor@mcgill.ca).

Materials availability

Cell lines generated in the course of this study will be made available upon reasonable requests.

Data and code availability

Sequencing data were deposited to the Gene Expression Omnibus (GEO) repository with the following accession numbers: RNA-seq (GSE276594 and GSE276595), ATAC-seq (GSE276588), and ChIP-seq (GSE276590). Passage number is indicated in GEO submission.

ACKNOWLEDGMENTS

We thank the Rosalind & Morris Goodman Cancer Institute Flow Cytometry core, the SickKids Center for Applied Genomics facility, the La Jolla Institute for Allergy and Immunology Sequencing Core, and the Canada Michael Smith Genome Sciences Center at BC Cancer for their dedicated service. We thank Dr. Brian Cox (University of Toronto) for sharing code to perform single-cell RNA-seq analysis. This work was funded by the New Frontiers in Research Fund (NFRF) grant NFRFE-2018-00883 and the Canadian Institutes of Health Research (CIHR) project grant PJT-166169 to W.A.P., the NIH grants HD101319, HD062546, and HD103161 to S.P., the NSERC Discovery Grant RGPIN-2016-05053 and CIHR project grant PJT-180483 to S.J.R. and the Austrian Science Fund P34588-B and P36159-B to S.H. W.A.P. was supported by an FRQS Chercheurs-boursier. J.K.C. was supported by a Fonds de recherche Santé Québec graduate fellowship and studentships from the McGill University Faculty of Medicine.

AUTHOR CONTRIBUTIONS

J.K.C., S.Y.K., A.-M.P., T.M., J.S., J.G., J.Z., P.D., and M.J.J. conducted the experiments. J.K.C. and D.S. conducted the bioinformatic analysis. S.J.R., S.P., S.H., and W.A.P. supervised the experiments and analysis.

DECLARATION OF INTERESTS

The authors declare no competing interests.

SUPPLEMENTAL INFORMATION

Supplemental information can be found online at https://doi.org/10.1016/j.stemcr.2025.102481.

Received: October 21, 2024
Revised: March 26, 2025
Accepted: March 27, 2025
Published: April 24, 2025

REFERENCES

Alsat, E., Wyplosz, P., Malassiné, A., Guibourdenche, J., Porquet, D., Nessmann, C., and Evain-Brion, D. (1996). Hypoxia impairs cell fusion and differentiation process in human cytotrophoblast, in vitro. J. Cell. Physiol. *168*, 346–353. https://doi.org/10.1002/(SICI)1097-4652(199608)168:2<346::AID-JCP13>3.0.CO;2-1.

Arutyunyan, A., Roberts, K., Troulé, K., Wong, F.C.K., Sheridan, M.A., Kats, I., Garcia-Alonso, L., Velten, B., Hoo, R., Ruiz-Morales, E.R., et al. (2023). Spatial multiomics map of trophoblast development in early pregnancy. Nature *616*, 143–151. https://doi.org/10.1038/s41586-023-05869-0.

Chang, C.W., Wakeland, A.K., and Parast, M.M. (2018). Trophoblast lineage specification, differentiation and their regulation by oxygen tension. J. Endocrinol. *236*, R43–R56. https://doi.org/10.1530/JOE-17-0402.

Chazaud, C., and Yamanaka, Y. (2016). Lineage specification in the mouse preimplantation embryo. Development *143*, 1063–1074. https://doi.org/10.1242/dev.128314.

Chen, Y., Meng, Y., Yu, Y., Li, W., Shen, Y., Li, S., Chang, Y., and Sun, W. (2022). LMO2 plays differential roles in trophoblast subtypes and is associated with preeclampsia. Biochem. Biophys. Res. Commun. *604*, 43–50. https://doi.org/10.1016/j.bbrc.2022.03.033.

Chiang, M.H., Liang, F.Y., Chen, C.P., Chang, C.W., Cheong, M.L., Wang, L.J., Liang, C.Y., Lin, F.Y., Chou, C.C., and Chen, H. (2009). Mechanism of hypoxia-induced GCM1 degradation: implications for the pathogenesis of preeclampsia. J. Biol. Chem. *284*, 17411–17419. https://doi.org/10.1074/jbc.M109.016170.

Chiu, Y.H., and Chen, H. (2016). GATA3 inhibits GCM1 activity and trophoblast cell invasion. Sci. Rep. *6*, 21630. https://doi.org/10.1038/srep21630.

Cinkornpumin, J.K., Kwon, S.Y., Guo, Y., Hossain, I., Sirois, J., Russett, C.S., Tseng, H.W., Okae, H., Arima, T., Duchaine, T.F., et al. (2020). Naive Human Embryonic Stem Cells Can Give Rise to Cells with a Trophoblast-like Transcriptome and Methylome. Stem Cell Rep. *15*, 198–213. https://doi.org/10.1016/j.stemcr.2020.06.003.

Cowden Dahl, K.D., Fryer, B.H., Mack, F.A., Compernolle, V., Maltepe, E., Adelman, D.M., Carmeliet, P., and Simon, M.C. (2005). Hypoxia-inducible factors 1alpha and 2alpha regulate trophoblast differentiation. Mol. Cell Biol. *25*, 10479–10491. https://doi.org/10.1128/MCB.25.23.10479-10491.2005.

Dong, C., Fu, S., Karvas, R.M., Chew, B., Fischer, L.A., Xing, X., Harrison, J.K., Popli, P., Kommagani, R., Wang, T., et al. (2022). A genome-wide CRISPR-Cas9 knockout screen identifies essential and growth-restricting genes in human trophoblast stem cells. Nat. Commun. *13*, 2548. https://doi.org/10.1038/s41467-022-30207-9.

Enders, A.C., and Blankenship, T.N. (1999). Comparative placental structure. Adv. Drug Deliv. Rev. *38*, 3–15. https://doi.org/10.1016/s0169-409x(99)00003-4.

Fisher, R.A., and Maher, G.J. (2021). Genetics of gestational trophoblastic disease. Best Pract. Res. Clin. Obstet. Gynaecol. *74*, 29–41. https://doi.org/10.1016/j.bpobgyn.2021.01.004.

Genbacev, O., Joslin, R., Damsky, C.H., Polliotti, B.M., and Fisher, S.J. (1996). Hypoxia alters early gestation human cytotrophoblast differentiation/invasion in vitro and models the placental defects

that occur in preeclampsia. J. Clin. Investig. *97*, 540–550. https://doi.org/10.1172/JCI118447.

Genbacev, O., Zhou, Y., Ludlow, J.W., and Fisher, S.J. (1997). Regulation of human placental development by oxygen tension. Science *277*, 1669–1672.

Gnarra, J.R., Ward, J.M., Porter, F.D., Wagner, J.R., Devor, D.E., Grinberg, A., Emmert-Buck, M.R., Westphal, H., Klausner, R.D., and Linehan, W.M. (1997). Defective placental vasculogenesis causes embryonic lethality in VHL-deficient mice. Proc. Natl. Acad. Sci. USA *94*, 9102–9107. https://doi.org/10.1073/pnas.94.17.9102.

Guo, Y., Xue, Z., Yuan, R., Li, J.J., Pastor, W.A., and Liu, W. (2021). RAD: a web application to identify region associated differentially expressed genes. Bioinformatics *37*, 2741–2743. https://doi.org/10.1093/bioinformatics/btab075.

Haider, S., Lackner, A.I., Dietrich, B., Kunihs, V., Haslinger, P., Meinhardt, G., Maxian, T., Saleh, L., Fiala, C., Pollheimer, J., et al. (2022). Transforming growth factor-beta signaling governs the differentiation program of extravillous trophoblasts in the developing human placenta. Proc. Natl. Acad. Sci. USA *119*, e2120667119. https://doi.org/10.1073/pnas.2120667119.

Haider, S., Meinhardt, G., Saleh, L., Kunihs, V., Gamperl, M., Kaindl, U., Ellinger, A., Burkard, T.R., Fiala, C., Pollheimer, J., et al. (2018). Self-Renewing Trophoblast Organoids Recapitulate the Developmental Program of the Early Human Placenta. Stem Cell Rep. *11*, 537–551. https://doi.org/10.1016/j.stemcr.2018.07.004.

Hori, T., Okae, H., Shibata, S., Kobayashi, N., Kobayashi, E.H., Oike, A., Sekiya, A., Arima, T., and Kaji, H. (2024). Trophoblast stem cell-based organoid models of the human placental barrier. Nat. Commun. *15*, 962. https://doi.org/10.1038/s41467-024-45279-y.

Hui, P., Riba, A., Pejovic, T., Johnson, T., Baergen, R.N., and Ward, D. (2004). Comparative genomic hybridization study of placental site trophoblastic tumour: a report of four cases. Mod. Pathol. *17*, 248–251. https://doi.org/10.1038/modpathol.3800025.

Jaremek, A., Shaha, S., Jeyarajah, M.J., Jaju Bhattad, G., Chowdhury, D., Riddell, M., and Renaud, S.J. (2023). Genome-Wide Analysis of Hypoxia-Inducible Factor Binding Reveals Targets Implicated in Impaired Human Placental Syncytiotrophoblast Formation under Low Oxygen. Am. J. Pathol. *193*, 846–865. https://doi.org/10.1016/j.ajpath.2023.03.006.

Jauniaux, E., Watson, A., and Burton, G. (2001). Evaluation of respiratory gases and acid-base gradients in human fetal fluids and uteroplacental tissue between 7 and 16 weeks' gestation. Am. J. Obstet. Gynecol. *184*, 998–1003. https://doi.org/10.1067/mob.2001.111935.

Jeyarajah, M.J., Jaju Bhattad, G., Kelly, R.D., Baines, K.J., Jaremek, A., Yang, F.H.P., Okae, H., Arima, T., Dumeaux, V., and Renaud, S.J. (2022). The multifaceted role of GCM1 during trophoblast differentiation in the human placenta. Proc. Natl. Acad. Sci. USA *119*, e2203071119. https://doi.org/10.1073/pnas.2203071119.

Jun, S.Y., Ro, J.Y., and Kim, K.R. (2003). p57kip2 is useful in the classification and differential diagnosis of complete and partial hydatidiform moles. Histopathology *43*, 17–25. https://doi.org/10.1046/j.1365-2559.2003.01667.x.

Jung, S.H., Choi, Y.J., Kim, M.S., Park, H.C., Han, M.R., Hur, S.Y., Lee, A.W., Shin, O.R., Kim, J., Lee, S.H., et al. (2020). Distinct genomic profiles of gestational choriocarcinoma, a unique cancer of pregnant tissues. Exp. Mol. Med. *52*, 2046–2054. https://doi.org/10.1038/s12276-020-00544-0.

Kozak, K.R., Abbott, B., and Hankinson, O. (1997). ARNT-deficient mice and placental differentiation. Dev. Biol. *191*, 297–305. https://doi.org/10.1006/dbio.1997.8758.

Lee, C.Q.E., Turco, M.Y., Gardner, L., Simons, B.D., Hemberger, M., and Moffett, A. (2018a). Integrin alpha2 marks a niche of trophoblast progenitor cells in first trimester human placenta. Development *145*, dev162305. https://doi.org/10.1242/dev.162305.

Lee, C.Q.E., Turco, M.Y., Gardner, L., Simons, B.D., Hemberger, M., and Moffett, A. (2018b). Integrin α2 marks a niche of trophoblast progenitor cells in first trimester human placenta. Development *145*, dev162305. https://doi.org/10.1242/dev.162305.

Li, S., and Roberson, M.S. (2017). Dlx3 and GCM-1 functionally coordinate the regulation of placental growth factor in human trophoblast-derived cells. J. Cell. Physiol. *232*, 2900–2914. https://doi.org/10.1002/jcp.25752.

Majmundar, A.J., Wong, W.J., and Simon, M.C. (2010). Hypoxia-inducible factors and the response to hypoxic stress. Mol. Cell *40*, 294–309. https://doi.org/10.1016/j.molcel.2010.09.022.

Matsuoka, S., Edwards, M.C., Bai, C., Parker, S., Zhang, P., Baldini, A., Harper, J.W., and Elledge, S.J. (1995). p57KIP2, a structurally distinct member of the p21CIP1 Cdk inhibitor family, is a candidate tumor suppressor gene. Genes Dev. *9*, 650–662. https://doi.org/10.1101/gad.9.6.650.

Mello, J.B.H.d., Ramos Cirilo, P.D., Michelin, O.C., Custódio Domingues, M.A., Cunha Rudge, M.V., Rogatto, S.R., and Maestá, I. (2017). Genomic profile in gestational and non-gestational choriocarcinomas. Placenta *50*, 8–15. https://doi.org/10.1016/j.placenta.2016.12.009.

Natsuizaka, M., Naganuma, S., Kagawa, S., Ohashi, S., Ahmadi, A., Subramanian, H., Chang, S., Nakagawa, K.J., Ji, X., Liebhaber, S.A., et al. (2012). Hypoxia induces IGFBP3 in esophageal squamous cancer cells through HIF-1alpha-mediated mRNA transcription and continuous protein synthesis. FASEB J. *26*, 2620–2630. https://doi.org/10.1096/fj.11-198598.

Nelson, D.M., Johnson, R.D., Smith, S.D., Anteby, E.Y., and Sadovsky, Y. (1999). Hypoxia limits differentiation and up-regulates expression and activity of prostaglandin H synthase 2 in cultured trophoblast from term human placenta. Am. J. Obstet. Gynecol. *180*, 896–902. https://doi.org/10.1016/s0002-9378(99)70661-7.

Okae, H., Toh, H., Sato, T., Hiura, H., Takahashi, S., Shirane, K., Kabayama, Y., Suyama, M., Sasaki, H., and Arima, T. (2018). Derivation of Human Trophoblast Stem Cells. Cell Stem Cell *22*, 50–63.e6. https://doi.org/10.1016/j.stem.2017.11.004.

Oliver, G.R., Marcano-Bonilla, S., Quist, J., Tolosa, E.J., Iguchi, E., Swanson, A.A., Hoppman, N.L., Schwab, T., Sigafoos, A., Prodduturi, N., et al. (2021). LPCAT1-TERT fusions are uniquely recurrent in epithelioid trophoblastic tumors and positively regulate cell growth. PLoS One *16*, e0250518. https://doi.org/10.1371/journal.pone.0250518.

Redline, R.W., and Patterson, P. (1995). Pre-eclampsia is associated with an excess of proliferative immature intermediate trophoblast. Hum. Pathol. *26*, 594–600. https://doi.org/10.1016/0046-8177(95)90162-0.

Rodesch, F., Simon, P., Donner, C., and Jauniaux, E. (1992). Oxygen measurements in endometrial and trophoblastic tissues during early pregnancy. Obstet. Gynecol. *80*, 283–285.

Sato, Y. (2020). Endovascular trophoblast and spiral artery remodeling. Mol. Cell. Endocrinol. *503*, 110699. https://doi.org/10.1016/j.mce.2019.110699.

Shannon, M.J., McNeill, G.L., Koksal, B., Baltayeva, J., Wächter, J., Castellana, B., Peñaherrera, M.S., Robinson, W.P., Leung, P.C.K., and Beristain, A.G. (2024). Single-cell assessment of primary and stem cell-derived human trophoblast organoids as placenta-modeling platforms. Dev. Cell *59*, 776–792.e11. https://doi.org/10.1016/j.devcel.2024.01.023.

Sheridan, M.A., Zhao, X., Fernando, R.C., Gardner, L., Perez-Garcia, V., Li, Q., Marsh, S.G.E., Hamilton, R., Moffett, A., and Turco, M.Y. (2021). Characterization of primary models of human trophoblast. Development *148*, dev199749. https://doi.org/10.1242/dev.199749.

Sherman, B.T., Hao, M., Qiu, J., Jiao, X., Baseler, M.W., Lane, H.C., Imamichi, T., and Chang, W. (2022). DAVID: a web server for functional enrichment analysis and functional annotation of gene lists (2021 update). Nucleic Acids Res. *50*, W216–W221. https://doi.org/10.1093/nar/gkac194.

Shimizu, T., Oike, A., Kobayashi, E.H., Sekiya, A., Kobayashi, N., Shibata, S., Hamada, H., Saito, M., Yaegashi, N., Suyama, M., et al. (2023). CRISPR screening in human trophoblast stem cells reveals both shared and distinct aspects of human and mouse placental development. Proc. Natl. Acad. Sci. USA *120*, e2311372120. https://doi.org/10.1073/pnas.2311372120.

Smilinich, N.J., Day, C.D., Fitzpatrick, G.V., Caldwell, G.M., Lossie, A.C., Cooper, P.R., Smallwood, A.C., Joyce, J.A., Schofield, P.N., Reik, W., et al. (1999). A maternally methylated CpG island in KvLQT1 is associated with an antisense paternal transcript and loss of imprinting in Beckwith-Wiedemann syndrome. Proc. Natl. Acad. Sci. USA *96*, 8064–8069. https://doi.org/10.1073/pnas.96.14.8064.

Soares, M.J., Iqbal, K., and Kozai, K. (2017). Hypoxia and Placental Development. Birth Defects Res. *109*, 1309–1329. https://doi.org/10.1002/bdr2.1135.

Takahashi, S., Okae, H., Kobayashi, N., Kitamura, A., Kumada, K., Yaegashi, N., and Arima, T. (2019). Loss of p57(KIP2) expression confers resistance to contact inhibition in human androgenetic trophoblast stem cells. Proc. Natl. Acad. Sci. USA *116*, 26606–26613. https://doi.org/10.1073/pnas.1916019116.

Takeda, K., Ho, V.C., Takeda, H., Duan, L.J., Nagy, A., and Fong, G.H. (2006). Placental but not heart defects are associated with elevated hypoxia-inducible factor alpha levels in mice lacking prolyl hydroxylase domain protein 2. Mol. Cell Biol. *26*, 8336–8346. https://doi.org/10.1128/MCB.00425-06.

Turco, M.Y., Gardner, L., Kay, R.G., Hamilton, R.S., Prater, M., Hollinshead, M.S., McWhinnie, A., Esposito, L., Fernando, R., Skelton, H., et al. (2018). Trophoblast organoids as a model for maternal-fetal interactions during human placentation. Nature *564*, 263–267. https://doi.org/10.1038/s41586-018-0753-3.

Varberg, K.M., Dominguez, E.M., Koseva, B., Varberg, J.M., McNally, R.P., Moreno-Irusta, A., Wesley, E.R., Iqbal, K., Cheung, W.A., Schwendinger-Schreck, C., et al. (2023). Extravillous trophoblast cell lineage development is associated with active remodeling of the chromatin landscape. Nat. Commun. *14*, 4826. https://doi.org/10.1038/s41467-023-40424-5.

Wakeland, A.K., Soncin, F., Moretto-Zita, M., Chang, C.W., Horii, M., Pizzo, D., Nelson, K.K., Laurent, L.C., and Parast, M.M. (2017). Hypoxia Directs Human Extravillous Trophoblast Differentiation in a Hypoxia-Inducible Factor-Dependent Manner. Am. J. Pathol. *187*, 767–780. https://doi.org/10.1016/j.ajpath.2016.11.018.

Wang, L.J., Chen, C.P., Lee, Y.S., Ng, P.S., Chang, G.D., Pao, Y.H., Lo, H.F., Peng, C.H., Cheong, M.L., and Chen, H. (2022). Functional antagonism between DeltaNp63alpha and GCM1 regulates human trophoblast stemness and differentiation. Nat. Commun. *13*, 1626. https://doi.org/10.1038/s41467-022-29312-6.

Xu, M.L., Yang, B., Carcangiu, M.L., and Hui, P. (2009). Epithelioid trophoblastic tumor: comparative genomic hybridization and diagnostic DNA genotyping. Mod. Pathol. *22*, 232–238. https://doi.org/10.1038/modpathol.2008.165.

Yang, L., Liang, P., Yang, H., and Coyne, C.B. (2024). Trophoblast organoids with physiological polarity model placental structure and function. J. Cell Sci. *137*, jcs261528. https://doi.org/10.1242/jcs.261528.

Yang, L., Semmes, E.C., Ovies, C., Megli, C., Permar, S., Gilner, J.B., and Coyne, C.B. (2022). Innate immune signaling in trophoblast and decidua organoids defines differential antiviral defenses at the maternal-fetal interface. Elife *11*, e79794. https://doi.org/10.7554/eLife.79794.

Zhou, Y., Damsky, C.H., and Fisher, S.J. (1997). Preeclampsia is associated with failure of human cytotrophoblasts to mimic a vascular adhesion phenotype. One cause of defective endovascular invasion in this syndrome? J. Clin. Investig. *99*, 2152–2164. https://doi.org/10.1172/JCI119388.

Stem Cell Reports, Volume *20*

Supplemental Information

Hypoxia and loss of *GCM1* expression prevent differentiation and contact inhibition in human trophoblast stem cells

Jessica K. Cinkornpumin, Sin Young Kwon, Anna-Maria Prandstetter, Theresa Maxian, Jacinthe Sirois, James Goldberg, Joy Zhang, Deepak Saini, Purbasa Dasgupta, Mariyan J. Jeyarajah, Stephen J. Renaud, Soumen Paul, Sandra Haider, and William A. Pastor

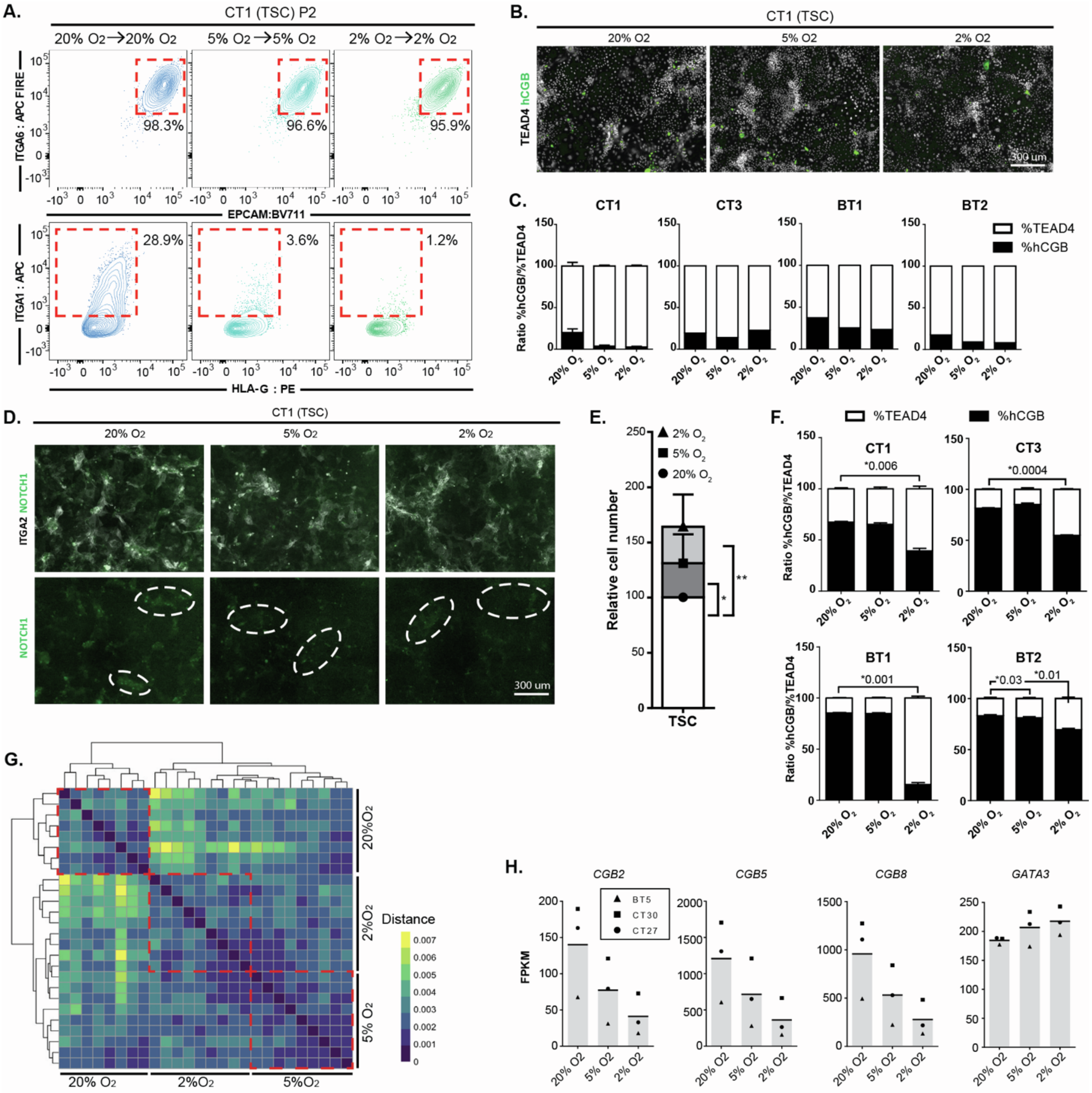

I. Enriched transcription factor target sets Cluster1

351 genes (13.8%) from the input list are present in at least one TF target set.
The total number of genes present in at least one TF target set and identifiable by 'ensembl' IDs is **2373**.

transcription factor	set size	candidates contained	p-value	q-value
HIF1A	82	30 (36.6%)	5.51e-07	0.000103
PTTG1	15	9 (60.0%)	6.74e-05	0.0063
MYCN	45	16 (35.6%)	0.000398	0.0248
MYC	99	25 (25.5%)	0.00316	0.129
VHL	20	8 (40.0%)	0.00523	0.129
HDGF	6	4 (66.7%)	0.00552	0.129
ARNTL	6	4 (66.7%)	0.00552	0.129
HIVEP2	6	4 (66.7%)	0.00552	0.129
CLOCK	13	6 (46.2%)	0.00686	0.143
TFDP1	10	5 (50.0%)	0.00914	0.171

J. Enriched transcription factor target sets Cluster2

338 genes (11.5%) from the input list are present in at least one TF target set.
The total number of genes present in at least one TF target set and identifiable by 'ensembl' IDs is **2373**.

transcription factor	set size	candidates contained	p-value	q-value
GCM1	4	4 (100.0%)	0.000405	0.0595
ATF4	35	13 (37.1%)	0.000601	0.0595
KLF6	20	8 (40.0%)	0.00411	0.217
HIC1	9	5 (55.6%)	0.00439	0.217
DDIT3	18	7 (38.9%)	0.00866	0.269
PPARG	66	17 (25.8%)	0.00878	0.269

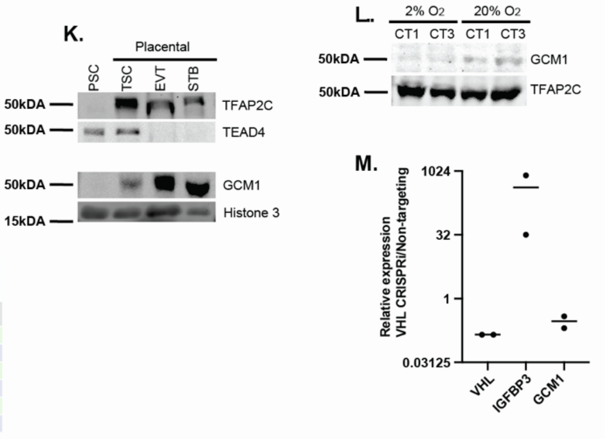

Figure S1 (related to Figure 1). Reduced and impaired hTSC differentiation in hypoxic conditions. A. Trophoblast stem cells shown in Figure. 1A were passaged and cultured for additional 72hrs in varying levels of oxygen (20%, 5%, 2% O_2). Continued low oxygen tension causes further reduction of ITGA1$^+$ cell population. **B.** Spontaneous differentiation of hTSC to STB in as indicated by loss of TEAD4 and gain of hCGB. Note trend toward higher hCGB staining at 20% O_2. **C.** Quantification of spontaneous hCGB expression across multiple cell lines at oxygen concentration indicated (4 cell lines, n=4 wells each cell line per condition). **D.** hTSC in the varying oxygen conditions were grown to over maximum confluency. At regions where overgrowth causes increase cell-to-cell contact and cell pile-up, spontaneous nuclear NOTCH1 signal is observed. In the low oxygen cultures, NOTCH1 expression is not detected. **E.** Relative numbers of TEAD4+ cells per unit area (4 cell lines, n=3 for each cell line over 3 different passages). **F.** Quantification of hCGB expression upon directed differentiation to STB in multiple cell lines at oxygen concentration indicated (4 cell lines, n=4 wells for each cell line per condition). **G.** Correlation matrix showing sample clustering of RNA-seq data from hTSCs in culture conditions indicated. **H.** Bar graphs showing FPKM of specific genes of interest (for G-H, 3 cell lines; BT2, CT1, CT3; n=3 replicates for each line in each condition over 3 passages, except BT2 at 20% O_2 n=2). **I., J.** ConsensusPathwayDB analysis of Cluster 1 and Cluster 2 from Figure 1H, identifying TF targets with significance from each cluster. **K.** Western blot comparing pluripotent stem cell (PSC) that don't express placental markers, with hTSC and differentiated EVT and STB to highlight specific expression patterns. **L.** Western blot for GCM1 in 2 cell lines grown in 2% and 20% O_2. TFAP2C is the loading control. **M.** Ratio of expression for genes indicated in hTSCs subjected to CRISPRi-targeted degradation of VHL relative to control hTSC. Note reduced expression of GCM1, while known hypoxia target IGFBP3 is dramatically upregulated.

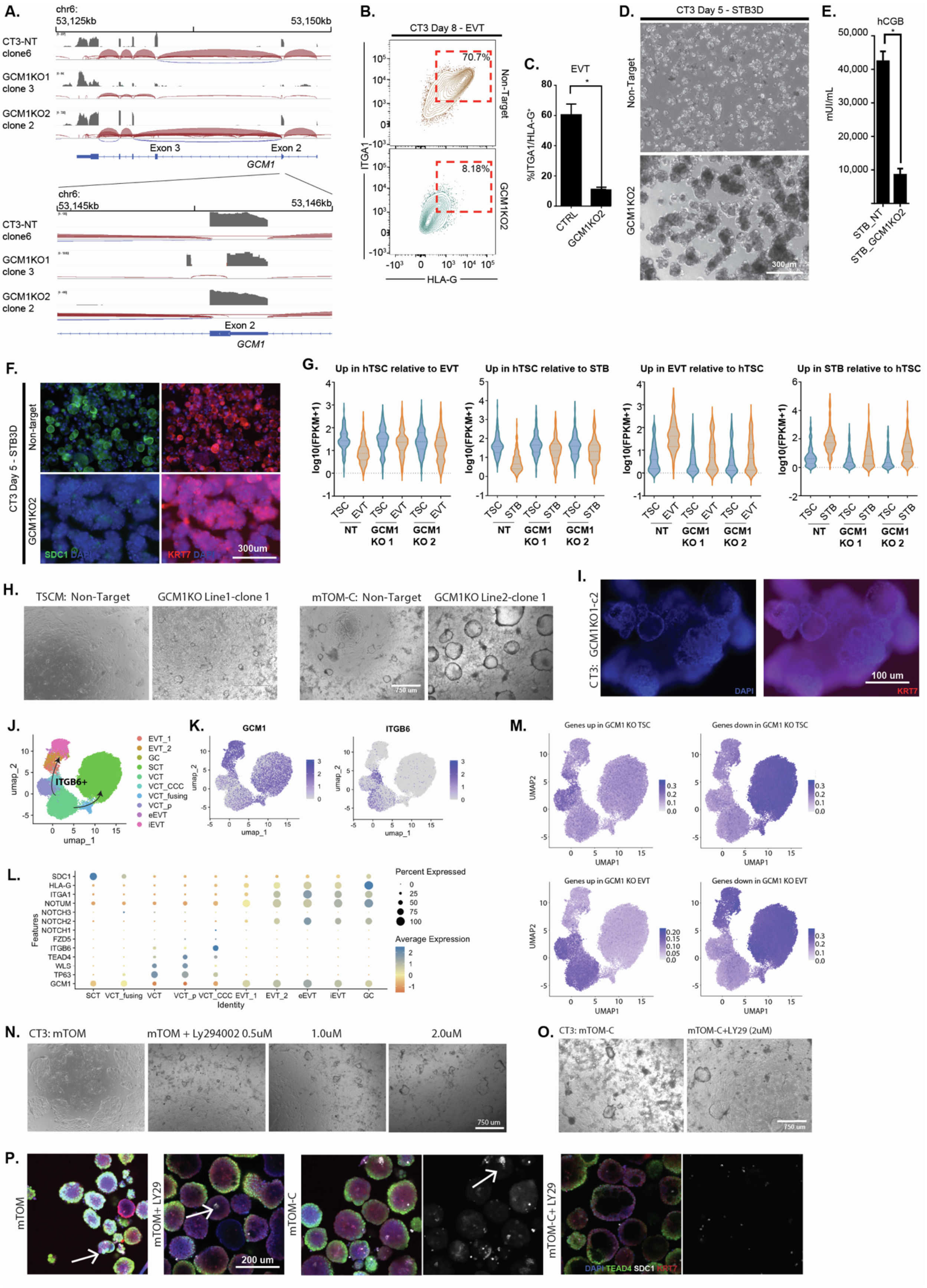
A.
B.
CT3 Day 8 - EVT
C.
EVT
D.
CT3 Day 5 - STB3D
E.
hCGB
F.
G.
Up in hTSC relative to EVT
Up in hTSC relative to STB
Up in EVT relative to hTSC
Up in STB relative to hTSC
H.
TSCM: Non-Target
GCM1KO Line1-clone 1
mTOM-C: Non-Target
GCM1KO Line2-clone 1
I.
J.
K.
M.
Genes up in GCM1 KO TSC
Genes down in GCM1 KO TSC
Genes up in GCM1 KO EVT
Genes down in GCM1 KO EVT
L.
N.
CT3: mTOM
mTOM + Ly294002 0.5uM
1.0uM
2.0uM
O.
CT3: mTOM-C
mTOM-C+LY29 (2uM)
P.

Figure S2 (related to Figure 2). Impaired differentiation upon genetic or chemical reduction in GCM1 level. A. Sashimi plot across the genomic region of *GCM1*. Representative clones were chosen. Normal splicing is observed from non-target line. *GCM1*$^{-/-}$ KO1 (from right to left) show a deletion at the distal tip of exon 2 but an alternative slice site forming just after. *GCM1*$^{-/-}$ KO2 shows the complete skipping of exon 3. **B.** Flow cytometric analysis from EVT differentiation of *GCM1*$^{-/-}$ KO2 and NT hTSC. NT cells differentiation produce ITGA1^{hi}/HLA-G^{hi} cells whereas *GCM1*$^{-/-}$ TSC do not. (representative flow related to C). **C.** Bar graph showing formation of ITGA1^{hi}/HLA-G^{hi} EVTs from control and *GCM1*$^{-/-}$ KO2 hTSC (n=3 clonal replicates for NT and KO). **D.** STB3D formation of NT and *GCM1*$^{-/-}$ KO2 hTSC. Control hTSCs form a fluid-filled syncytium while *GCM1*$^{-/-}$ form a cluster of cells (representative image related to E). **E.** hCGB ELISA was perform using supernatant from *GCM1*$^{-/-}$ and control hTSC (n=3 clonal replicates for NT and KO). **F.** Control and *GCM1*$^{-/-}$ KO2 STB3D stained for the STB-marker SDC1 and the pan-placental marker CKT7. Note absence of SDC1 in *GCM1*$^{-/-}$ (representative image related to E). **G.** Violin plot showing expression of genes specific to hTSC, EVT or STB in cell types indicated. Differentiated *GCM1*$^{-/-}$ cells fail to express differentiation markers and retain expression of TSC markers instead (data analyze from Fig. 2G). **H.** *GCM1*$^{-/-}$-hTSC were grown in mTOM with and without CHIR99021. Dome-like projections appeared in regions of high cell density (representative image line CT3, n=7 each for NT and KO line CT1 and CT3). **I.** Immunofluorescent staining of *GCM1-/-* trophoblast organoids. **J.-L** Reanalysis of Arut. *et al.* 2022 single RNA-seq profiling the several subtypes found in early villus of the placenta. **J.** Cell types in placenta, with path of differentiation indicated. (GC=Giant Cell, VCT = villous CTB, VCT_p=proliferating CTB, VCT_CCC = cell column CTB eEVT=endovascular EVT, iEVT= interstitial EVT). **K.** Expression of GCM1 and ITGB6 in cells shown in (**J**). **L.** Expression of genes indicated in cell types indicated. Note that ITGB6 is associated with cell column cytotrophoblast. **M.** Average expression of gene set indicated ($\log_2$ FC>0.5, $p_{adj} < 0.05$) over cells shown in (**J**). **N.** hTSC cultured in mTOM media with varying concentrations of LY294002. **O.** hTSC cultured in mTOM-C with or without LY294002 2μM. **P.** day14 TB-ORG grown in mTOM or mTOM-C with or without LY294002 2μM treatment and IF stained for DAPI, TEAD4, SDC1, and KRT7. Arrows mark areas of SDC1 expression (n=3 over 3 passages, cell line CT3). Note hollow cavities and lack of SDC1^{+} regions in the presence of LY294002.

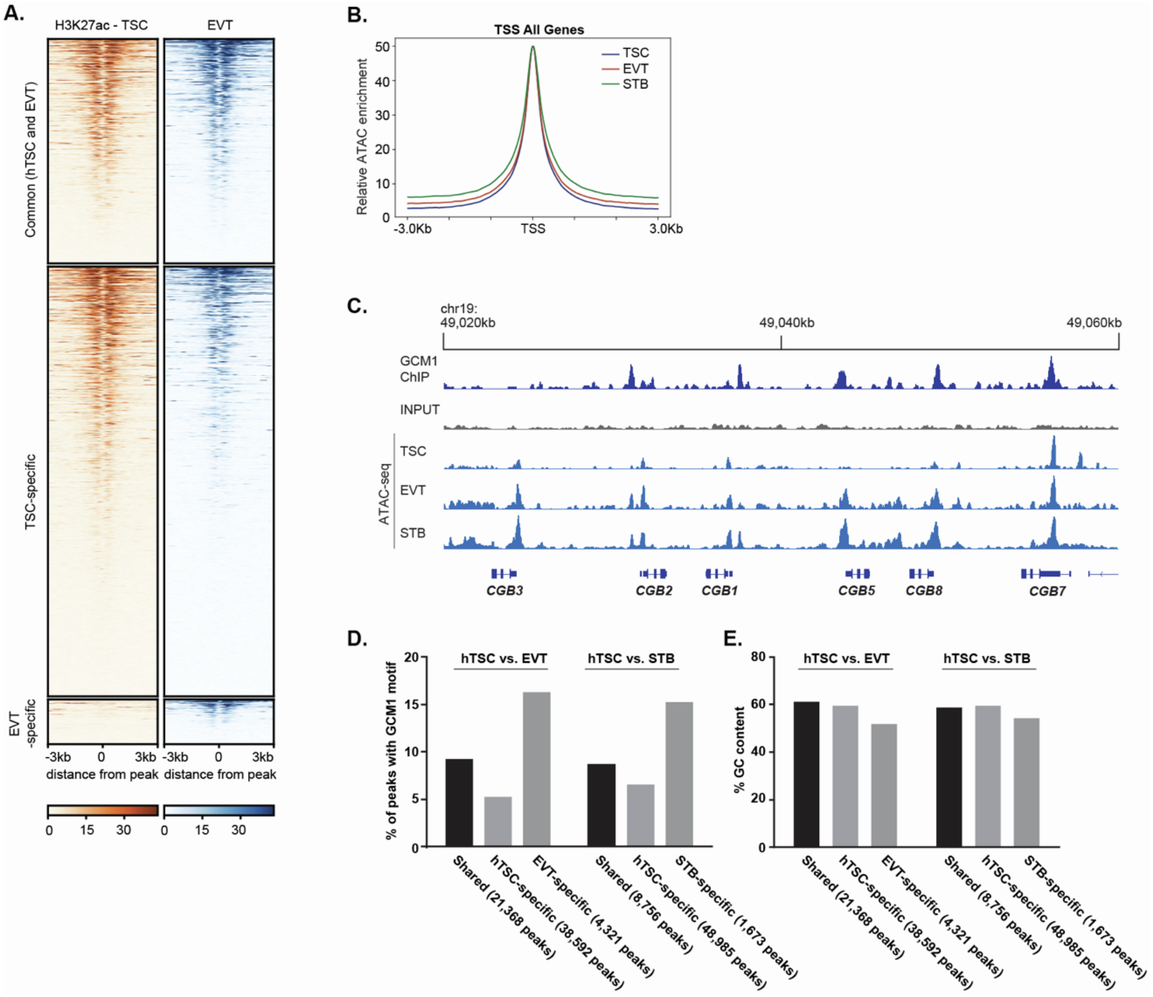

Figure S3 (related to Figure 3). GCM1 positively regulates differentiation-associated genes. **A.** Heatmap of H3K27Ac enrichment over common, hTSC-specific, and EVT-specific ATAC-seq peaks in hTSC and EVT. Note correspondence of H3K27Ac enrichment with ATAC enrichment in each set. **B.** Metaplot of ATAC-seq data from TSC, EVT and STB over all gene TSS after normalization. **C.** GCM1 ChIP-seq and ATAC-seq data plotted over the *CGB* locus. **D.** Percentage of peaks in each category containing GCM motifs. **E.** GC content of ATAC-seq peaks in each category. GCM1 has a GC-rich motif, but the higher frequency of GCM1 sites observed in **(D)** cannot be explained by difference in underlying GC richness.

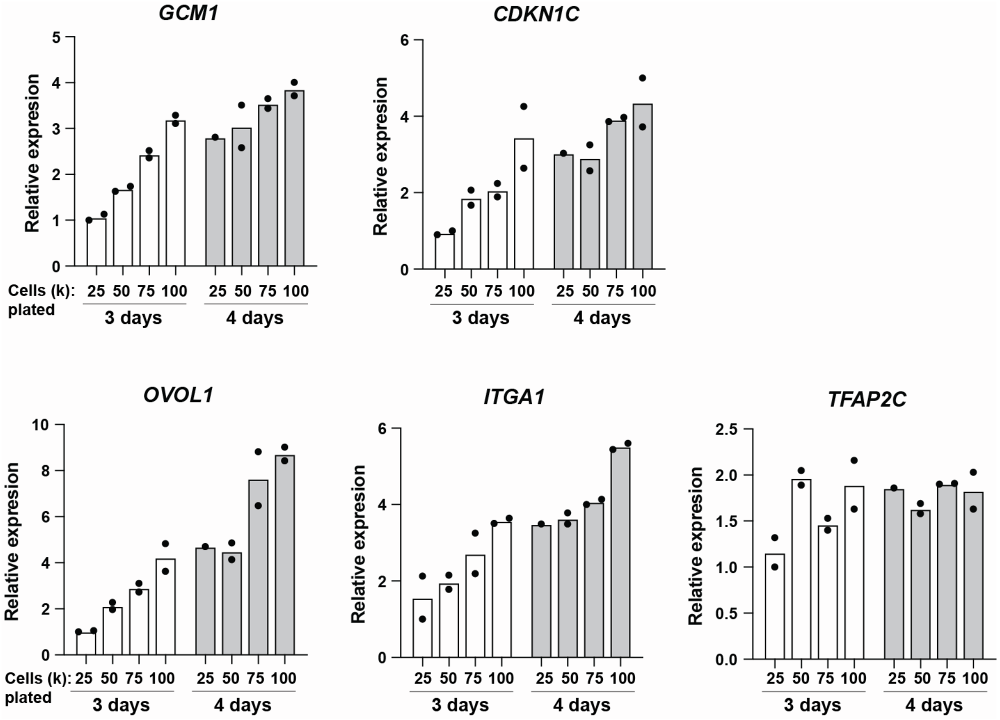

Figure S4 (related to Figure 4). Expression of GCM1 is confluence dependent. Expression of genes indicated in plating conditions (cell number and growth time) indicated. Note that plating at higher densities leads to higher expression of *CDKN1C, GCM1*, *OVOL1* and *ITGA1* (CT3 cells, n=2 replicates).

Supplemental Methods

Cell Culture-Maintenance of hTSC: TSC were cultured in TSC basal media containing DMEM F-12 (GIBCO), 1x ITS-X (GIBCO), 0.3% BSA (WISENT), 1% Penicillin/Streptomycin (GIBCO), 1% ESC qualified Fetal Bovine Serum (GIBCO), 0.1mM β-mercaptoethanol (GIBCO), 15μg/ml L-ascorbic acid (SIGMA), and 50ng/ml recombinant hEGF (GIBCO). To this, 0.75mM valporic acid, 2μM CHIR99021 (Cayman Chem), 0.5μM A8301 (Cayman Chem), 1μM SB431542 (Cayman Chem), and 5μM Y27632 (Cayman Chem) was added to make TSC Media (TSCM) immediately before use. TSCs were dissociated using TrypLE (GIBCO) diluted with PBS to 30% and incubated at 37°C for 10 min. TrypLE was deactivated using 1:1 vol of 0.5mg/ml Soybean trypsin inhibitor (GIBCO) diluted in PBS. For experiments performed in Figure 1, hTSCs were passaged on to 5μg/ml of Collagen IV (Corning) coated plates, but due to product availability, TSC from experiments in Figures 2-4 were passaged onto Laminin 511(SIGMA) coated plates.

Cell Culture-Differentiation of hTSC to EVT: TSCs were converted to EVTs using a modified TSC basal media containing DMEM F-12 (GIBCO), 1x ITS-X (GIBCO), 0.3% BSA (WISENT), 1% Penicillin/Streptomycin, and 0.1mM β-mercaptoethanol (GIBCO). For days 1-2, TSCs were passage onto 1μg/ml Collagen IV coated plates in mod. TSC basal with added 5% Knockout-serum replacement (KSR) (GIBCO), 5μM Y27632 (Cayman Chem), 3μM A8301 (Cayman Chem), 100ng/ml NRG1 (NEB) and 2% GFR-Matrigel (Corning) while the media is still cold (EVTM). Days 3-5, the media is changed to ETVM without NRG1 and reduction to 0.5% GFR-Matrigel. Days 6-8, the media is changed to the previously mentioned ETVM but this time without NRG1, KSR, and reduction to 0.5% GFR-Matrigel. At the end of day 8, differentiated EVTs can be assessed by Flow cytometry.

Cell Culture-Differentiation of hTSC to STB: TSCs were converted to STB3Ds using a modified TSC basal media containing DMEM F-12 (GIBCO), 1x ITS-X (GIBCO), 0.3% BSA (WISENT), 1% Penicillin/Streptomycin, and 0.1mM β-mercaptoethanol (GIBCO). For days 1-2, TSCs were passage on to suspension plate (Sarstedt) in modified TSC basal media containing 5μM Y27632, 2μM Forskolin (Cayman Chem), 5% KSR (GIBCO), and 50ng/ml recombinant hEGF (STBM). On day 3, cell clusters were collected and pulsed-spun for 30sec to separate single cells from STB fusion-clusters and replated in STBM. At day 5, STB-3D are ready to be assessed by immunofluorescence and/or hCG- ELISA.

To generate STB2D, the same media was used except that hEGF was omitted. hTSCs were seeded onto tissue culture plates pre-treated with 2.5μg/uL Collagen IV. Media was changed on day 3, and STB2D were analyzed on day 5.

Cell culture – standard maintenance and EVT differentiation of trophoblast organoids: Villous cytotrophoblasts (vCTBs) were isolated (6 – 7th week of gestation, n=3), and 1 x 10^5 cells were embedded in Matrigel and cultured in Advanced DMEM/F12 (Invitrogen) supplemented with media containing 10mM HEPES, 1 x B27 (Gibco), 1 x ITS-X (Gibco), 2mM glutamine (Gibco), 0.05 mg/ml gentamicin (Gibco), 1 μM A8301 (R&D Systems), 50 ng/ml recombinant human epidermal growth factor (rhEGF, R&D Systems), 3 μM CHIR99021, and 5 μM ROCKi (Y27632, Santa Cruz). After the first passaging, ROCKi was omitted. The medium was changed every 2 – 4 days, and TB-ORG were split after 5 – 7 days. To test effects of LY294002 under stemness conditions, TB-ORG of passage 2 were treated with DMSO (vehicle) or 5 μM LY294002 for 10 days. Culture media were changed every 2 – 3 days.

For EVT differentiation, passage 2 TB-ORG were incubated with TB-ORG medium lacking CHIR99021 (TB-ORG-DIFF). To test the effects of LY294002 on EVT lineage formation and differentiation, TB-ORG were incubated in TB-ORG-DIFF medium supplemented with DMSO (vehicle) or 5 µM LY294002 for 10 days. EVT formation was monitored and bright field images were taken every 2 – 3 days.

Flow cytometry: Dissociated single cells were first washed with 1% KSR-PBS solution. Cell samples were incubated with fluorescently conjugated antibodies for 15min at room temperature. Post-incubation, the samples were washed once with 1% KSR-PBS, then resuspended in 1% KSR-PBS containing DAPI nuclear counterstain to identify live or dead cells. Data acquisition was preformed using the LSR Fortessa and data analysis was performed on FlowJo v10. Antibody: EPCAM (Biolegend324239), ITGA1 (Biolegend328313), ITGA6 (Biolegend313631), and HLA-G (ab24384).

Western blot: Dissociated single cell samples were washed with cold PBS and lysed with Laemmli buffer without blue dye and boiled at 95°C for 5 minutes. Protein concentration was determined using a standard Bradford assay. Standard Bio-RAD SDS-PAGE system was used to separate proteins and transfer it to PVDF membrane (Millipore). Membrane blocking, primary and secondary antibody incubations are diluted in Odyssey Blocking buffer (LICOR). Infrared conjugated secondary antibodies were used for detection and visualization of the protein of interest on the membrane with the LICOR-Odyssey Imager. Antibody: GCM1 (ab187860, 49kDA), TFAP2C (ab76007, 49kDA), Histone H3 (ab10799, 15kDA), and TEAD4 (ab58310, 48kDA). Figure 4D was performed with 2 blots of the same protein samples.

Immunofluorescence: Glass coverslips were precoated with 5µg/ml Collagen IV (CORNING) overnight before cell attachment. Cells were grown for a determined amount of time for its corresponding experiment. Coverslips were fixed with 4% PFA for 20 min. at room temperature. PFA solution was washed 3 times with PBS before permeabilization with permeabilization buffer (PBS + 5% donkey serum + 0.1% Trition-X100) for 30 min. Primary and secondary antibodies were diluted in permeabilization buffer and incubated for 1-2 hours for each process. Coverslips were washed with PBS containing DAPI nuclear counterstain and mounted on glass slides using Pro Long Gold (Invitrogen). Imaging analysis was performed using the Axiovert (Zeiss). Antibody: GCM1 (ab187860), hCGB (ab131170) ITGA2 (Biolegend108901), ITGA5 (ab150361), KRT7 (BioLegend601603), NOTCH1 (CS4380), SDC1 (HPA006185), TEAD4 (ab58310), and TFAP2C (ab76007).

RNA isolation and qPCR: Total RNA isolation used manufacturing protocol indicated by Sigma-Aldrich RNAzol^{RT} R4533. Qubit™ RNA BR Assay kit (Q10211) was used to measure RNA concentration.
First-strand cDNA was generated using the SensiFast cDNA Synthesis kit (Froggabio). Quantitative PCR was done using PowerUP SYBR^{TM} Green PCR (Invitrogen, A25742) on Quantstudio 5 (Applied Biosystems) with the following cycling conditions: 50 °C 2 minutes, 95 °C 20 seconds, 45x (95 °C 3 seconds, 60 °C 30 seconds), 95 °C 1 second). The qPCR reaction was performed using 1x concentration of PowerUP SYBR Green Master Mix, 5ng of template mRNA and 0.5 µM of primer mix in a total of 7 µL reaction. The expression of target genes was

normalized to the housekeeping gene *RAB7A* and the sequence of primer used for qPCR are provided in the table below.

qPCR primer name	sequence
GCM1_F	TGAACACAGCACCTTCCTCC
GCM1_R	CGCCTTCCTGGAAAGACCAA
RAB7A_3_F	GAGGTGGAGCTGTACAACGAATTT
RAB7A_3_R	CGGTCATTCTTGTCCAGTTTGATAG
CDKN1C (p57KIP2)_F	AGCTGCACTCGGGGATTTC
CDKN1C (p57KIP2)_R	ACTTCTCAGGCGCTGATCTCTT
OVOL1_1F	CAATGACACCTTCGACCTCA
OVOL1_1R	TGCACACCATGGATCTTCTT
ITGA1_F	ACGCTGCTGCGTATCATTCA
ITGA1_R	CACCTCTCCCAACTGGACAC
TFAP2C_F	CGCGGAAGAGTATGTTGTTG
TFAP2C _R	TATGTTCGGCTCCAAGACCT
VHL_F4_CR	GACCTGGAGCGGCTGACA
VHL_R4_CR	TACCATCAAAAGCTGAGATGAAACA

RNA isolation and qPCR from TB-ORG (Figures 2N,O). TB-ORG were washed with ice-cold PBS and re-suspended with PeqGold Trifast (PeqLab). Homogenization of TB-ORG was supported using the Precellys 24 (CK-Mix tubes, 5000 rpm, 1 x 20 sec, PeqLab) and RNA isolation was performed as indicated by the manufacturer. RNA (1 µg per sample) was reverse transcribed (RevertAid H Minus Reverse Transcriptase, Thermo Scientific) and qPCR was performed (7500 Fast Real-time PCR system, Applied Biosystems). The following TaqMan Gene Expression Assays (ABI) were used: *CGB* (Hs00361224_g), *ENDOU* (Hs00195731_m1), *TP63* (Hs00978340), *HLA-G* (Hs00365950_g1), and *ITGA1* (Hs00235006_m1). Signals (ΔCt) were normalized to TATA-box binding protein (*TBP*, 4333769F).

Library preparation: For RNA library synthesis, in brief, purified mRNA was cleaned using the NEBNext Poly(A) mRNA Magnetic Isolation Module kit (NEB E7490) and final library generation was created with the NEBNext® Ultra RNA Library Prep Kit for Illumina® (NEB E7530) following manufacturing instructions. Barcoding came from TruSeq Unique Dual Indexes (Illumina, San Diego, CA). Qubit™ 1x dsDNA HS Assay kit (Q33231) was used to measure synthesis of the library.

Chromatin immunoprecipitation: Cell samples are fixed with 0.66% paraformaldehyde (FisherSci) diluted with PBS and is quenched by adding glycine to a final concentration of 0.125M. Nuclear lysis extraction begins with nuclear lysis buffer (50mM HEPES pH 7.8, 0.5% Triton X-100, 1mM EDTA, 0.5mM EGTA, 140 mM NaCl, 10% glycerol and 1% NP-40). Nuclei are resuspended in nuclear wash buffer (10mM Tris-HCl pH 8.0, 200mM NaCl, 1mM EDTA, and 0.5mM EGTA). Nuclear pellets are then resuspended in SDS lysis buffer (50mM Tris-HCl pH 8, 10mM EDTA, 1% SDS, and 1% Triton X-100). Nuclei samples are transferred to a 1ml tube (Bioruptor) and keep cold before sonication. Sonication was performed using the Diagenode Bioruptor sonicator (settings: 30sec on, 30 sec off, 30-35 cycles). Then diluted with Dilution buffer (25mM Tris-HCl pH 8, 150 mM NaCl, 3mM EDTA, and 1% Triton X-100). A pre-clearing step is performed by using 40µl of pre-washed Protein G Sepharose beads (SIGMA, P3296-5ml)

combined sonicated sample. 2μg of GCM1(HPA001343) antibody is added to each pre-cleared sample and then rotated overnight at 4°C. Pre-washed Protein G Sepharose beads are added to the ChIP samples and washed with three buffers: DB150 (25mM Tris-HCl pH 8.0, 150mM NaCl, 3mM EDTA, 1% Triton X-100, and 0.05% SDS), DB500 (25mM Tris-HCl pH 8.0, 500mM NaCl, 3mM EDTA, 1% Triton X-100, and 0.05% SDS), Buffer III (10mM Tris-HCl pH 8.0, 250mM LiCl, 1% Sodium Deoxycholate, 1% NP-40, and 1mM EDTA, and lastly TE buffer (10mM Tris-HCl pH 8.0, 1mM EDTA). DNA is eluted with 200µl elution buffer (100 mM NaHCO3, and 1% SDS) and incubated at 65°C overnight for decrosslinking. Ethanol precipitation is performed to collect ChIP material, and further purification was performed with Geneaid Gel/PCR cleanup protocol. Purified DNA fragments passed to sequencing library preparation.

ATAC-Seq: ATAC-seq library preparation was performed on 1×10^6 freshly cultured cells using a commercially available ATAC-Seq kit from Active Motif (#53150, Carlsbad, CA). We followed manufacturers protocol with some minor modification. Each sample was lysed in the ATAC-seq lysis buffer. Next, the samples were processed for the transposase reaction. After cleanup of the transposed DNA, samples were stored at −20 °C until library amplification. Samples were subsequently thawed at room temperature and library construction completed according to manufacturer protocol. Libraries were quantified using a Qubit dsDNA BR Assay Kit (Q32853, Thermo-Fisher) and the size was determined with a High Sensitivity DNA Bioanalyzer Kit (5067-4626, Agilent, Santa Clara, CA) and sequenced on a NovaSeq 6000 (Illumina, San Diego, CA) using Nextera Sequencing primers.

Supplemental Table Captions

Table S1: Gene expression associated with different oxygen concentrations, including FPKM of all samples, differentially expressed genes for 2% and 5% O_2 as compared with 20% O_2, and gene cluster analysis to identify genes associated with O_2 level.
Table S2: Expression of genes specific to hTSC, EVT and STB in hTSCs grown at 20%, 5% and 2% O_2.
Table S3: Gene expression associated with WT and GCM1 KO lines, including FPKM, expression of hTSC, STB and EVT-specific genes, and genes identified as differentially expressed between WT vs. GCM1 KO.
Table S4: GCM1 ChIP-seq peaks as well as ATAC-seq peaks specific to cell types indicated.
